# Supplementary material for: An amide to thioamide substitution improves the permeability and bioavailability of macrocyclic peptides
Source: Nat Commun. 2023 Sep 28;14:6050. doi: 10.1038/s41467-023-41748-y (PMC10539501; doi:10.1038/s41467-023-41748-y)
Supplement: Supplementary file 1 — Supplementary Information [file 41467_2023_41748_MOESM1_ESM.pdf]

SUPPLEMENTARY INFORMATION

An amide to thioamide substitution improves the permeability and bioavailability of macrocyclic peptides

*Pritha Ghosh[1], Nishant Raj[1], Hitesh Verma[1], Monika Patel[2,3], Sohini Chakraborti[1], Bhavesh Khatri[1], Chandrashekar M. Doreswamy[4], Anandakumar S. R. [4], Srinivas Seekallu[4], Dinesh M. B.[5], Gajanan Jadhav[6], Prem Narayan Yadav[2,3], and Jayanta Chatterjee[*1]*

[1]Molecular Biophysics Unit, Indian Institute of Science, Bangalore 560012, Karnataka, India
[2]Neuroscience & Ageing Biology, CSIR-CDRI, Lucknow 226031, Uttar Pradesh, India
[3]Academy of Scientific and Innovative Research (AcSIR), Ghaziabad 201002, India
[4]Department of Pre-clinical Research, Anthem Biosciences Pvt. Ltd., Bangalore 560099, Karnataka, India
[5]Central Animal Facility, Indian Institute of Science, Bangalore 560012, Karnataka, India
[6]Eurofins Advinus Biopharma Services India Pvt. Ltd., Bangalore 560058, Karnataka, India

*Corresponding author:

Prof. Jayanta Chatterjee, Molecular Biophysics Unit, Indian Institute of Science.
C. V. Raman Avenue, Bangalore 560012, Karnataka, India.
Tel: 080-2293 2053, E-mail: jayanta@iisc.ac.in

Table of Contents

Supplementary Figures……………….……………………………………………………..S3-S21

Supplementary Tables…………………………………………………………………S22-S25

Supplementary Materials and Instrumentation…………………………………………S26

Supplementary Methods and Protocols………………………………………………...S27-S36

ESI-MS and HPLC purification profiles of cyclic peptides **10** and **10a-k**……………S37-S48

ESI-MS and HPLC purification profiles of cyclic peptides **11** and **11a-g**……………S49-S56

ESI-MS and HPLC purification profiles of cyclic peptides **12** and **12a-h**……………S57-S65

ESI-MS and HPLC purification profiles of cyclic peptides **13** and **13a-f**…………….S66-S72

ESI-MS and HPLC purification profile of peptide control (**PC**)………………………S73

^{1}H, TOCSY, and ROESY spectra of **10**, **10a**, **10e**, and **10f** in DMSO-d_6…………....S74-S78

Absolute proton chemical shifts and $^3J_{NH}$ of **10**, **10a**, **10e**, and **10f** in DMSO-d_6…...S79

Distance violations of **10**, **10a**, **10e**, and **10f** in DMSO-d_6……………………………S80-S83

^{1}H, TOCSY, and ROESY spectra of **10**, **10a**, **10e**, and **10f** in $CDCl_3$………………..S84-S88

Absolute proton chemical shifts and $^3J_{NH}$ of **10**, **10a**, **10e**, and **10f** in $CDCl_3$……….S89

Distance violations of **10**, **10a**, **10e**, and **10f** in $CDCl_3$………………………………...S90-S93

^{1}H, TOCSY, and ROESY spectra of **13**, and **13a-13f** in DMSO-d_6………………….S94-S101

Absolute proton chemical shifts and $^3J_{NH}$ of **13**, and **13a-13f** in DMSO-d_6…………S102

Distance violations of **13**, and **13a-13f** in DMSO-d_6……………………………………S103-S109

Supplementary References………………………………………………………………S110

a

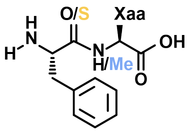

AlogP	Oxo	Thio	N-methyl
1 (FK)	-3.14	-2.05	-2.94
2 (FE)	-2.36	-1.27	-2.15
3 (FG)	-2.55	-1.45	-2.34
4 (FA)	-2.06	-0.97	-1.86
5 (FV)	-1.22	-0.13	-1.01
6 (FM)	-1.73	-0.64	-1.53
7 (FW)	-0.21	0.88	0

b

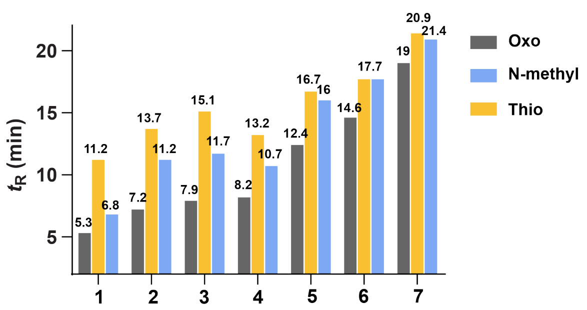

Supplementary Figure 1. Hydrophobicity assessment of dipeptides. AlogP values (**a**) and HPLC retention time in C18 column (**b**) of peptides **1-7** and their N-Me and thio-analogs. (Solvent A: 0.1% TFA in water, solvent B: 0.1% TFA in acetonitrile, gradient 10-60% solvent B over 20 minutes).

a

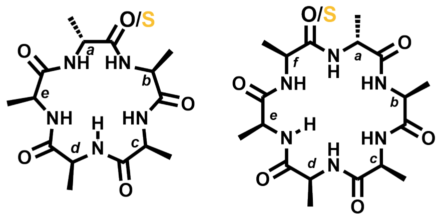

8 9

b

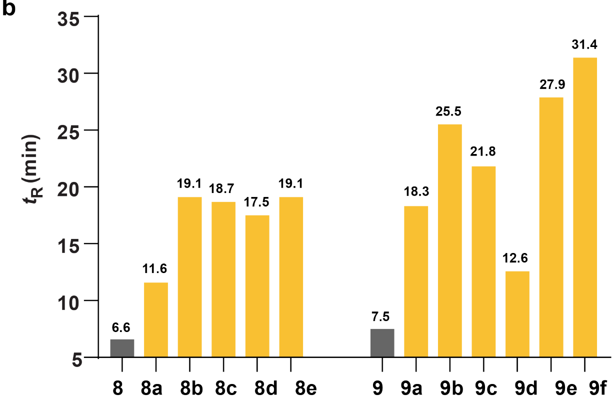

Supplementary Figure 2. Retention time analysis of cyclic penta- and hexa-Ala peptides. a Chemical structure of peptides **8** and **9**. **b** HPLC retention time of **8, 9** and their thio-analogs in C18 column. (Solvent A: 0.1% TFA in water, solvent B: 0.1% TFA in acetonitrile, gradient 10-50% solvent B over 30 minutes).

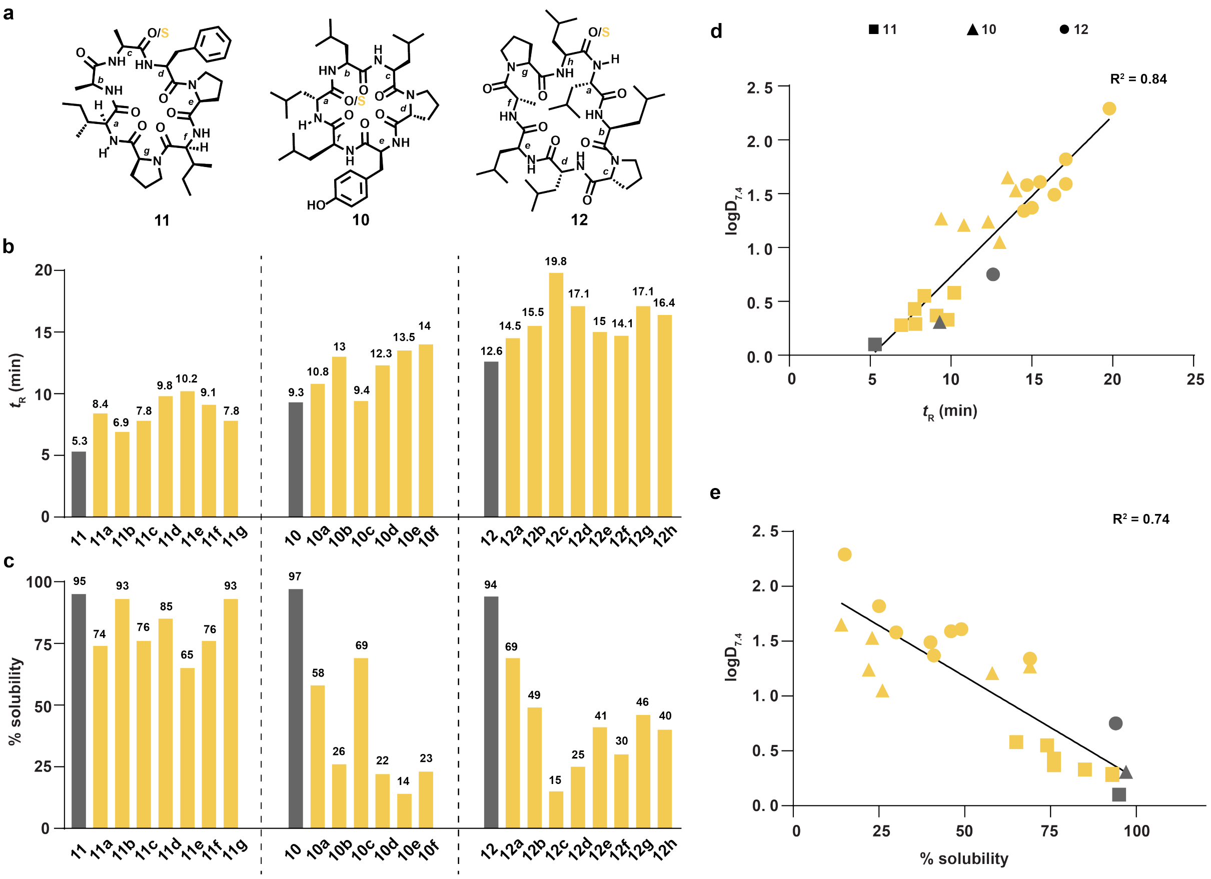

Supplementary Figure 3. Effect of O to S substitution on retention time & solubility of macrocycles and their correlation with $logD_{7.4}$. a Chemical structure of peptides **11, 10,** and **12.** HPLC retention time in C18 column. (**b**) % solubility (**c**) of peptides **10, 11,** and **12** and their thio-analogs. **d** Linear correlation between $logD_{7.4}$ and HPLC retention time of the peptides and their thio analogs. **e** Linear correlation between $logD_{7.4}$ and % solubility of the same group of peptides. (Solvent A: 0.1% TFA in water, solvent B: 0.1% TFA in methanol, gradient 80-100% solvent B over 20 minutes). Source data are provided as a Source Data file.

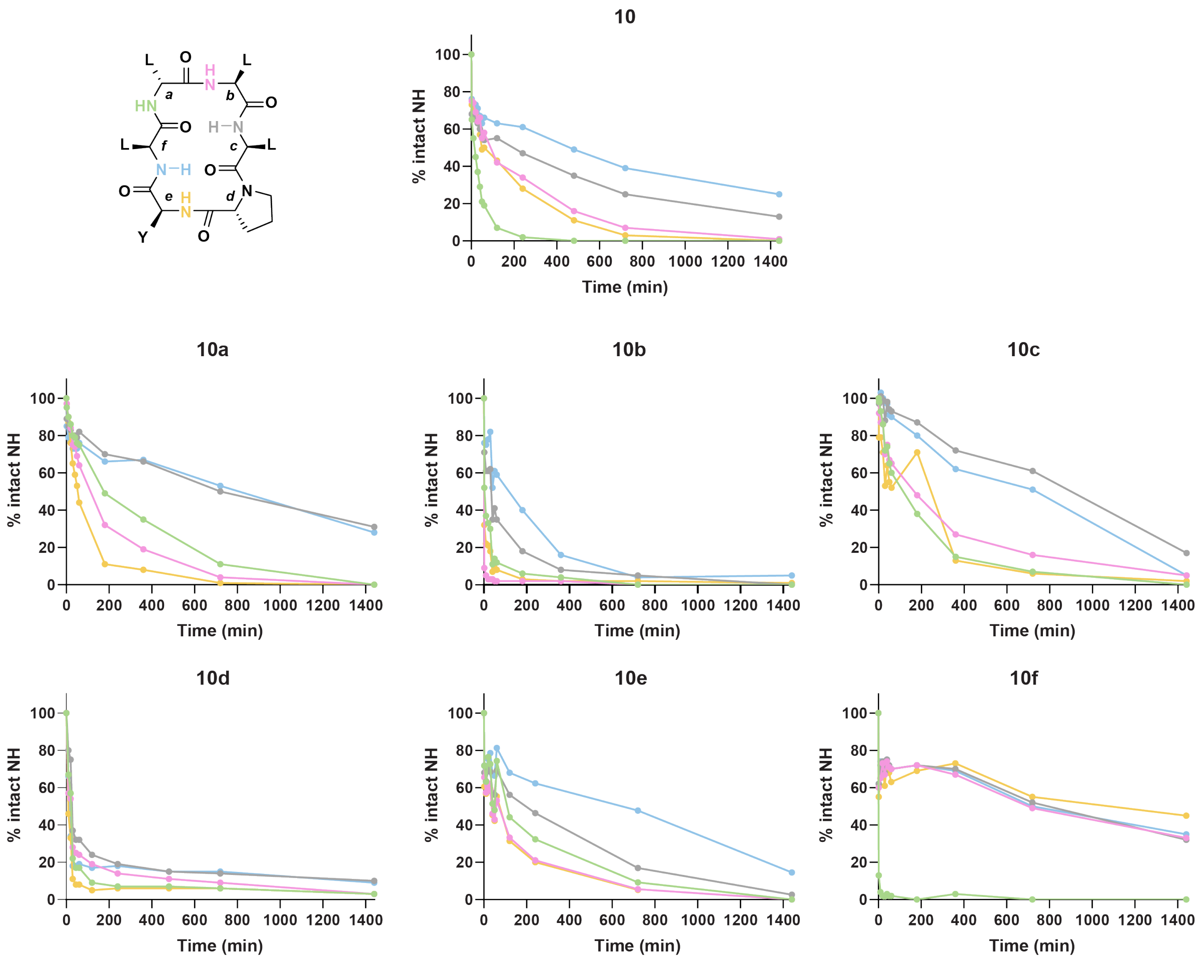

Supplementary Figure 4. Analyses from Hydrogen-Deuterium exchange of macrocycles in DMSO-*d*$_6$. Exchange kinetics of the amide protons of **10**, **10a-f** in 20% D_2O in DMSO-*d*$_6$ over 24 hours. Source data are provided as a Source Data file.

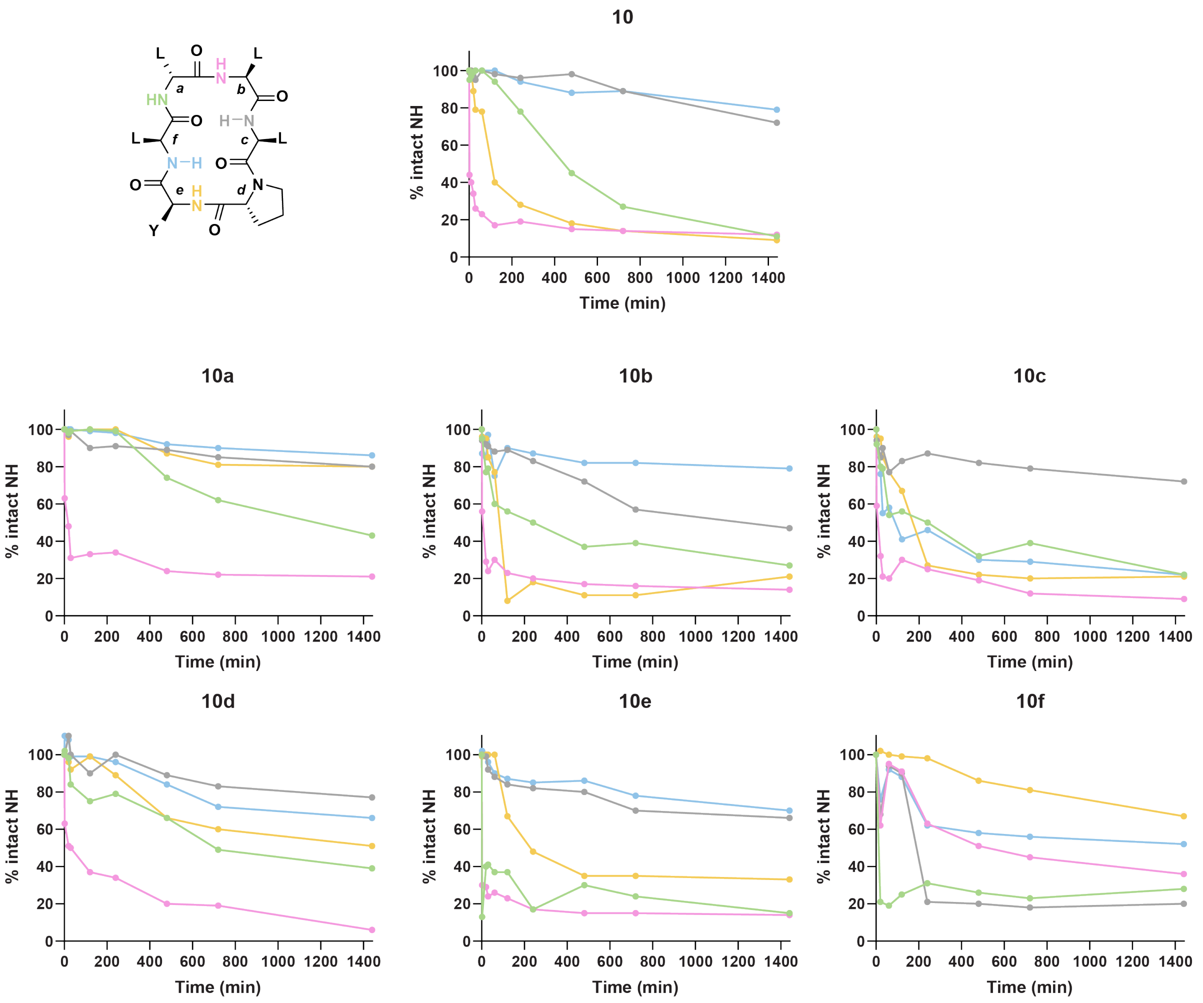

Supplementary Figure 5. Analyses from Hydrogen-Deuterium exchange of macrocycles in $CDCl_3$. Exchange kinetics of the amide protons of **10**, **10a-f** in 20% CD_3OD in $CDCl_3$ over 24 hours. Source data are provided as a Source Data file.

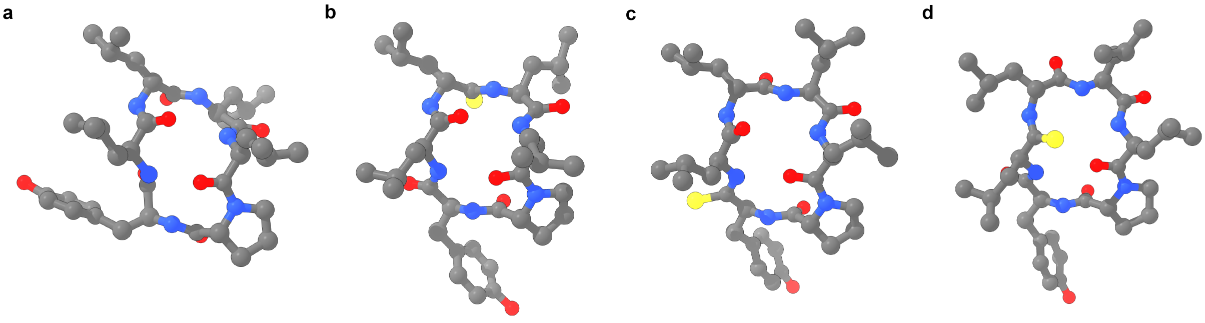

10 10a 10e 10f

Supplementary Figure 6. Conformations of macrocyclic peptides in DMSO-*d*$_6$. Average rMD structures of **10** (**a**), **10a** (**b**), **10e** (**c**), and **10f** (**d**) calculated in DMSO-*d*$_6$. The structures have been generated in ChimeraX.

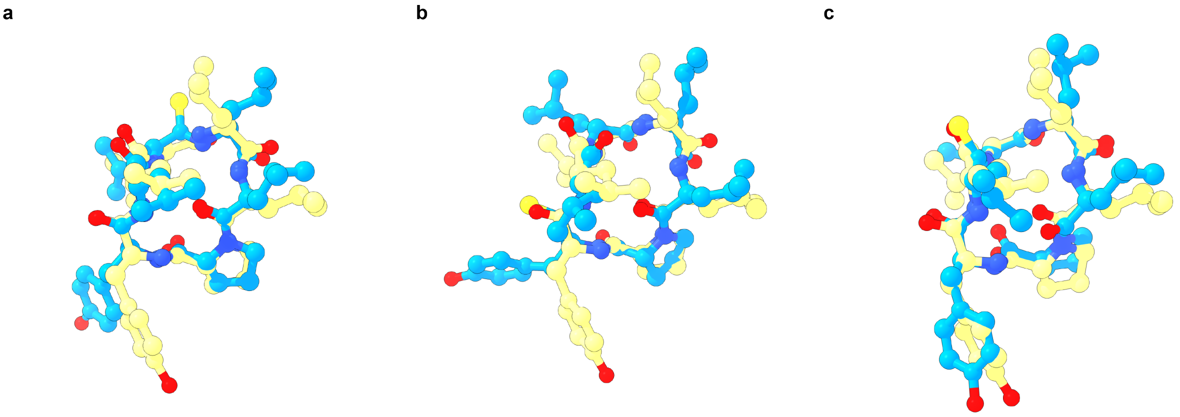

	10a	10e	10f
**RMSD**	**0.716**	**0.851**	**0.413**

Supplementary Figure 7. Structural impact of thioamidation on macrocyclic peptides. Overlay of the average fMD structures (in DMSO-*d*$_6$) of **10a** (**a**), **10e** (**b**), and **10f** (**c**) on that of the parent scaffold **10** (yellow). The RMSDs between six pruned atom pairs (Cα) with respect to **10** in each case has been highlighted at the bottom of each overlaid pair. The structures have been generated in ChimeraX.

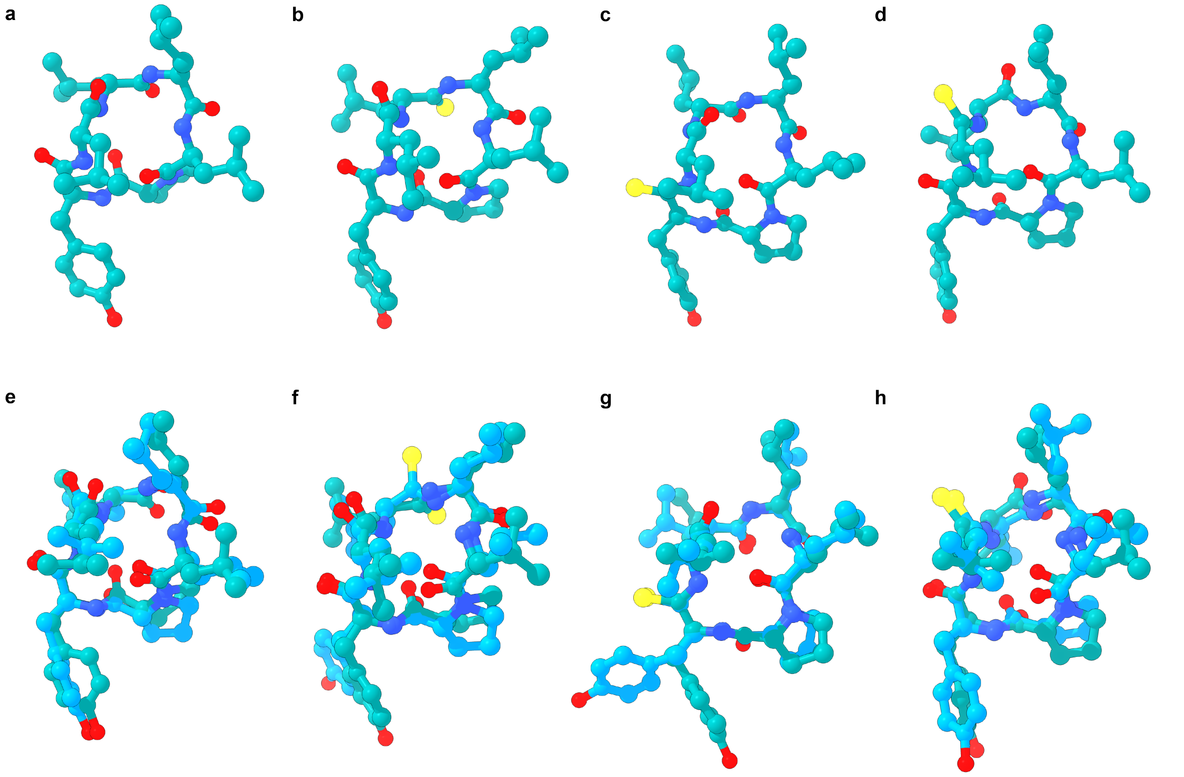

	10	10a	10e	10f
RMSD	0.511	0.356	0.253	0.517

Supplementary Figure 8. Conformations of macrocyclic peptides in DMSO-d_6 and $CDCl_3$. Average fMD structures of **10** (**a**), **10a** (**b**), **10e** (**c**), and **10f** (**d**) calculated in $CDCl_3$. Overlay of the average fMD structures of **10** (**e**), **10a** (**f**), **10e** (**g**), and **10f** (**h**) in DMSO-d_6 (aqua) on that of $CDCl_3$ (teal). The RMSDs between six pruned atom pairs (Cα) in each case have been highlighted at the bottom of each overlaid pair. The structures have been generated in ChimeraX.

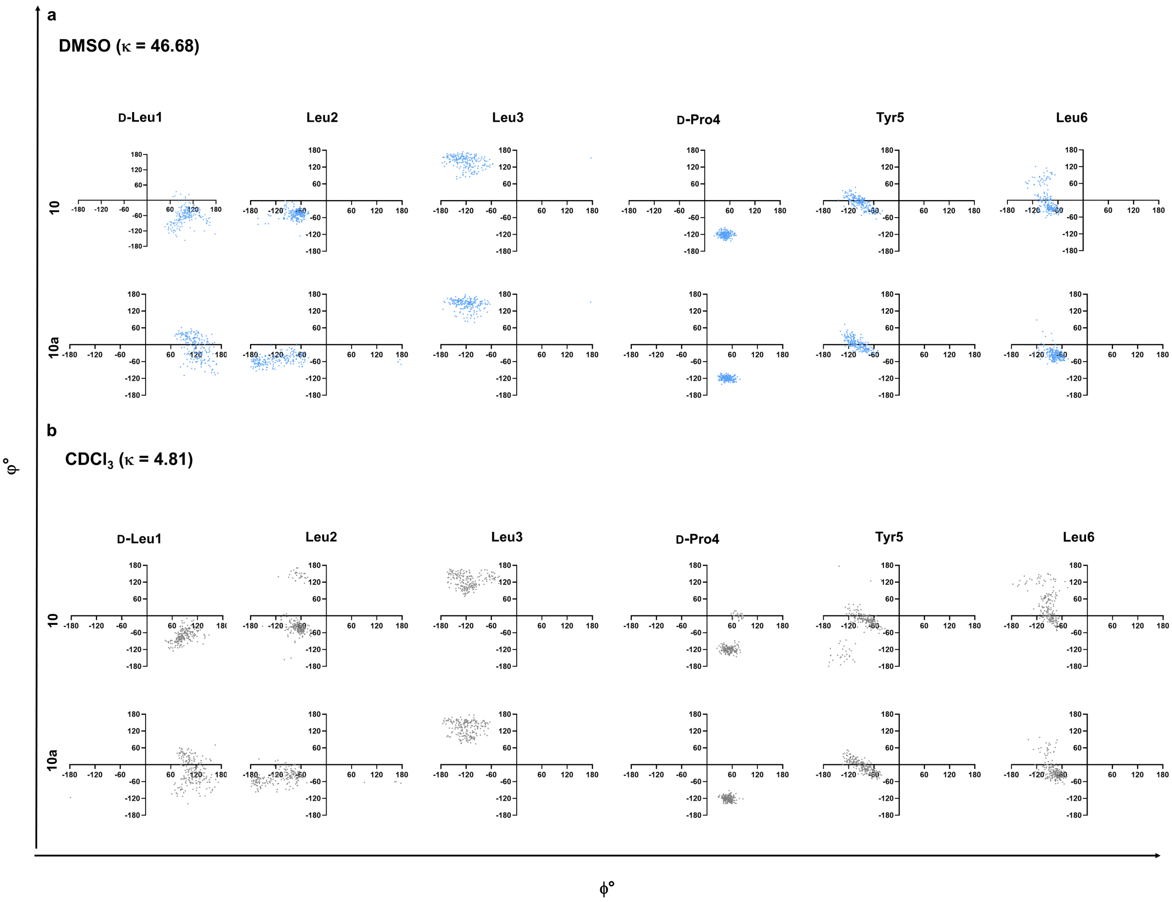

Supplementary Figure 9. ϕ-φ analyses of 10a w.r.t. the parent 10. Ramachandran plot of the ϕ,φ fluctuations in each residue of **10** (**a**, **b**; top panel) vs **10a** (**a**, **b**; bottom panel) during the entire 200 ns fMD simulation in DMSO-d_6 (**a**), and $CDCl_3$ (**b**). Source data are provided as a Source Data file.

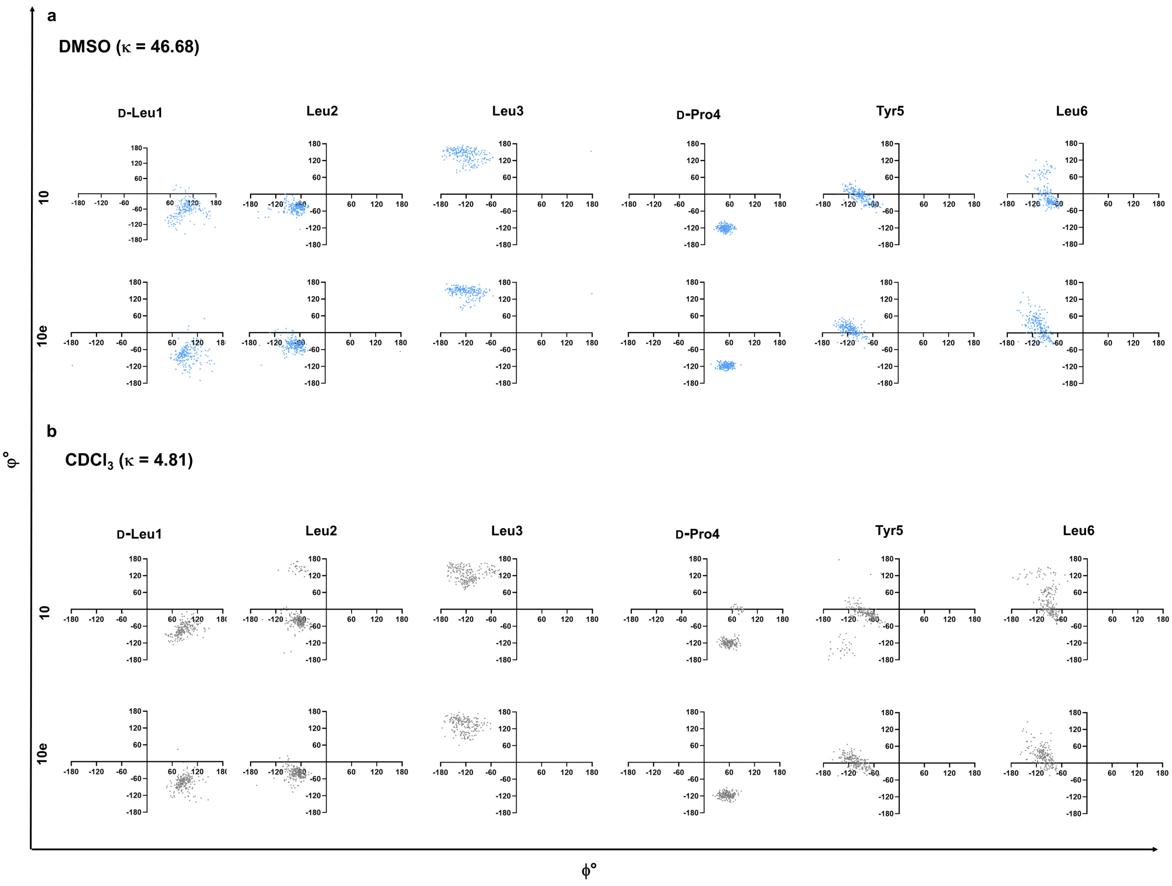

Supplementary Figure 10. ϕ-φ analyses of 10e w.r.t. the parent 10. Ramachandran plot of the ϕ,φ fluctuations in each residue of **10** (**a**, **b**; top panel) vs **10e** (**a**, **b**; bottom panel) during the entire 200 ns fMD simulation in DMSO-d_6 (**a**), and $CDCl_3$ (**b**). Source data are provided as a Source Data file.

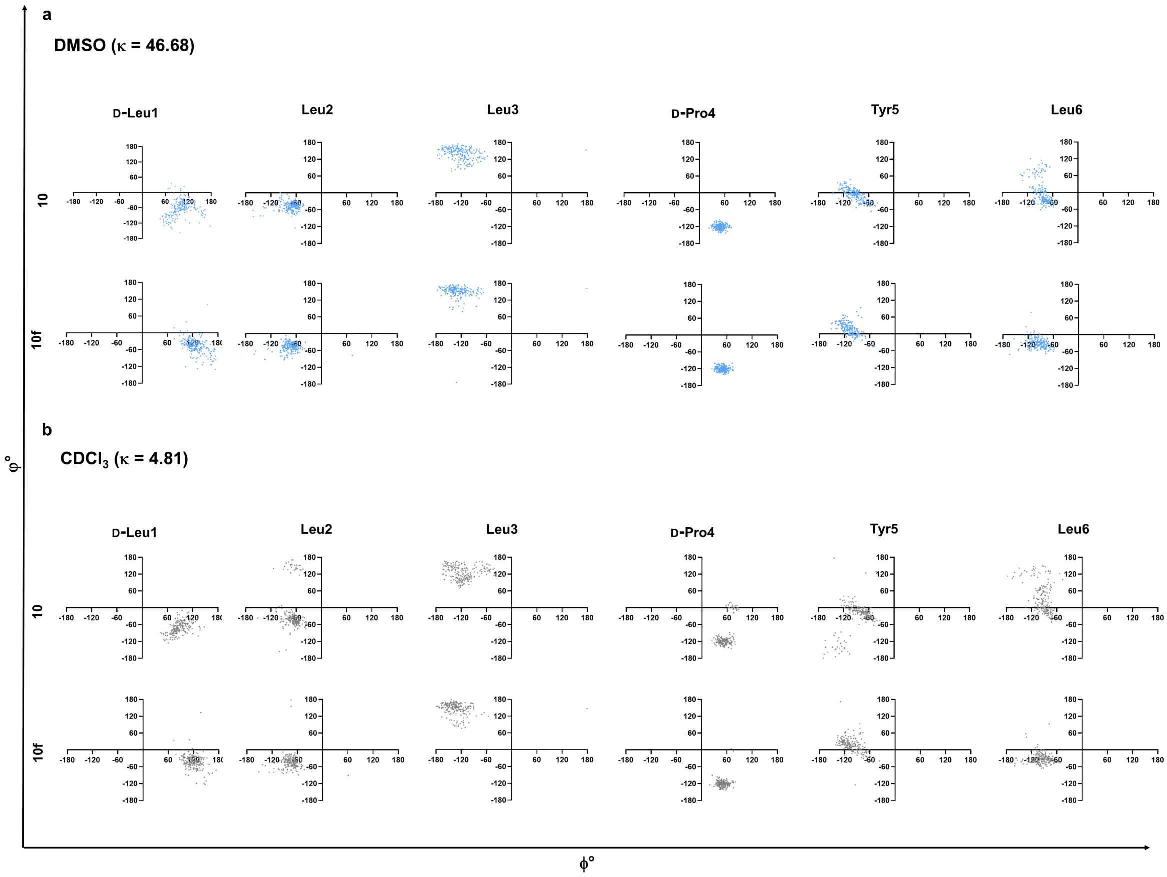

Supplementary Figure 11. ϕ-φ analyses of 10f w.r.t. the parent 10. Ramachandran plot of the ϕ,φ fluctuations in each residue of **10** (**a**, **b**; top panel) vs **10f** (**a**, **b**; bottom panel) during the entire 200 ns fMD simulation in DMSO-d_6 (**a**), and $CDCl_3$ (**b**). Source data are provided as a Source Data file.

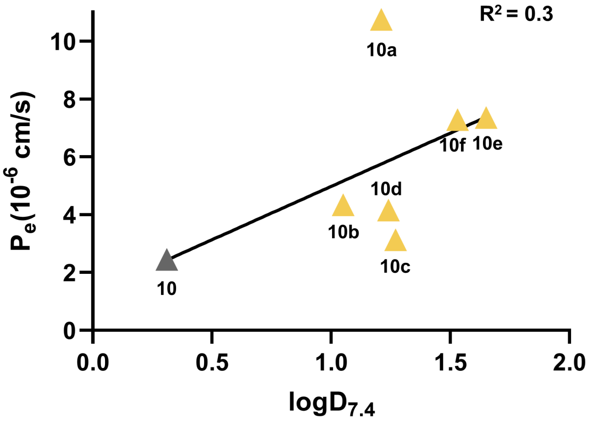

Supplementary Figure 12. Correlation between permeability and lipophilicity of macrocyclic peptides. Linear correlation between $logD_{7.4}$ and PAMPA permeability P_e of **10**, and **10a-f**. Source data are provided as a Source Data file.

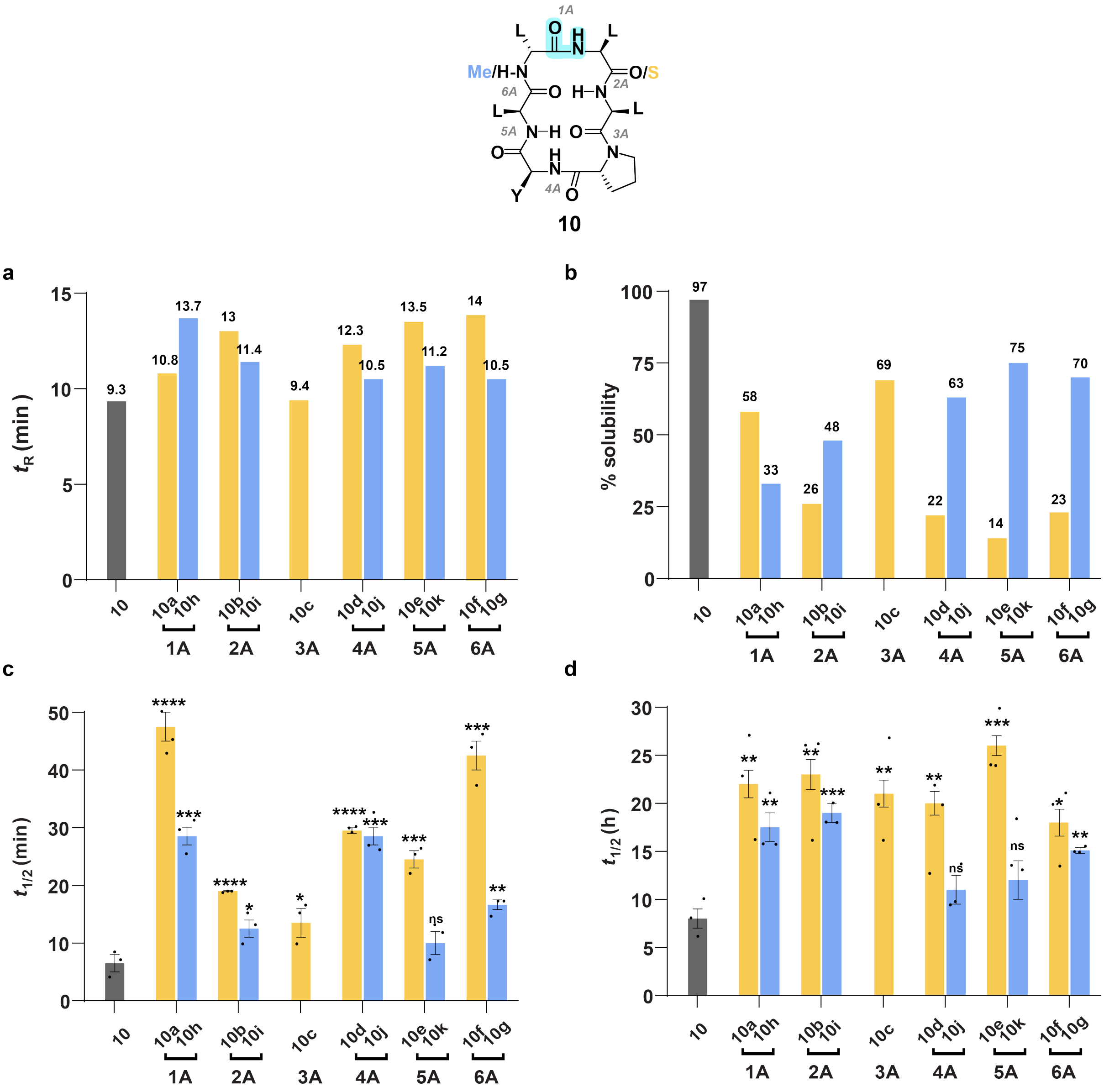

Supplementary Figure 13. Comparison of HBD and HBA-masking on lipophilicity and proteolytic stability of macrocycles. 1A-6A refer to the amide bonds in peptide **10**. The yellow and the blue bars in the individual panels represent the thioamidated (**10a-10f**) and the N-methylated (**10g-10k**) counterparts, respectively, at a specific amide bond. Comparison of the HPLC retention time in C18 column (**a**), % solubility (**b**), half lives in human liver microsome (**c**), and blood plasma (**d**) of the thioamidated and N-methylated analogs of **10** (grey). (Solvent A: 0.1% TFA in water, solvent B: 0.1% TFA in methanol, gradient 80-100% solvent B over 20 minutes). $n = 3 \pm$ SEM. Each bar represents mean values of three biological replicates: dots are individual data points. Statistical significance w.r.t. **10** was measured by a one-tailed unpaired t-test. *$p < 0.05$, **$p < 0.01$, ***$p < 0.001$, ****$p < 0.0001$, ns (non-significant) > 0.05. p (**10** vs **10a**, **10b 10c**, **10d**, **10e**, **10f**) = <0.0001, 0.0005, 0.0223, <0.0001, 0.0005, 0.0002; p (**10** vs **10g**, **10h**, **10i**, **10j**, **10k**) = 0.0021, 0.0004, 0.0216, 0.0006, 0.0919 for in $t_{1/2}$ human liver microsome. p (**10** vs **10a**, **10b 10c**, **10d**, **10e**, **10f**) = 0.0073, 0.0071, 0.0095, 0.0012, 0.0007, 0.0113; p (**10** vs **10g**, **10h**, **10i**, **10j**, **10k**) = 0.0018, 0.0044, 0.0007, 0.0796, 0.0525 for in $t_{1/2}$ human plasma. Source data are provided as a Source Data file.

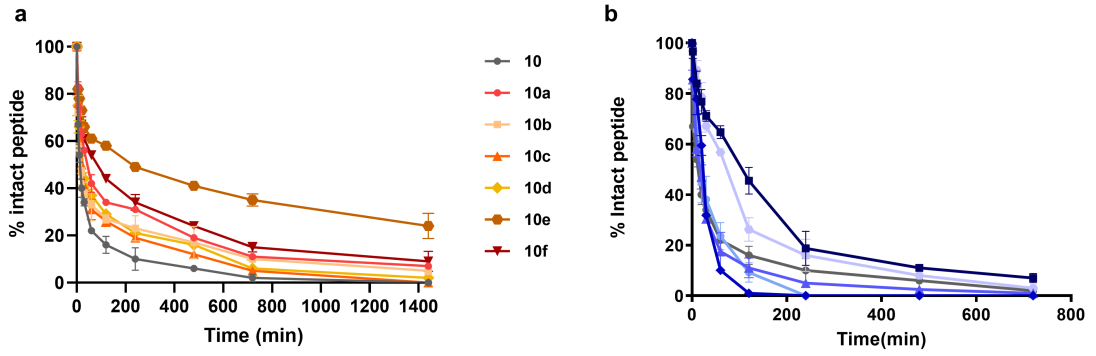

Supplementary Figure 14. Degradation profiles of peptides in SGF. Degradation kinetics of **10**, thioamidated analogs **10a-f** (**a**), and N-methylated analogs **10g-k** (**b**) in simulated gastric fluid. $n = 3 \pm$ SEM. Each point on the graph(s) represents mean values of three biological replicates. Source data are provided as a Source Data file.

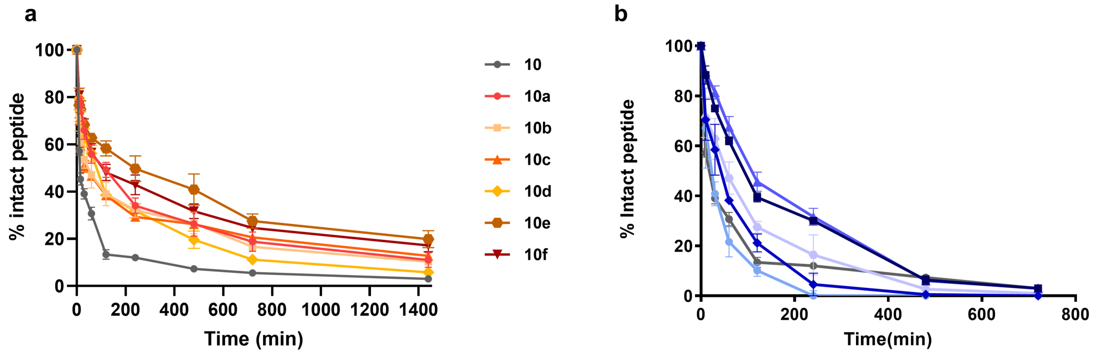

Supplementary Figure 15. Degradation profiles of peptides in SIF. Degradation kinetics of **10**, thioamidated analogs **10a-f** (**a**), and N-methylated analogs **10g-k** (**b**) in simulated intestinal fluid. Each point on the graph(s) represents mean values of three biological replicates. Source data are provided as a Source Data file.

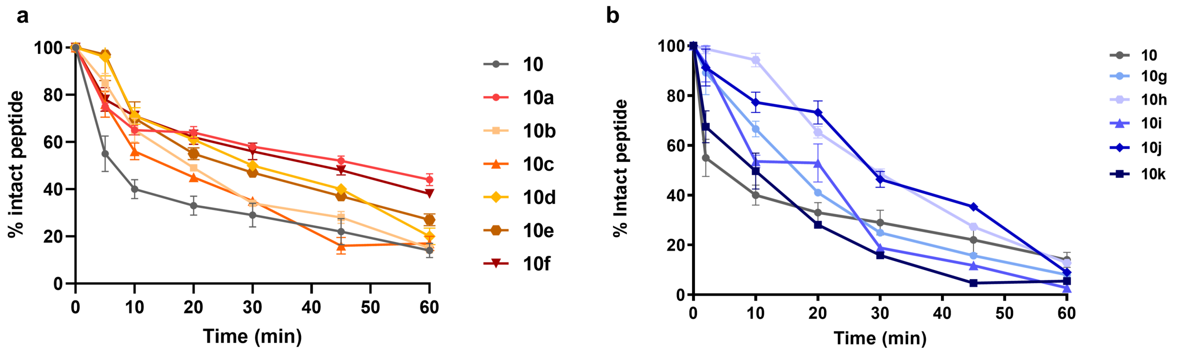

Supplementary Figure 16. Degradation profiles of peptides in human liver microsome. Degradation kinetics of **10**, thioamidated analogs **10a-f** (**a**), and N-methylated analogs **10g-k** (**b**) in human liver microsome. Each point on the graph(s) represents mean values of three biological replicates. Source data are provided as a Source Data file.

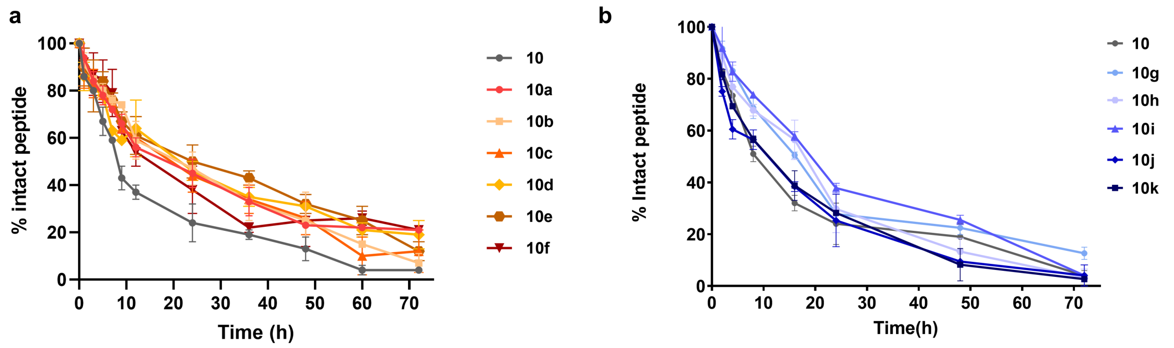

Supplementary Figure 17. Degradation profiles of peptides in plasma. Degradation kinetics of **10**, thioamidated analogs **10a-f** (**a**), and N-methylated analogs **10g-k** (**b**) in human blood plasma. Each point on the graph(s) represents mean values of three biological replicates. Source data are provided as a Source Data file.

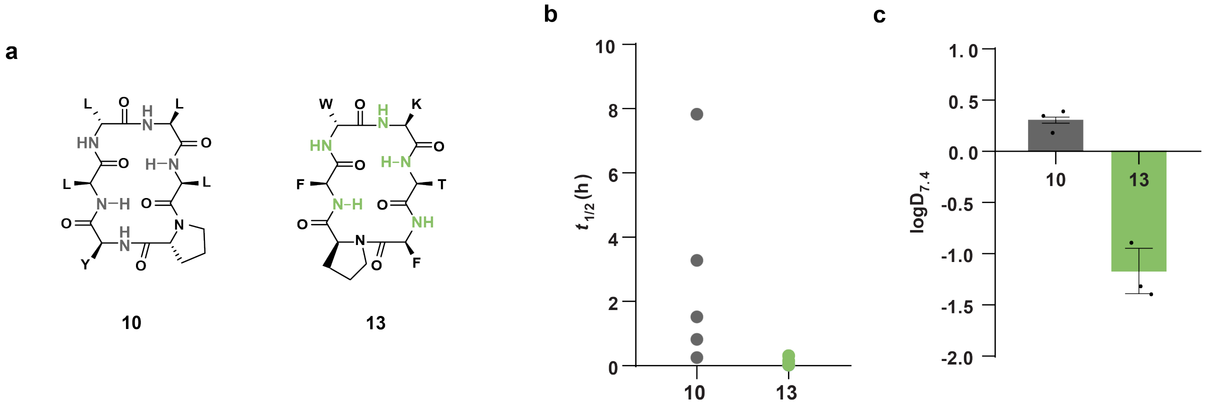

Supplementary Figure 18. Comparison of lipophilicity of cyclic peptide scaffolds 10 & 13. a Chemical structure of scaffolds **10** and **13**. Comparison of the relative solvent shielding and lipophilicity of the scaffolds **10** and **13** through HDX in 20% D_2O in DMSO-d_6. (The bubble plot denotes the half-lives of the amide protons) (**b**) and logD$_{7.4}$ (**c**). n = 3 ± SEM. Each bar represents mean values of three biological replicates: dots are individual data points. Source data are provided as a Source Data file.

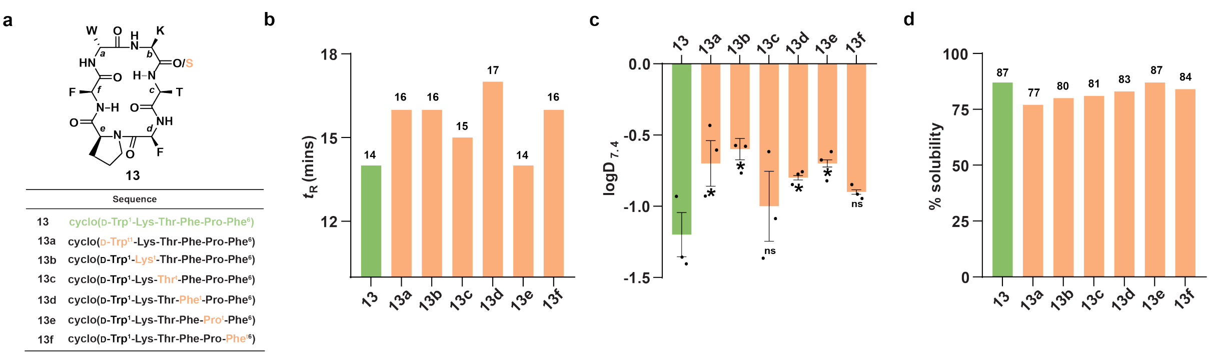

Supplementary Figure 19. Impact of thioamidation on the lipophilicity of macrocyclic peptide 13. a Chemical structure of **13** and sequence mono-thioamidated peptide library of **13**. HPLC retention time in C18 column. (**b**), logD$_{7.4}$ (**c**), and % solubility (**d**) of the thio-analogs in comparison to **13**. Solvent A: 0.1% TFA in water, solvent B: 0.1% TFA in acetonitrile, gradient 30-80% solvent B over 20 minutes. n = 3 ± SEM. Each bar represents mean values of three biological replicates: dots are individual data points. Statistical significance w.r.t. **13** was measured by a one-tailed unpaired t-test. *$p < 0.05$, **$p < 0.01$, ***$p < 0.001$, ****$p < 0.0001$, ns (non-significant) > 0.05. p (**13**

vs **13a**, **13b** **13c**, **13d**, **13e**, **13f**) = 0.0262, 0.0125, 0.2612, 0.0311, 0.0153, 0.0613 for $logD_{7.4}$. Source data are provided as a Source Data file.

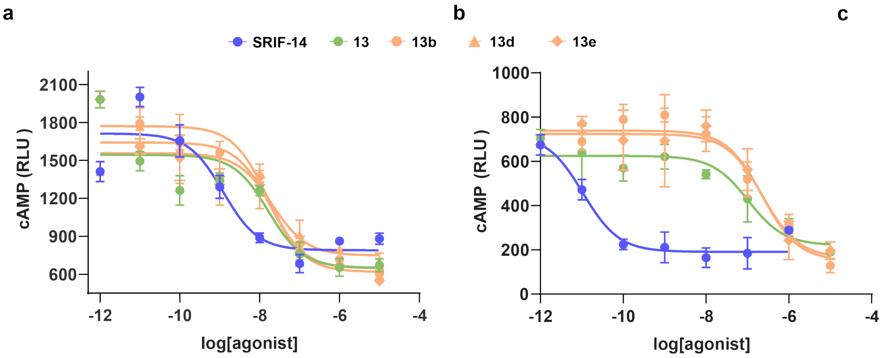

c

	IC$_{50}$ (nM)	
	sstr2 (A)	sstr5 (B)
SRIF-14	1.22 ± 0.65	0.01 ± 1.4
13	16.6 ± 4.2	105.2 ± 24.2
13a	> 10^4	> 10^4
13b	15.6 ± 4.3	228.3 ± 41.9
13d	20.3 ± 11.9	> 10^4
13e	19.2 ± 11.4	232.2 ± 70.4

Supplementary Figure 20. In vitro potency of 13 and its thio-analogs. Inhibition of forskolin-induced cAMP production by SRIF-14, **13**, **13b**, **13d**, and **13e** in HEK293T cells expressing SSTR2 (**a**) and SSTR5 (**b**). The IC_{50} values are mentioned in the table (**c**). *n* = 3 (biological triplicates) ± SEM. Source data are provided as a Source Data file.

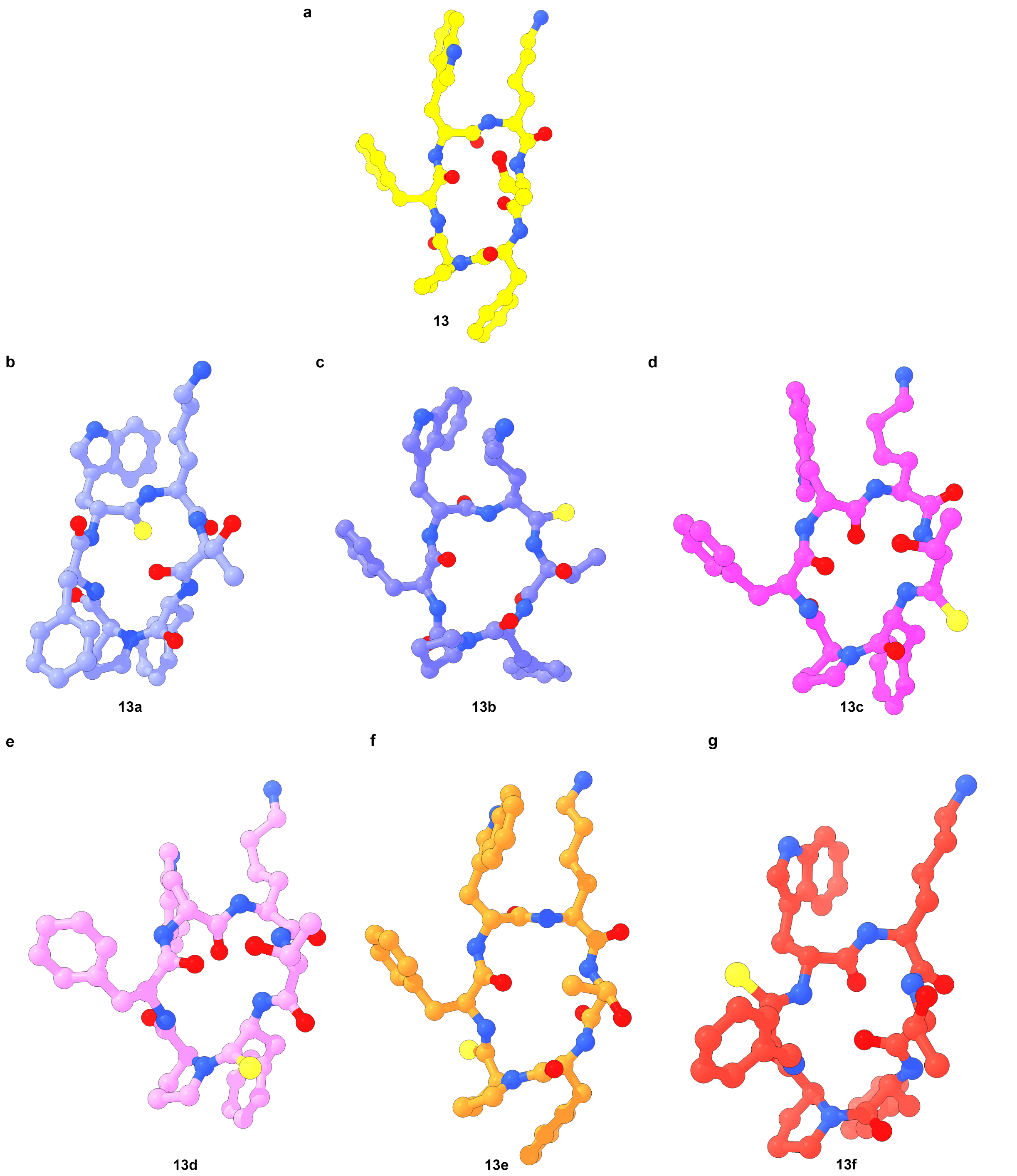

Supplementary Figure 21. NMR- deduced average structures of 13 and its thio-analogs. Average rMD structures of **13** (**a**, lemon), **13a** (**b**, orchid), **13b** (**c**, blue), **13c** (**d**, magenta). **13d** (**e**, lavender), **13e** (**f**, orange), and **13f** (**g**, red) in DMSO-d_6. The structures have been generated in ChimeraX.

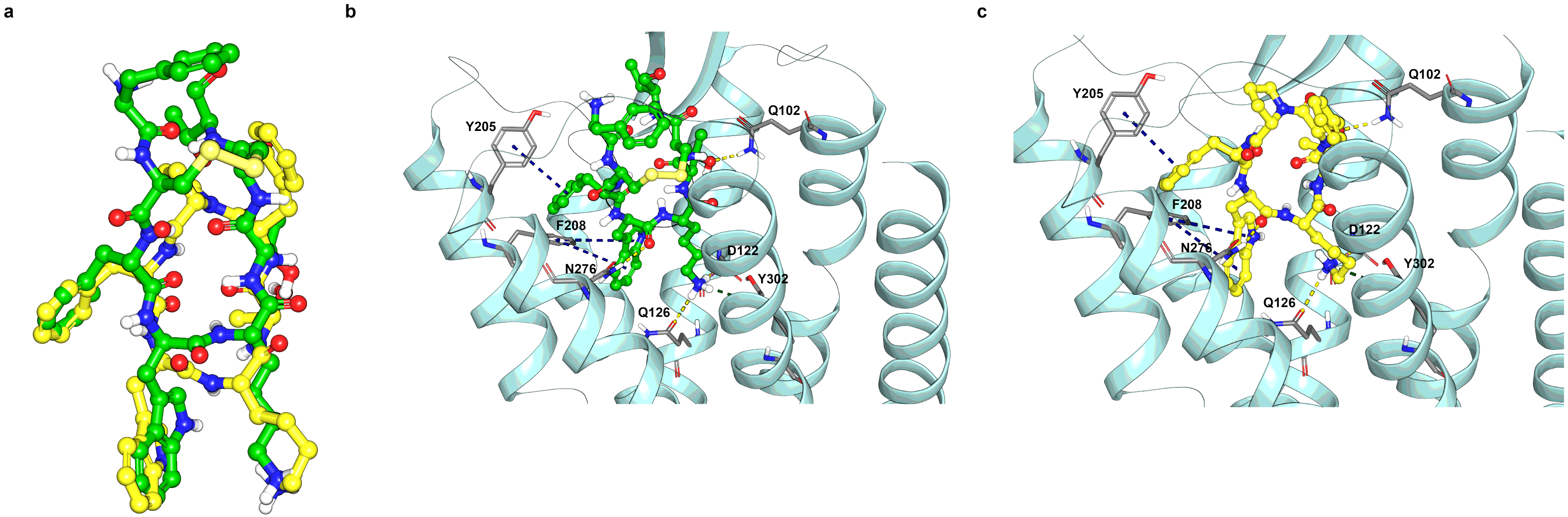

Supplementary Figure 22. Comparison of the SSTR2 bound structures of Octreotide and 13. a Superposition of the receptor bound structure of octreotide (green) and **13** (lemon) (minimum common structure RMSD 1.62 Å). Ligand binding pocket of SSTR2 (helices, cyan) with bound octreotide (**b**) and **13** (**c**). The key interacting partners in the binding pocket are highlighted in grey sticks. The dashed lines in the images indicate various non-covalent interactions between the peptides and SSTR2 (Yellow: Hydrogen bond; Magenta: Salt bridge; Blue: π-π stacking; Green: cation-π). The structures have been generated in Maestro GUI of Schrödinger Suite.

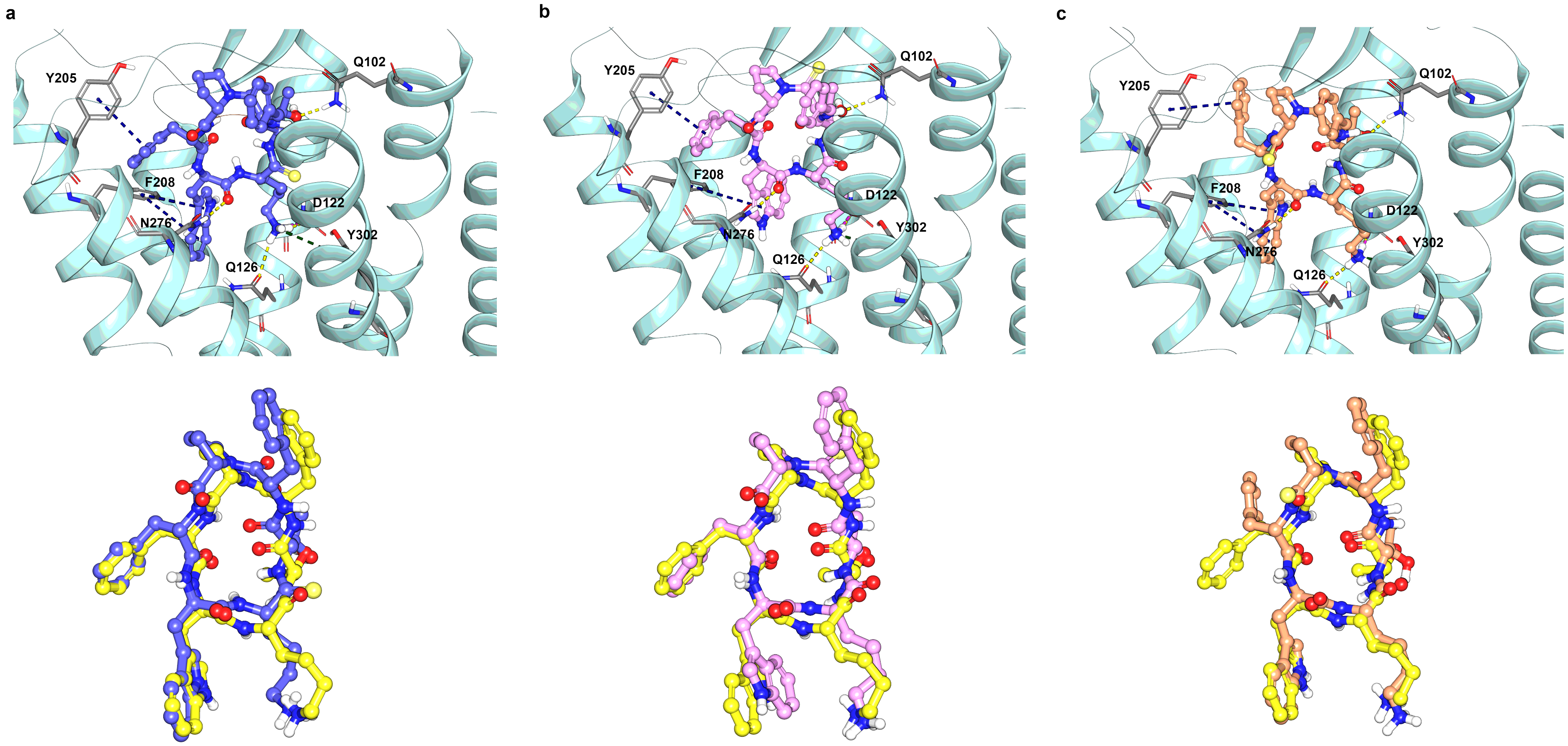

Supplementary Figure 23. Comparison of the SSTR2 bound structures of 13 and its active thio-analogs. (**a-c**, top panel) Ligand binding pocket of SSTR2 (helices, cyan) with bound **13b** (**a**), **13d** (**b**), **13e** (**c**). The key interacting partners in the binding pocket are highlighted in grey sticks. The dashed lines in the images indicate various non-covalent interactions between the peptides and SSTR2 (Yellow: Hydrogen bond; Magenta: Salt bridge; Blue: π-π stacking; Green: cation-π). (**a-c**, bottom panel) Superposition of the receptor bound structure of **13** (lemon) with the three bioactive analogs **13b** (blue), **13d** (lavender), and **13e** (orange), respectively. Maximum common structure RMSDs of **13b**, **13d**, and **13e** w.r.t. **13** are 1.55 Å, 1.83 Å, and 1.87 Å, respectively. The structures have been generated in Maestro GUI of Schrödinger Suite.

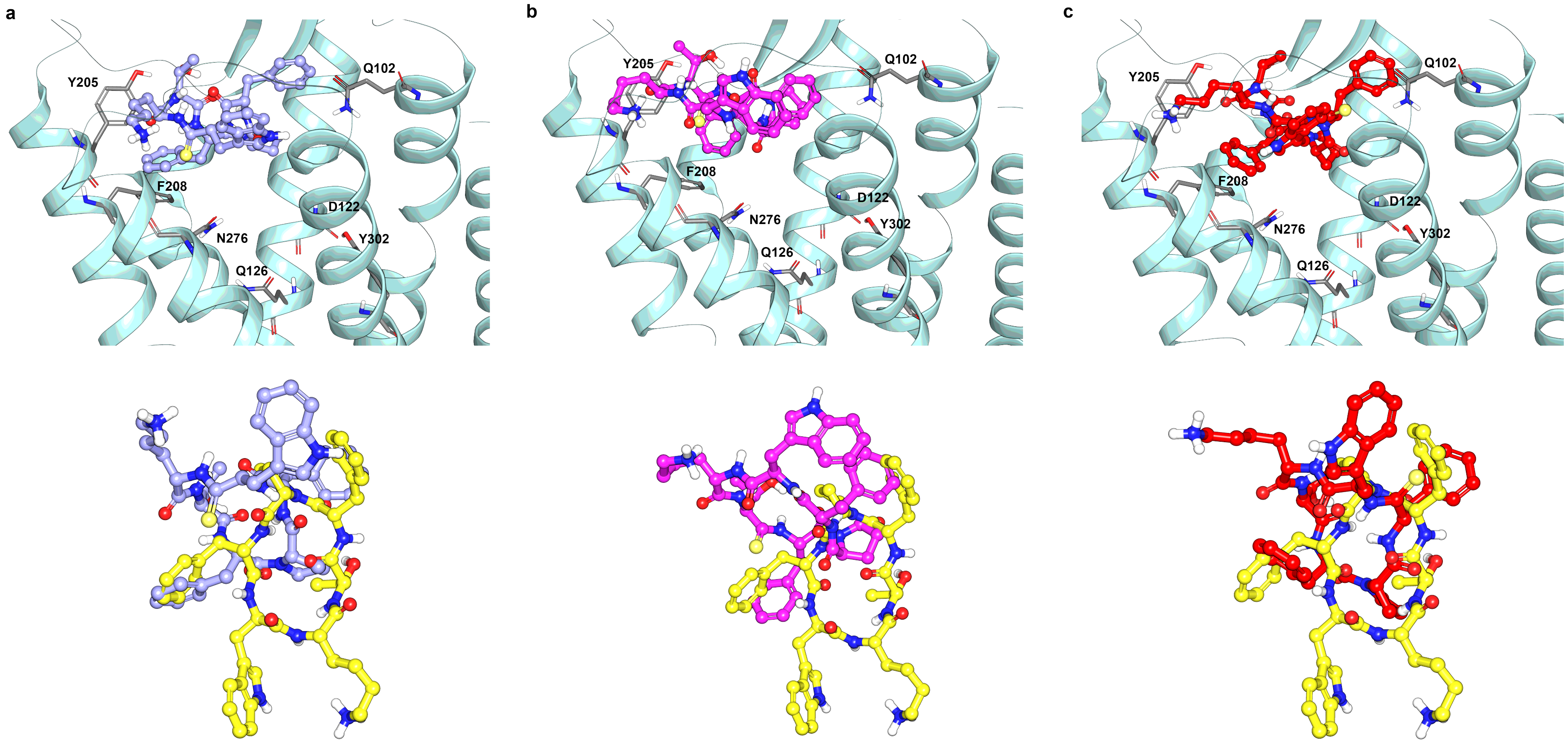

Supplementary Figure 24. Comparison of the SSTR2 bound structures of 13 and its inactive thio-analogs. (**a-c**, top panel) Ligand binding pocket of SSTR2 (helices, cyan) with bound **13a** (**a**), **13c** (**b**), **13f** (**c**). The key interacting partners in the binding pocket are highlighted in grey sticks. The dashed lines in the images indicate various non-covalent interactions between the peptides and SSTR2 (Yellow: Hydrogen bond; Magenta: Salt bridge; Blue: π-π stacking; Green: cation-π). (**a-c**, bottom panel) Superposition of the receptor bound structure of **13** (lemon) with the three inactive analogs **13a** (orchid), **13c** (magenta), and **13f** (red), respectively. Maximum common structure RMSDs of **13a**, **13c**, and **13f** w.r.t. **13** are 10.93 Å, 11.59 Å, and 10.62 Å, respectively. The structures have been generated in Maestro GUI of Schrödinger Suite.

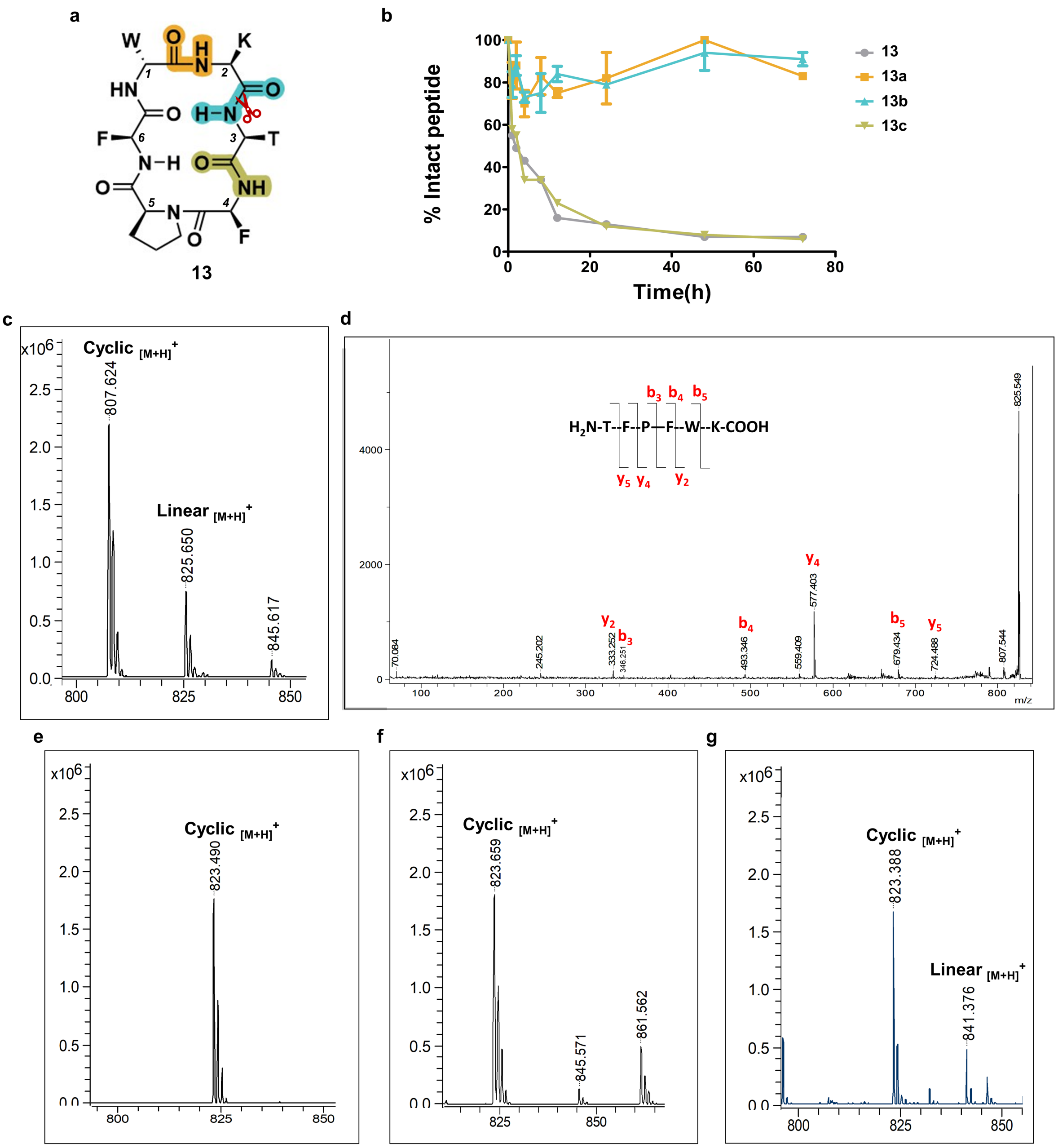

Supplementary Figure 25. Impact of O to S substitution on the protease cleavage site in macrocyclic peptides. **a** Chemical structure of **13**, with the site of cleavage (P1) between Lys^2 and Thr^3 highlighted. P1', P1, and P2 sites which were thioamidated are highlighted in olive (**13c**), teal (**13b**), and orange (**13a**) respectively. **b** Degradation kinetics of **13**, **13a**, **13b**, and **13c** in SIF over 72 hours. $n = 3 \pm$ SEM. Each point on the graph(s) represents mean values of three biological replicates. **c** MALDI profile of **13** after incubation in SIF for 1 hour, showed both cyclic and linear mass. **d** MS-MS of the linear peak revealed the Lys^2-Thr^3 amide bond to be the primary site of cleavage. MALDI profiles of **13a** (**e**), **13b** (**f**), and **13c** (**g**) after incubation in SIF for 1 hour clearly shows that thioamidation confers protection against the intestinal proteases in **13a** (P2) and **13b** (P1), but no such protection in **13c** (P1').

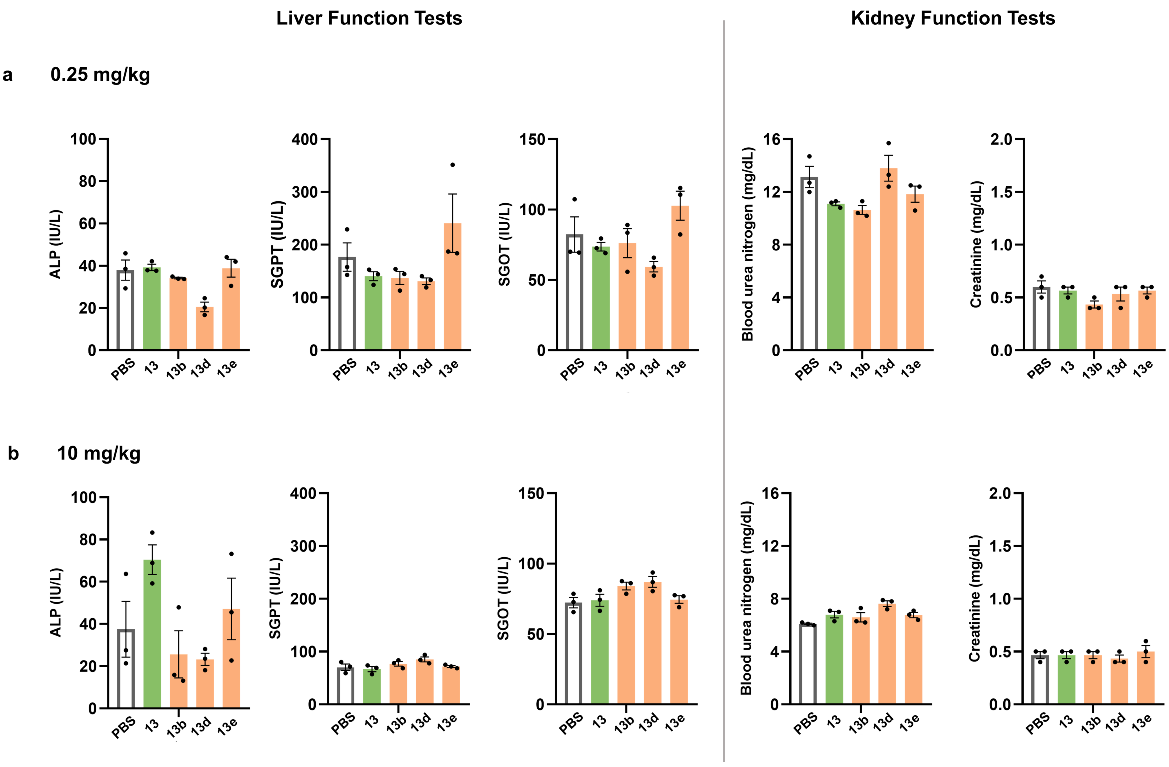

Supplementary Figure 26. In vivo acute toxicity analyses. Serum biochemistry analyses in male SD rats upon subcutaneous administration of **13**,**13b**, **13d**, and **13e** at 0.25 mg/kg (**a**) and 10mg/kg (**b**) to assess for acute toxicity. The reference range of the enzymes are ALP 44-147 IU/L, SGOT 74-143 IU/L, SGPT 63-175 IU/L, Urea 5-20 mg/dL, Creatinine 0.5-2.2 mg/dL $n = 3 \pm$ SEM. Each bar represents mean values of three biological replicates: dots are individual data points. Source data are provided as a Source Data file.

Supplementary Table 1. Calculated and observed m/z, HPLC retention time in C18 column, theoretical and observed lipophilicities for all the peptides assessed in this manuscript. **1-7b**: Solvent A: 0.1% TFA in water, solvent B: 0.1% TFA in acetonitrile gradient 10-60% solvent B over 20 minutes, **8-9f**: Solvent A: 0.1% TFA in water, solvent B: 0.1% TFA in acetonitrile gradient 10-50% solvent B over 30 minutes, **10-12h**, **PC**: Solvent A: 0.1% TFA in water, solvent B: 0.1% TFA in methanol gradient 80-100% solvent B over 20 minutes, **13-13f**: Solvent A: 0.1% TFA in water, solvent B: 0.1% TFA in acetonitrile gradient 30-80% solvent B over 20 minutes. The lower cases denote D-amino acid residues.

Peptides	Sequence	Calculated m/z $[M+H]^+$	Observed m/z $[M+H]^+$	AlogP	logD$_{7.4}$	t_R (min)
**1**	H_2N-F-K-COOH	294.2	294.2	-3.2	-2.9	5.3
**1a**	H_2N-F^{t}-K-COOH	310.2	310.2	-2.1	-2.1	11.2
**1b**	H_2N-F-^{NMe}K-COOH	308.2	308.2	-2.9	-2.0	6.8
**2**	H_2N-F-E-COOH	295.1	295.2	-2.4	-2.6	7.2
**2a**	H_2N-F^{t}-E-COOH	311.1	311.1	-1.3	-1.5	13.7
**2b**	H_2N-F-^{NMe}E-COOH	309.1	309.1	-2.2	-2.1	11.2
**3**	H_2N-F-G-COOH	223.1	223.0	-2.5	-2.2	7.9
**3a**	H_2N-F^{t}-G-COOH	239.1	239.1	-1.5	-1.6	15.1
**3b**	H_2N-F-^{NMe}G-COOH	237.1	236.9	-2.3	-1.7	11.7
**4**	H_2N-F-A-COOH	237.1	237.1	-2.1	-2.4	8.2
**4a**	H_2N-F^{t}-A-COOH	253.1	253.1	-1.0	-1.8	13.2
**4b**	H_2N-F-^{NMe}A-COOH	251.1	250.9	-1.9	-1.8	10.7
**5**	H_2N-F-V-COOH	265.2	265.1	-1.2	-1.3	12.4
**5a**	H_2N-F^{t}-V-COOH	281.1	281.1	-0.1	-0.9	16.7
**5b**	H_2N-F-^{NMe}V-COOH	279.1	279.0	-1.0	-0.6	16
**6**	H_2N-F-M-COOH	297.1	297.1	-1.7	-1.4	14.6
**6a**	H_2N-F^{t}-M-COOH	313.1	313.1	-0.6	-0.8	17.7
**6b**	H_2N-F-^{NMe}M-COOH	311.1	311.1	-1.5	-1.1	17.7
**7**	H_2N-F-W-COOH	352.2	352.1	-0.2	-0.6	19
**7a**	H_2N-F^{t}-W-COOH	368.1	368.2	0.9	0.1	21.4
**7b**	H_2N-F-^{NMe}W-COOH	366.1	366.1	-0.0	0.2	20.9
**8**	*cyclo*(a-A-A-A-A)	356.1	357.1	-2.3	-1.5	6.6
**8a**	*cyclo*(a^{t}-A-A-A-A)	372.2	372.2	-1.2	-0.3	11.6
**8b**	*cyclo*(a-A^{t}-A-A-A)	372.2	372.1	-1.2	-0.1	19.1
**8c**	*cyclo*(a-A-A^{t}-A-A)	372.2	372.2	-1.2	-0.2	18.7
**8d**	*cyclo*(a-A-A-A^{t}-A)	372.2	372.2	-1.2	-0.2	17.5
**8e**	*cyclo*(a-A-A-A-A^{t})	372.2	372.1	-1.2	-0.1	19.1
**9**	*cyclo*(a-A-A-A-A-A)	427.2	426.7	-2.7	-2.5	7.5
**9a**	*cyclo*(a^{t}-A-A-A-A-A)	443.2	442.9	-1.6	-0.7	18.3
**9b**	*cyclo*(a-A^{t}-A-A-A-A)	443.2	443.4	-1.6	-0.7	25.5
**9c**	*cyclo*(a-A-A^{t}-A-A-A)	443.2	442.9	-1.6	-0.6	21.8
**9d**	*cyclo*(a-A-A-A^{t}-A-A)	443.2	443.4	-1.6	-0.7	12.6
**9e**	*cyclo*(a-A-A-A-A^{t}-A)	443.2	443.4	-1.6	-0.5	27.9
**9f**	*cyclo*(a-A-A-A-A-A^{t})	443.2	443.4	-1.6	-0.2	31.4
**10**	*cyclo*(l-L-L-p-Y-L)	713.4	713.5	3.8	0.3	9.3
**10a**	*cyclo*(l^{t}-L-L-p-Y-L)	729.4	729.5	4.9	1.2	10.8
**10b**	*cyclo*(l-L^{t}- L-p-Y-L)	729.4	729.5	4.9	1.1	13.0
**10c**	*cyclo*(l-L-L^{t}- p-Y-L)	729.4	729.5	4.9	1.3	9.4
**10d**	*cyclo*(l-L-L-p^{t}-Y-L)	729.4	729.5	4.9	1.2	12.3
**10e**	*cyclo*(l-L-L-p-Y^{t}-L)	729.4	729.5	4.9	1.7	13.5
**10f**	*cyclo*(l-L-L-p-Y-L^{t})	729.4	751.4	4.9	1.5	14.0
**10g**	*cyclo*(^{NMe}l-L-L-p-Y-L)	726.9	727.2	4.0	1.0	10.5
**10h**	*cyclo*(l-^{NMe}L-L-p-Y-L)	726.9	727.3	4.0	1.5	13.7
**10i**	*cyclo*(l-L-^{NMe}L-p-Y-L)	726.9	727.2	4.0	1.1	11.4
**10j**	*cyclo*(l-L-L-p-^{NMe}Y-L)	726.9	727.3	4.0	1.0	10.5
**10k**	*cyclo*(l-L-L-p-Y-^{NMe}L)	726.9	727.2	4.0	1.2	11.2
**11**	*cyclo*(I-A-A-F-P-I-P)	710.4	710.3	1.6	0.1	5.3
**11a**	*cyclo*(I^{t}-A-A-F-P-I-P)	726.4	726.1	2.7	0.6	8.4
**11b**	*cyclo*(I-A^{t}-A-F-P-I-P)	726.4	726.3	2.7	0.3	6.9
**11c**	*cyclo*(I-A-A^{t}-F-P-I-P)	726.4	726.3	2.7	0.4	7.8

Peptides	Sequence	Calculated m/z [M+H]$^+$	Observed m/z [M+H]$^+$	AlogP	logD$_{7.4}$	t_R (min)
**11d**	*cyclo*(l-A-A-F^t-P-l-P)	726.4	726.3	2.7	0.3	9.8
**11e**	*cyclo*(l-A-A-F-P^t-l-P)	726.4	726.2	2.7	0.6	10.2
**11f**	*cyclo*(l-A-A-F-P-l^t-P)	726.4	726.2	2.7	0.4	9.1
**11g**	*cyclo*(l-A-A-F-P-l-P^t)	726.4	726.2	2.7	0.3	7.8
**12**	*cyclo*(l-L-p-l-L-a-P-L)	831.5	853.4*	3.1	0.8	12.6
**12a**	*cyclo*(l^t-L-p-l-L-a-P-L)	847.5	869.3*	4.1	1.3	14.5
**12b**	*cyclo*(l-L^t-p-l-L-a-P-L)	847.5	869.3*	4.1	1.6	15.5
**12c**	*cyclo*(l-L-p^t-l-L-a-P-L)	847.5	869.3*	4.1	2.3	19.8
**12d**	*cyclo*(l-L-p-l^t-L-a-P-L)	847.5	869.3*	4.1	1.8	17.1
**12e**	*cyclo*(l-L-p-l-L^t-a-P-L)	847.5	869.3*	4.1	1.4	15.0
**12f**	*cyclo*(l-L-p-l-L-a^t-P-L)	847.5	847.3	4.1	1.6	14.7
**12g**	*cyclo*(l-L-p-l-L-a-P^t-L)	847.5	847.4	4.1	1.6	17.1
**12h**	*cyclo*(l-L-p-l-L-a-P-L^t)	847.5	869.3*	4.1	1.5	16.4
**13**	*cyclo*(w-K-T-F-P-F)	807.4	807.5	1.0	-1.2	14.0
**13a**	*cyclo*(w^t-K-T-F-P-F)	823.4	823.5	2.0	-0.7	16.2
**13b**	*cyclo*(w-K^t-T-F-P-F)	823.4	823.4	2.0	-0.6	15.7
**13c**	*cyclo*(w-K-T^t-F-P-F)	823.4	823.4	2.0	-1.0	14.9
**13d**	*cyclo*(w-K-T-F^t-P-F)	823.4	823.4	2.0	-0.8	17.2
**13e**	*cyclo*(w-K-T-F-P^t-F)	823.4	823.4	2.0	-0.7	14.1
**13f**	*cyclo*(w-K-T-F-P-F^t)	823.4	823.4	2.0	-0.9	15.8
**PC**	*cyclo*(l-l-L-l-P-Y)	713.4	713.5	3.8	1.0	9.8

*[M+Na]$^+$

Supplementary Table 2. Fold change in permeability (P_e) of the thioamidated and N-methylated analogs of peptides **10**, **11**, **12** and **13** with respect to their parent scaffolds.

Peptide	Sequence	P_e (10^{-6} cm/s)	Fold Change
**10**	*cyclo*(l-L-L-p-Y-L)	2.5 ± 0.7	**-**
**10a**	*cyclo*(l^t-L-L-p-Y-L)	10.8 ± 1.6	**4.3**
**10b**	*cyclo*(l-L^t- L-p-Y-L)	4.3 ± 0.7	**1.7**
**10c**	*cyclo*(l-L-L^t- p-Y-L)	3.1 ± 0.3	**1.2**
**10d**	*cyclo*(l-L-L-p^t-Y-L)	4.2 ± 1.6	**1.7**
**10e**	*cyclo*(l-L-L-p-Y^t-L)	7.4 ± 0.6	**3**
**10f**	*cyclo*(l-L-L-p-Y-L^t)	7.3 ± 1.5	**2.9**
**10g**	*cyclo*(^{NMe}l-L-L-p-Y-L)	2.8 ± 0.2	**1.1**
**10h**	*cyclo*(l-^{NMe}L-L-p-Y-L)	9.1 ± 1.5	**3.6**
**10i**	*cyclo*(l-L-^{NMe}L-p-Y-L)	3.2 ± 0.1	**1.3**
**10j**	*cyclo*(l-L-L-p-^{NMe}Y-L)	4.6 ± 0.3	**1.8**
**10k**	*cyclo*(l-L-L-p-Y-^{NMe}L)	6.1 ± 0.3	**2.4**
**11**	*cyclo*(l-A-A-F-P-l-P)	0.4 ± 0.2	**-**
**11a**	*cyclo*(l^t-A-A-F-P-l-P)	4.7 ± 0.7	**11.8**
**11b**	*cyclo*(l-A^t-A-F-P-l-P)	0.4 ± 0.2	**1**
**11c**	*cyclo*(l-A-A^t-F-P-l-P)	1.0 ± 0.1	**2.5**
**11d**	*cyclo*(l-A-A-F^t-P-l-P)	0.4 ± 0.0	**1**
**11e**	*cyclo*(l-A-A-F-P^t-l-P)	0.8 ± 0.1	**2**
**11f**	*cyclo*(l-A-A-F-P-l^t-P)	1.1 ± 0.2	**2.8**
**11g**	*cyclo*(l-A-A-F-P-l-P^t)	0.4 ± 0.2	**1**
**12**	*cyclo*(l-L-p-l-L-a-P-L)	2.9 ± 0.6	**-**
**12a**	*cyclo*(l^t-L-p-l-L-a-P-L)	4.5 ± 0.7	**1.6**
**12b**	*cyclo*(l-L^t-p-l-L-a-P-L)	2.6 ± 0.6	**0.9**
**12c**	*cyclo*(l-L-p^t-l-L-a-P-L)	1.5 ± 0.3	**0.5**
**12d**	*cyclo*(l-L-p-l^t-L-a-P-L)	8.9 ± 0.9	**3.1**
**12e**	*cyclo*(l-L-p-l-L^t-a-P-L)	5.7 ± 0.6	**2**
**12f**	*cyclo*(l-L-p-l-L-a^t-P-L)	6.4 ± 1.0	**2.2**
**12g**	*cyclo*(l-L-p-l-L-a-P^t-L)	10.2 ± 2.5	**3.5**

Peptide	Sequence	P_e (10^{-6} cm/s)	Fold Change
**12h**	*cyclo*(l-L-p-l-L-a-P-L^{t})	7.7 ± 0.8	**2.7**
**13**	*cyclo*(w-K-T-F-P-F)	1.3 ± 0.5	**-**
**13a**	*cyclo*(w^{t}-K-T-F-P-F)	2.4 ± 0.2	**1.8**
**13b**	*cyclo*(w-K^{t}-T-F-P-F)	2.9 ± 0.3	**2.2**
**13c**	*cyclo*(w-K-T^{t}-F-P-F)	1.0 ± 0.2	**0.8**
**13d**	*cyclo*(w-K-T-F^{t}-P-F)	2.3 ± 0.1	**1.8**
**13e**	*cyclo*(w-K-T-F-P^{t}-F)	1.9 ± 0.7	**1.5**
**13f**	*cyclo*(w-K-T-F-P-F^{t})	1.5 ± 0.0	**1.1**

Supplementary Table 3. Average ϕ and φ values of each residue of **10**, **10a**, **10e**, and **10f**, from their fMD simulations measured in implicit solvent with dielectrics of DMSO (top), and $CDCl_3$ (bottom).

Residue	D-Leu1		Leu2		Leu3		D-Pro4		Tyr5		Leu6	
**DMSO (κ = 46.68)**												
	ϕ	φ	ϕ	φ	ϕ	φ	ϕ	φ	ϕ	φ	ϕ	φ
**10**	102.4	-29	-61.4	-54.5	-144.8	160.3	35.1	-102.7	-91.9	1.7	-104.9	-28.3
**10a**	116.4	24.1	-158.8	-57.2	-108.5	143.4	42.6	-120.3	-108.1	22.2	-79.9	-38.4
**10e**	79.5	-98.6	-70.1	-46.7	-109.8	130.4	69.7	-109.8	-122.3	6.0	-109.5	36.2
**10f**	96.3	-38.3	-72.5	-36.9	-147.1	94.6	62.0	-119.2	-49.4	-43.9	-74.5	-14.6
**$CDCl_3$ (κ = 4.81)**												
**10**	100.2	-74.9	-51.2	-66.0	-112.0	110.6	61.1	-123.6	-55.6	-29.3	-86.7	4.6
**10a**	121.7	-67.0	-82.9	-20.7	-125.6	101.9	39.5	-114.7	-62.7	-17.6	-85.1	-16.7
**10e**	75.6	-80.4	-61.1	-57.5	-116.6	162.3	35.2	-110.0	-123.9	22.1	-109.6	39.0
**10f**	110.6	36.4	-151.7	-77.8	-90.1	145.3	60.9	-119.7	-103.1	10.0	-93.1	-22.2

Supplementary Table 4. Caco-2 permeability assessment of **10** and its mono thioamidated analogs **10a-f** in presence and absence of P-gp efflux inhibitor Elacridar. *n* = 2. Since *n*<3, standard errors and associated statistics has not been deduced.

Compounds	Average Values						
	P_{app} (10^{-6} cm/sec)				Efflux Ratio	A to B % Recovery	B to A % Recovery
	AB	BA	AB+Elacridar	BA+Elacridar			
**10**	0.8	12.5	2.7	5.1	16.3	127	107
**10a**	3.3	75.4	13.0	16.9	23.2	92	110
**10c**	0	5.6	0	0.6	n.c.	94	84
**10e**	2.0	37.6	6.1	9.5	18.7	99	83
**10f**	3.0	41.8	6.8	8.9	13.9	74	74
**Propranolol**	16.6	34.1	n.c.	n.c.	2.1	102	120
**Atenolol**	0.4	1.4	n.c.	n.c.	3.5	82	124
**Digoxin**	0.7	29.5	1.5	6.2	40.9	121	121

n.c.: not calculated.

Supplementary Table 5. Single dose oral and intravenous pharmacokinetic parameters of **10**, **10a**, **10e**, and **10f** in male Wistar rats. *n* = 3 ± SEM. Each value represents mean of three biological replicates.

**per oral**						
**PK parameters**	**10**	**10a**	**10e**	**10f**	**10h**	**10k**
**$t_{1/2}$ (h)**	NC	3.8 ± 2.2	4.7 ± 0.6	1.9 ± 0.1	2.1 ± 0.4	16.7 ± 2.8
**t_{max} (h)**	0.1 ± 0.1	0.3 ± 0.0	0.8 ± 0.5	0.2 ± 0.1	1.0 ± 0.9	0.1 ± 0.1
**C_{max}(ng ml^{-1})**	3.5 ± 0.6	15.0 ± 0.7	47.2 ± 11.3	61.7 ± 16.1	13.1 ± 5.4	55.9 ± 4.0
**AUC_{last}(h ng ml^{-1})**	0.9 ± 0.3	26.9 ± 15.9	359.5 ± 58.1	120.8± 46.8	25.9 ± 15.0	344.9 ± 130.4
**MRT_{last} (h)**	0.2 ± 0.1	2.0 ± 1.3	5.9 ± 1.2	2.1 ± 0.4	1.3 ± 0.3	9.0 ± 1.3
**intravenous**						
**$t_{1/2}$ (h)**	2.6 ± 0.6	4.1 ± 0.9	1.6 ± 0.1	1.3 ± 0.1	0.5 ± 0.0	0.4 ± 0.1
**t_{max} (h)**	0.1 ± 0.0	0.2 ± 0.1	0.1 ± 0.0	0.1 ± 0.0	0.1 ± 0.0	0.1 ± 0.0
**C_{max}(ng ml^{-1})**	1046.7±187.1	48.1 ± 5.1	2660.9 ± 942.3	580.0 ± 58.9	434.6±73.2	1165.5±227.9
**C_0(ng ml^{-1})**	1730.4±437.2	50.4 ± 2.7	4461.4±2356.1	940.4±166.5	620.4 ± 87.0	1728.9±397.8
**AUC_{last}(h ng ml^{-1})**	548.1 ± 26.6	146.4 ± 23.2	2278.4 ± 330.7	389.7 ±12.9	212.2±38.6	509.5 ± 96.2
**Cl_obs (ml min^{-1} kg^{-1})**	28.4 ± 1.4	91.1 ± 22.6	7.5 ± 1.2	42.2 ±1.33	79.5 ± 14.5	33.3 ± 6.0
**MRT_{last} (h)**	1.6 ± 0.3	2.8 ± 0.0	1.5 ± 0.1	1.1 ± 0.121	0.4 ± 0.0	0.4 ± 0.0
**V_{ss_obs} (l kg^{-1})**	4.1 ± 0.9	28.6 ± 1.3	0.7 ± 0.1	3.2 ± 0.2	2.3 ± 0.5	0.8 ± 0.1

Supplementary Table 6. Caco-2 permeability assessment of **13** and its mono thioamidated analogs **13a-f.** *n* = 2. Since *n*<3, standard errors and associated statistics has not been deduced.

**Compound**	**Average Values**				
	**P_{app} (10^{-6} cm/s)**		**Efflux Ratio**	**A to B % Recovery**	**B to A % Recovery**
	**AB**	**BA**			
**13**	0	0	n.c.	60.3	61.9
**13a**	0	0	n.c.	46.5	50.1
**13b**	0	0	n.c.	50.5	44.7
**13c**	0	0	n.c.	56.5	53.9
**13d**	0	0	n.c.	67.2	62.4
**13e**	0	0	n.c.	64.9	64.8
**13f**	0	0	n.c.	13.7	13.7
**Propranolol**	22.8	25.4	1.1	71.0	90.7
**Atenolol**	0	0.3	n.c.	91.7	92.9

n.c.: not calculated.

Supplementary Table 7. Single dose subcutaneous pharmacokinetic parameters of **13**, **13a-f** in male Sprague-Dawley rats. *n* = 3 ± SEM. Each value represents mean of three biological replicates.

**PK Parameters**	**13**	**13a**	**13b**	**13d**	**13e**
**C_{max} (ng ml^{-1})**	205.2 ± 18.4	152.2 ± 27.4	98.3 ± 26.4	101.6 ± 9.5	59.1 ± 8.6
**AUC_{last} (ng h ml^{-1})**	220.8 ± 47.6	228.5 ± 23.9	154.2 ± 41.3	146.2 ± 24.3	121.5 ± 20.2
**$t_{1/2}$ (h)**	0.3 ± 0.05	0.7 ± 0.1	0.6 ± 0.2	1.1 ± 0.4	0.6 ± 0.3
**MRT_{last} (h)**	0.7 ± 0.05	1.1 ± 0.1	1.0 ± 0.04	1.1 ± 0.2	1.5 ± 0.1

Supplementary Materials and methods

Materials: All the Fmoc and orthogonally protected amino acids, O-(6-chlorobenzotriazol-1-yl)-N, N, N', N'-tetramethyluronium hexafluorophosphate (HCTU), 1-Hydroxy Benzotriazole (HOBt), 1-Hydroxy-7-azabenzotriazole (HOAt), 1-[Bis(dimethylamino)methylene]-1H-1,2,3-triazolo[4,5-b]pyridinium 3-oxide hexafluorophosphate (HATU), Rink amide AM and 2-Chlorotrityl chloride polystyrene resin were purchased from GL Biochem, Shanghai, China. *N,N'*-Diisopropylcarbodiimide (DIC), .*N,N*-diisopropylethylamine (DIPEA), Trifluoroacetic acid (TFA), Trifluoroethanol (TFE), Triisopropylsilane (TIPS), Triphenylphosphine, anhydrous Dichloromethane (DCM) anhydrous Tetrahydrofuran (THF), anhydrous Methanol, Diisopropylazodicarboxylate (DIAD), 1,8-Diazabicyclo[5.4.0]undec-7-ene (DBU), 2-Mercaptoethanol, Glacial acetic acid, N-methyl-2-pyrrolidone (NMP), 4-Nitro-o-phenylenediamine (DNB), Diphenylphosphoryl azide (DPPA), Sodium nitrite, Thionyl Chloride, Piperidine, Dimethyl sulpoxide-d_6, Chloroform-*d*, Methanol-d_4, Deuterium Oxide (D_2O), anhydrous Heptane, Ethylene glycol, 1-Octanol were all purchased from Sigma-Aldrich. All the reagents were used as obtained from the supplier. Solvents for RP-HPLC were purchased as HPLC grade and were used without further filtration or purification. All the reactions were performed in oven-dried glass apparatus. Reactions on solid support were carried out in plastic syringes (5 ml) fitted with a frit column plate (Torviq). Analytical thin-layer chromatography was performed on E-Merck silica gel 60 GF-254 (0.25 mm plates) pre-coated on aluminium sheet with various combinations of ethyl acetate and hexane as eluents. Reagents for the in vitro and in vivo assays: Pancreatin, Pepsin, Human liver microsome, NADPH, Protease activity kit and K_2-EDTA were also procured from Sigma-Aldrich. 1X Phosphate buffered saline, pH 7.4 was purchased from HiMedia Labs. Rapid Equilibrium Dialysis (RED) single-use plate with inserts was purchased from Thermo Fisher Scientific. Pre-coated PAMPA plates were purchased from Corning® Gentest. Rat growth hormone ELISA kit was purchased from Invitrogen.

Instrumentation: High-resolution mass spectra were recorded on a Bruker Daltonics ESI Q TOF-(Maxix Impact) with Nano LC (Proxeon easy nLC) mass spectrometer. ESI mass spectra were recorded in positive ion mode on a HCTultra ETD II ion trap spectrometer (PTM Discovery System, BrukerDaltonics, Germany). MALDI mass spectra were recorded on UltrafleXtreme TOF/TOF (BrukerDaltonics, Germany). Nuclear magnetic resonance (NMR) spectra were recorded at room temperature on Bruker AV 500 spectrometer (^{1}H, 500 MHz). All chemical shifts are reported in parts per million (ppm) from tetramethylsilane (TMS) (δ = 0) and were measured relative to the solvent in which the sample was analysed ($(CD_3)2SO$: δ 2.49 ppm, $CDCl_3$ δ 7.36 ppm for ^{1}H NMR). Analytical RP-HPLC was performed on a Shimadzu UFLC and iProminence system equipped with Prominence Diode Array (PDA) UV Detector at 210, 254 and 270 nm using an analytical column (Intek/Phenomenex/Kromasil C18, 250 mm x 4.6 mm I.D., 5 μm) at a flow rate of 1 mL/min and 4 mL/min. The mobile phase was composed of 0.1% TFA in H_2O and 0.1% TFA in acetonitrile (ACN)/methanol, passing through pumps A and B respectively. The gradients were adjusted based on the peptide polarity. The HPLC quantifications were done by monitoring the absorbance at 214 nm.

Peptide synthesis and purification

Loading of the first amino acid: Peptide synthesis was carried out using TCP-resin (1.33 mmol/g) following standard Fmoc strategy. Fmoc-Amino acid-OH (1.25 eq.) was attached to the TCP resin with DIPEA (2.5 eq.) in anhydrous DCM (2 mL) at room temperature for 6-8 h. After loading the first amino acid, the remaining trityl chloride groups were capped by a solution of DCM, MeOH, DIPEA for 20 min. The resin was washed thoroughly with DCM (2x), DMF (3x) and MeOH (5x). The loading capacity of the resin was estimated from its dry weight and ranged from 0.6-0.8 mmol/g. After incorporation of the first amino acid, the polymer resin was washed and later swollen in DMF.

Fmoc deprotection and subsequent coupling: The resin-bound Fmoc peptide was treated with 20% piperidine in DMF (v/v) for 5 minutes and a second time for 15 minutes (10% piperidine in DMF (v/v) for 1 min twice, for thio-peptides). The resin was washed with DMF (5x) thoroughly by vigorous shaking to remove traces of piperidine. Subsequently, N_α protected amino acid was coupled to the amino group of the polymer bound substrate using HOBt/DIC (2.5 eq.) coupling in DMF for 3 h and henceforth the peptide chain was elongated.

Cleavage from resin and head-to-tail cyclization: All the peptides were cleaved off from the resin with a mild treatment of acetic acid/2,2,2-trifluoroethanol (TFE) solution in dichloromethane (DCM) (3:1:6), which was repeated 3 times with an hour each.

The head-to-tail cyclization was performed with diphenylphosphorylacid azide (DPPA) (3eq), applying the solid base method using $NaHCO_3$ (5 eq) in DMF at a concentration of 0.1 mM [1]. After the completion of cyclization, which was monitored by HPLC and ESI mass spectroscopy, DMF was removed using rotary evaporator. To remove excess DPPA, a work-up was done using saturated $NaHCO_3$ and DCM (1:1). The DCM layer was collected and evaporated to dryness. In case of peptides **13-13f**, the reaction solution (in DMF) was concentrated and added to chilled brine solution for precipitation. The supernatant containing DMF and excess DPPA was then removed. Before purification, the protecting groups were removed in solution by treating the peptides with 50% TFA in DCM and 2% TIPS for 20 minutes. The pure peptides were obtained by reversed phase high-performance liquid chromatography (RP-HPLC).

Synthesis of thioamide building blocks and incorporation onto the solid support: Thioamidated peptides were synthesized following a protocol optimized in the laboratory (Khatri, Bhat & Chatterjee, 2020), which has been reported earlier [2].

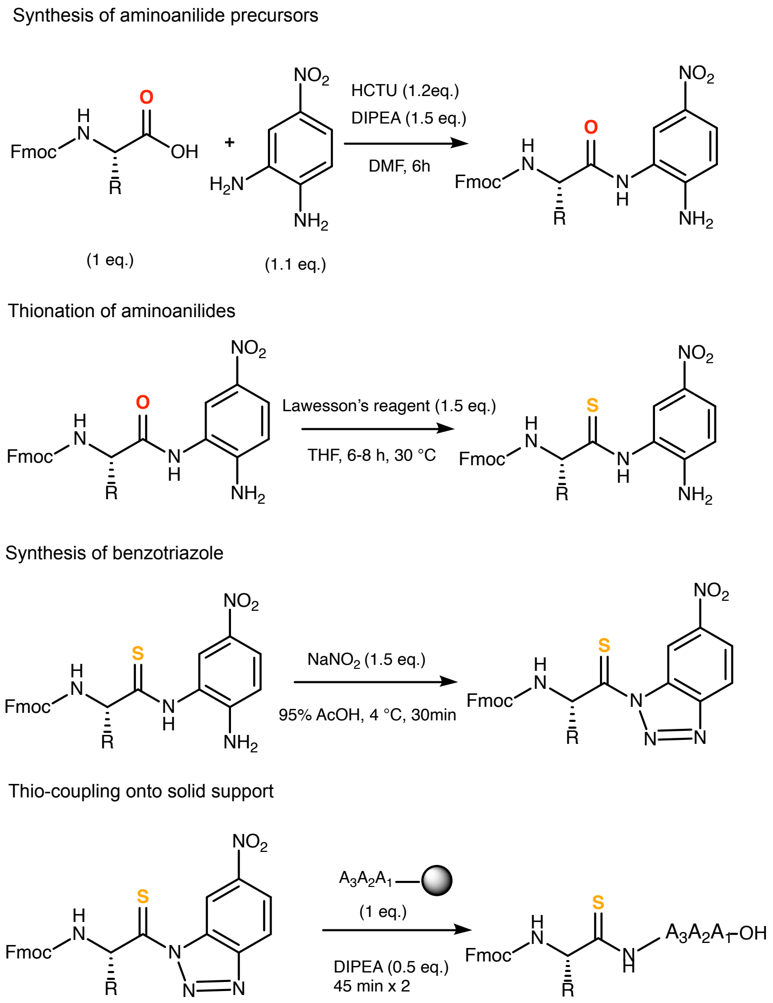

Supplementary Figure 27. Synthetic scheme for thio-amino acids incorporation. Synthesis of thioamidated building blocks and coupling onto solid support.

Synthesis of aminoanilide precursors: A 0.1-0.3 M solution of Fmoc-Aminoacid-OH (1 eq.) was prepared in DMF. HCTU (1.2 eq.) and DIPEA (1.5 eq.) was added to the solution, followed by the addition of 4-Nitro-o-phenylenediamine (1.1 eq.). the reaction was stirred vigorously at room temperature for 6h and the progress was monitored by TLC. The reaction mixture was poured into chilled water and filtered using a sintered disc filter-funnel. The yellow-coloured solid obtained after filtration was dissolved in ethyl acetate and washed with 1N HCl (2x) and saturated $NaHCO_3$ (2x). The organic fractions were combined, dried over Na_2SO_4, and evaporated to dryness, yielding respective aminoanilides.

General procedure for thionating aminoanilide precursors: To a 0.1-0.3 M solution of aminoanilide (1eq.) in anhydrous THF, 1.5 equivalence of Lawesson's reagent was added. The reaction was stirred at 30°C for 6-8 h. The progress of the reaction was monitored by TLC. After completion, the reaction mixture was filtered through a sintered disc filter-funnel, evaporated to dryness, and dissolved

in EtOAc. The solution was washed with saturated $NaHCO_3$ (2x), dried over Na_2SO_4, and evaporated to dryness, yielding the respective thioaminoanilides as a yellow-coloured solid.

General procedure for the synthesis of benzotriazole: To a 0.1-0.3 M solution of thioaminoanilide in glacial AcOH containing 5 % water, 1.5 equivalence of sodium nitrite was added at 4°C. The reaction mixture was stirred at 4 °C for 45 min. The progress of the reaction mixture was monitored by TLC. After completion, the reaction mixture was poured into chilled water and centrifuged. The supernatant was discarded, and the light orange precipitate was dissolved in DCM. The DCM solution was washed with saturated $NaHCO_3$ (2x), brine solution (1x), and dried over Na_2SO_4. This solution was directly utilized for incorporating the thiobenzotriazolides onto the solid support.

General procedure for the incorporation of thio-benzotriazole onto a growing peptide chain: The Fmoc deprotected peptide was swollen in DCM in a fritted syringe prior to the coupling. The solution of thiobenzotriazolide precursor (2 eq. with respect to the resin loading) in DCM was taken into the syringe along with 0.5 equivalence of DIPEA. The syringe was protected from light exposure and rotated in a mechanical rotor for 45 min. This step was repeated one more time. Finally, the resin was washed with DCM (5x).

On resin N-methylation of peptides: A modified protocol for Fukuyama & Mitsunobu reaction on the solid support was utilized for selective N-methylation of amino acid residue [1]. The first step involves amine activation by o-nitrobenzenesulfonyl group (o-NBS), followed by the alkylation of the activated amine by triphenylphosphine (PPh_3), diisopropylazodicarboxylate (DIAD), and anhydrous MeOH in THF. Finally, the o-NBS group is removed using DBU and β-mercaptoethanol, yielding the N-methylated amino acid bound to the solid support. This method was found to be compatible with different functional group containing amino acids and could be also performed on a growing peptide chain to selectively N-methylate the N-terminal residue. Coupling of the next amino acid onto the hindered N-methylated amino group on the resin requires strong coupling reagents like HoAT/HATU. This method was found to be rapid and highly efficient in synthesizing multiple N-methylated peptides with different functional groups in modest yields.

Reversed phase purification of peptides: For peptides **1-7**, **8-9f**, and **13-13f**, binary gradients of 10% B to 60% B, 10% B to 50% B, and 30% B to 80% B were respectively used, where solvent A was 0.1% TFA in H_2O and B was 0.1% TFA in ACN. For peptides **10-10f**, **11-11g** and **12-12h** a suitably adjusted gradient of 80% B to 100% B was used for purification, where solvent A was 0.1% TFA in H_2O and B was 0.1% TFA in methanol. For purification the flow rate was 4 mL/min.

NMR data acquisition

All the acquisitions were performed at 25°C. The concentration of all the peptides were ~1 mM. Spectra of peptides **10-10f** were acquired in both DMSO-d_6. and $CDCl_3$. Spectra of peptides **13-13f** were acquired in DMSO-d_6. Standard Bruker pulse sequences zgesgp for 1-D, mlevesgpph (60 ms mixing time) for TOCSY and roesyesgpph (200 ms mixing time) for ROESY were used with 2048 data

points in direct dimension and 512 data points in indirect dimension to acquire the NMR data. Data processing was done using iNMR software (version 6.3.3), and the 2D NMR data were analyzed with SPARKY (Goddard & Kneller, 2004) [3]. The chemical shift tables were generated from TOCSY and ^{1}H spectra. The sequential assignments and inter- and intra-residue NOEs were determined through ROESY. The NOEs were then integrated, and the integration values were converted to distances using the formula $V=Kd^{-6}$, where V is the integrated peak volume, K is a constant (determined using resolved Hβ1-Hβ2 distance of Tyr or Hβ-Hγ distance of Thr) and d is the distance between the protons.

Hydrogen-deuterium exchange was performed by preparing a solution of the peptides in 20% D_2O, 80% DMSO-d_6 and 20% CD_3OD, 80% $CDCl_3$. ^{1}H spectra were acquired for the next 24 h at the following time points – 0, 10, 20, 30, 40, 50, 60, 120, 240, 480, 720, 1440 mins.

Structure calculation

To calculate the solution structure of the peptides, we have used charmM force field (Brooks et al., 1983) via the interface of BIOVIA Discovery Studio Suite (v18.1.0.17334) [4]. The distance restraints obtained from 2D NMR, were converted into a charm restraint file using an inhouse Perl script. The resulting file was then used to define NOE restraints inside the charmM syntax. To the distance, 10% were added or subtracted to define the upper and lower limits respectively. If there were any methyl protons involved in the restraints, an additional 0.4 Å per methyl group (pseudoatom correction) were added to the upper limit to compensate for the errors involved. Dielectric constants of DMSO (46.68) and Chloroform (4.81) were applied in the implicit solvent system before running the simulations. The initial structure was obtained by following a simulated annealing protocol. It was then refined by distance and dihedral angle constraints derived from ^{1}H NMR spectra and Bystrov equation respectively followed by a 200 ns restrained molecular dynamics run. The average over the dynamics run was considered to be the final average structure for **13**, **13a-f**. For **10**, **10a**, **10e**, and **10f** the average structures from the restraint MD simulation (rMD) were further simulated for 200ns without any distance and angular constraints (free MD simulation or fMD). Average structures from these two simulations have been used for further analyses.

Docking studies

The coordinates of the SSTR2 in complex with octreotide was fetched from the PDB entry 7T11. The structure of SSTR2 was prepared at pH 7.4 using Protein Preparation Wizard in Schrödinger Suite (Schrödinger Release 2020-2) [5]. The coordinates of the average structure of the peptide ligands (**13**, **13a-f**) as obtained from NMR were used for the docking studies. The receptor grid suitable for docking peptides was generated with centroid of bound octreotide in 7T11 as centre of the receptor grid box. Redocking of octreotide was performed to validate the docking protocol. The peptides (octreotide, **13**, and **13a-f**) were docked into the SSTR2 binding site using the Standard Precision (SP)-peptide and flexible ligand sampling settings available in the Glide module of Schrödinger suite. The docked poses of the peptides and the interactions between the peptides and SSTR2 were visualized in Maestro GUI (version 12.4.072) of Schrödinger suite. OPLS3e force field was used for the computational studies [6]. The images of all the receptor-ligand interactions presented in this manuscript are generated using

Maestro GUI. In the images, unless otherwise mentioned, the heteroatoms are coloured as per usual computational chemistry convention (Red: Oxygen; Blue: Nitrogen; Yellow: Sulphur). The dashed lines in the images (Fig. S19-S21) indicate various non-covalent interactions between the peptide ligands and SSTR2 (Yellow: Hydrogen bond; Magenta: Salt bridge; Blue: π-π stacking; Green: cation-π).

HPLC retention time analysis

All dipeptides and cyclic peptides were run on their respective binary gradients (mentioned previously in Page: S30) of 20 mins in HPLC on a C18 (Phenomenex, 100 mm x 4.6 mm) column at 1 mL/min to assess relative hydrophobicity.

AlogP calculation

All AlogP calculations were done under molecular descriptor window in BIOVIA Discovery Studio Suite (v18.1.0.17334).

Octanol-Buffer partition coefficient ($logD_{7.4}$)

Shake flask method was used to determine the octanol water partition coefficient. Octanol and buffer were kept for pre-saturation overnight before the experiment. 100µM of compound was vigorously shaken in the equal volume (0.5 ml each) of buffer and octanol mixture for 2 h. After completion the mixture was allowed to settle, layers were separated and quantified using UV-Vis spectroscopy.

Heptane-ethylene glycol partition coefficient

Shake flask method was used to determine the heptane-ethylene glycol partition coefficient. Pre-saturation is not required in this case, 100µM of compound was vigorously shaken in the equal volume (0.5 ml each) of heptane and ethylene glycol mixture for 2 h. After completion the mixture was allowed to settle, layers were separated and quantified using UV-Vis spectroscopy.

Solubility measurements

Solubility measurement of peptides **10** and its thio analogs **10a-10f** were performed following previously reported protocol by Over & Kilhberg [7]. Peptide concentration was 100µM.

Parallel Artificial Membrane Permeability Assay (PAMPA)

96-well BD Corning® pre-coated PAMPA plates were used following the supplier's protocol. Donor wells were prepared by adding 300 µl of 100 µM of peptide in 5% DMSO/PBS at pH 7.4. The acceptor well was prepared by adding 200 µl of 5% DMSO/PBS at pH 7.4. The acceptor plate was gently placed on top of donor plate. This system was left undisturbed for 4 h at room temperature. After incubation, the peptide concentration was determined using UV/Vis spectroscopy & LCMS. The permeability was calculated using following formula.

$$P_e = \frac{-\ln[1-C_A/C_{equilibrium}]}{S^*(1/V_D+1/V_A)^*t}$$

where,

V_D = Donor well volume (0.3 ml)

V_A = Acceptor well volume (0.2 ml)

S = Membrane area (0.3 cm^2)

t = Incubation time in sec

C_A = Concentration of compound in acceptor well

$C_{equilibrium}$ = Equilibrium concentration (compound concentration across donor and acceptor well if the membrane is 100% permeable to compound)

$$C_{equilibrium} = \frac{[C_D^*V_D - C_A^*V_A]}{(V_D - V_A)}$$

Permeability across Caco-2 cell line

Following stock solutions were prepared: Peptides were dissolved in DMSO to prepare a stock solution of 2 mM which was further diluted to 1 mM as the working stock. The final peptide concentration in the assay condition was 10 µM. Elacridar (inhibitor of P-gp transporter) was dissolved in DMSO to prepare a stock of 10 mM concentration (working stock: 50 µM). A 10 mM stock of Digoxin (substrate of P-gp transporter) was prepared in DMSO ((working stock: 10 µM). Fetal bovine serum, L-glutamine, MEM non-essential amino acids, antibiotic antimycotic solutions, amphotericin B was added to DMEM to prepare the culture medium. HBSS-HEPES buffer (pH 7.4) was prepared.

Method: Prior to the start of the assay, the cell monolayer was washed and pre-incubated at 37 ºC and 5% CO_2 for 30 min with HBSS-HEPES buffer containing 1% DMSO. TEER values were measured before starting and after completion of the assay.

For the apical to basolateral (A to B) assay, 0.5 mL buffer solution containing one of the above combinations was added to the apical compartment and 1.5 mL buffer containing 1% DMSO was added to the basolateral compartment. The assay plate was incubated at 37 ºC and 5% CO_2. 100 µL samples from apical side at 0 and 120 min and 150 µL samples from the basolateral compartment at 30, 60, 90 and 120 min were withdrawn into acetonitrile containing 96-well plate.

Similarly, for the basolateral to apical (B to A) assay, 0.5 mL buffer containing 1% DMSO and 1.5 mL buffer solution containing one of the above combinations were added to the apical

compartment and basolateral compartment respectively. The assay plate was incubated at 37 ºC and 5% CO_2. 150 µL samples from basolateral side at 0 and 120 min and 100 µL samples from the apical compartment at 30, 60, 90 and 120 min were withdrawn into a 96-well plate containing acetonitrile.

At the end of the assay, the cell monolayer was washed with 500 µL buffer containing 1% DMSO and the washings were collected. Finally, the monolayer was washed with 500 µL acetonitrile to lyse the cells and the lysate was collected in tubes.

Analysis: A fit-for-purpose LC-MS/MS method was used for the estimation of peptide(s) as well as the positive controls. Cumulative amount of peptides and Digoxin (Q) transported at teach time point was plotted as a function of time. The slope was used to calculate the rate of appearance of peptides and Digoxin in the basolateral compartment.

Apparent permeability (P_{app}) was calculated using the following formula:

$P_{app} = (dQ/dt)/(A*C_0)$

where, dQ/dt is the rate of appearance of the peptides and digoxin in the receiver compartment.

A is the surface area of the membrane (1.12 cm^2).

C_0 is the initial concentration of the test items and Digoxin.

The efflux ratios were compared with and without Elacridar to assess whether the peptides are substrates of efflux transporter.

In vitro stability assessment of the peptides

Stability in Simulated Gastric Fluid (SGF)

SGF was prepared according to USP specifications (Test Solutions, United States Pharmacopeia 35, NF 30, 2012) [(8)]. Sodium chloride (0.2 g) was added to a 100 mL flask and dissolved in 50 mL of water. Then 0.7 mL of 10M HCl was added to adjust the pH of the solution to 1.2. To this, 0.32 g of Pepsin was added and dissolved with gentle shaking and the volume made up to 100 mL with water. Pepsin was added only after the pH was adjusted to 1.2. The protease activity of SGF solution ranged from 11,000-15,000 units/mL. 100 µM of peptide was incubated with the SGF solution and 50 µL was aliquoted at various time point. To quench the reaction, thrice the volume of chilled MeOH was added. Quantification of intact peptides was done using HPLC.

Stability in Simulated Intestinal Fluid (SIF)

SIF was prepared according to USP specifications (Test Solutions, United States Pharmacopeia 35, NF 30, 2012) [8]. Monobasic potassium phosphate (0.68 g) was dissolved in 25 mL of water, then 7.7 mL of 0.2 N NaOH was added to adjust the pH to 6.8. To this, 1 g of pancreatin was added and shaken gently until dissolved and the volume adjusted to 100 mL with water. Pancreatin was added after adjusting the pH of the solution to 6.8 to avoid precipitation of the enzyme. The protease activity of SIF solution ranged from 41,000-46,000 units/mL. 100 µM of peptide was incubated with the SIF solution

and 50 µL was aliquoted at various time points. To quench the reaction, equal volume of 0.1N HCl was added. Quantification of intact peptides was done using HPLC.

Stability in Human Liver Microsome

Microsomal stability was determined upon incubation of the peptides (100 µM) with human liver microsomes (conc. of cytochrome P450 = 0.25 µM) in the presence of 100 mM potassium phosphate buffer (pH 7.4) at 37˚C. The reaction mixture was prewarmed at 37˚C for 2 min before addition of NADPH (1.2 mM) cofactor. Aliquots of the reaction mixture at 0, 5, 15, and 30 min were added to chilled acetonitrile (thrice the volume of aliquoted mixture), and the samples were centrifuged at 2500 *g* for 5 min prior analysis for half-life determination. For control experiments, NADPH and/or liver microsomes were omitted from these incubations. In vitro liver microsomal half-lives ($t_{1/2}$) were scaled to hepatic intrinsic clearance (CL_{int}), as described by Houston et al. [9].

Ex vivo stability in Human Plasma

Blood was freshly drawn from a healthy adult and plasma was separated from whole blood by centrifugation for 10 min at 1000-2000 g in a refrigerated centrifuge. Required volume of plasma was pre-incubated at 37˚C prior to the experiment for 2 min. Plasma was spiked with the peptides (100 µM) and at different time points plasma (50 µL) was aliquoted. The reaction was quenched through heat inactivation for 2 min. Samples were centrifuged at 10,000 *g* for 10 minutes to allow all the proteins to settle down. The supernatant was separated and used for HPLC quantification.

Rapid Equilibrium Dialysis (RED) Assay

Plasma was extracted as mentioned in the previous section. Plasma was spiked with 100 µM peptide sample, volume was adjusted to 500 µL. The spiked plasma was added to the sample chamber, indicated by the red coloured ring. In the other chamber, 750 µL (as mentioned in the Thermo Fischer Scientific protocol) dialysis buffer (pH 7.4) containing 100 mM sodium phosphate, 150 mM sodium chloride was added. This entire unit was covered with a sealing tape and incubated at 37˚C for 4 h under mild shaking condition (300 rpm). After 4h, the unit was taken out, the seal was removed, and volume loss was checked (if any). Equal volumes (250 µL) of sample and buffer was aliquoted from both the chambers. To make the conditions and reaction constituents uniform, a certain volume of dialysis buffer was added to the aliquoted spiked plasma and the same volume of fresh plasma was added to the aliquoted sample from the buffer chamber. Chilled ACN was added to both to precipitate proteins and release peptides. Samples were centrifuged for 10 mins at 13,000 *g*, the supernatant was separated and used for HPLC quantification. The test compound concentration was measured in both buffer and plasma chamber through relative peak area analysis. Percentage of plasma bound peptide was calculated using the following formula:

% Free = (Peptide conc. in buffer chamber/Peptide conc. in plasma chamber) x 100

% Bound = 100 - % Free

GloSensor assay for the in vitro activity of Somatostatin analogs

HEK293T cells were maintained using high glucose (4500 mg/litre) DMEM supplemented with 10% FBS at 37°C with 5% CO_2 in a humidified incubator. Briefly, HEK-293T cells were co-transfected with human somatostatin receptor plasmid cDNA and cAMP biosensor (pGloSensorTM-22F plasmid; Promega Corp.) using PEImax in the ratio of 1 (DNA): 4 (PEImax). The transfected cells were incubated at 37°C in tissue culture incubator with 5% CO_2 for 14-16 hours. For GloSensor cAMP assay, after overnight incubation, media from the plate was aspirated and 100µl of sodium luciferin solution (1 mg/ml) in drug buffer (1X HBSS (pH7.4) and 20mM HEPES) was added to the cell plates. The plates were then incubated at 37°C in tissue culture incubator with 5% CO_2 for 60 minutes. For, GloSensor, compounds (**13, 13a-13f**) were diluted from stock solution (1mM in Nuclease free water) in drug buffer. Cells were treated with 20 µL of 6X diluted standard SRIF-14 (10000 nM to 0.01 nM) or with test compounds (in triplicates) and were incubated in a humidified tissue culture incubator at 37°C with 5% CO_2 for 10-15 minutes to get a steady state condition. Ligand incubation was followed by addition of 20 µL of 7X (70µM) forskolin (FSK) to the plate. FSK incubation was followed by incubation at 37°C for 10-15 minutes and measurement of luminescence per well using luminescence plate reader (BMG Labtech). The results were plotted as inhibition of FSK stimulated cAMP response (relative luminescence units) using nonlinear regression analysis by Graph-Pad Prism 8.

In vivo pharmacokinetics in rats

For oral bioavailability study of peptides: Animal care and *in vivo* procedures were conducted according to guidelines and protocols from the IEAC and CPCSEA. Jugular vein-cannulated male Wistar rats (230–250 g) were used for these studies. All animals were housed individually in the animal facility at Anthem Biosciences Pvt. Ltd and were acclimatized for 3 days. All animals were fasted overnight before dosing, whereas access to water was provided ad libitum. The peptides were administered orally (p.o.) and intravenously (i.v.). Fasted state animals were administered with peptides in a recommended vehicle 2% v/v DMSO + 98% v/v olive oil by oral gavage route with a dose of 10 mg/kg body weight at dose volume of 10 mL/kg body weight (b.w.). For i.v., the dosage was 1 mg/kg b.w. Blood specimens were collected at time points: 0.08, 0.25. 0.5. 1, 2, 3, 4, 6, 8, 24 h from jugular vein using syringes into pre-labelled tubes containing anticoagulant (K_2EDTA-2 mg/mL blood). Collected blood specimens were centrifuged at 2000 *g*, 4ºC for 10 minutes and plasma was separated and stored at -80ºC until analysis.

For subcutaneous (s.c.) administration of peptides: Single dose subcutaneous PK studies were performed in male Sprague – Dawley rats (230-250 g). Similar procedure for acclimatization was followed in this case as well. Peptides were administered in cell culture grade PBS at 250 µg/kg b.w. dosage and blood samples were collected at time points: 0, 0.25. 0.5. 1, 2, 3, 4, 6, 8, 24, 48 h. Plasma processing was done as mentioned previously.

Analysis was done through LC/MS/MS. In most of the cases the LLOQ was 1-2 ng/mL. The pharmacokinetic parameters such as C_{max}, T_{max}, $AUC_{0\text{-}t}$, $AUC_{0\text{-}inf}$, $T_{1/2,}$ MRT_{last}, Vss, and CL_{int} of the

analyte in rat plasma were be determined from the concentration time data by using non-compartmental analysis (Phoenix WinNonlin 6.3, Pharsight Corporation, USA).

In vivo inhibition of growth hormone secretion in rats

Animal care and in vivo procedures were conducted according to guidelines and protocols from the IEAC and CPCSEA. Animals were housed individually in Central Animal Facility, IISc. After procuring, the animals were kept for acclimatization for 3-7 days in LD cycle and temperature-controlled rooms. Access to food and water was provided ad libitum. Male Sprague-Dawley rats (230-250 g) were subcutaneously administered with peptides (stocks prepared in phosphate-buffer, pH 7.4) at 250 µg/kg b.w. Blood samples were withdrawn from orbital sinus at following time points post dosing: 1, 3, 5, 24, 26, 28, and 48 h. The samples were collected in pre-labelled microcentrifuge tubes containing anticoagulant (K_2EDTA-2 mg/mL blood). Rat plasma was separated from whole blood by centrifugation for 10 min at 1000-2000 *g* in a refrigerated centrifuge and stored at -80ºC until analysis.

Plasma samples were thawed, and rat growth hormone ELISA assay was performed strictly according to the protocol provided in the assay kit by Invitrogen (catalogue no. KRC5311). The growth hormone levels in ng/mL were obtained from a five-point standard curve using standard rat growth hormone provided with the ELISA kit.

Toxicity assessment in rats

Animal care was conducted according to guidelines and protocols from the IEAC and CPCSEA. Animals were housed individually in Central Animal Facility, IISc. After procuring, the animals were kept for acclimatization for 3-7 days in LD cycle and temperature-controlled rooms. Access to food and water was provided ad libitum. For assessing peptide induced acute toxicity in male Sprague-Dawley rats (230-250 g) were subcutaneously administered with peptides (stocks prepared in phosphate-buffer, pH 7.4) at 250 µg/kg b.w. (X) and 10 mg/kg b.w. (40X). The animals were monitored for the next 14 days. On 14th day, blood samples were withdrawn via cardiac puncture. Serum from whole blood was isolated and standard serum biochemistry analyses were performed.

For all the above-mentioned in vivo studies, the animals were euthanized ethically in CO_2 chamber following CPCSEA guidelines.

a

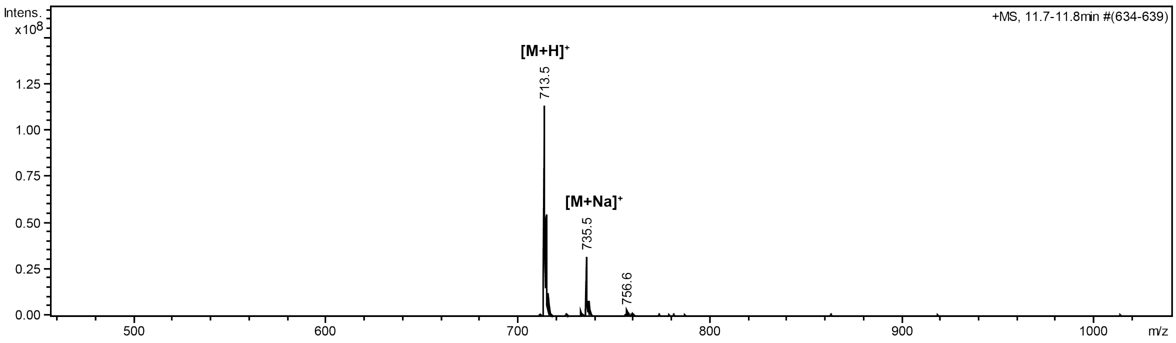

b

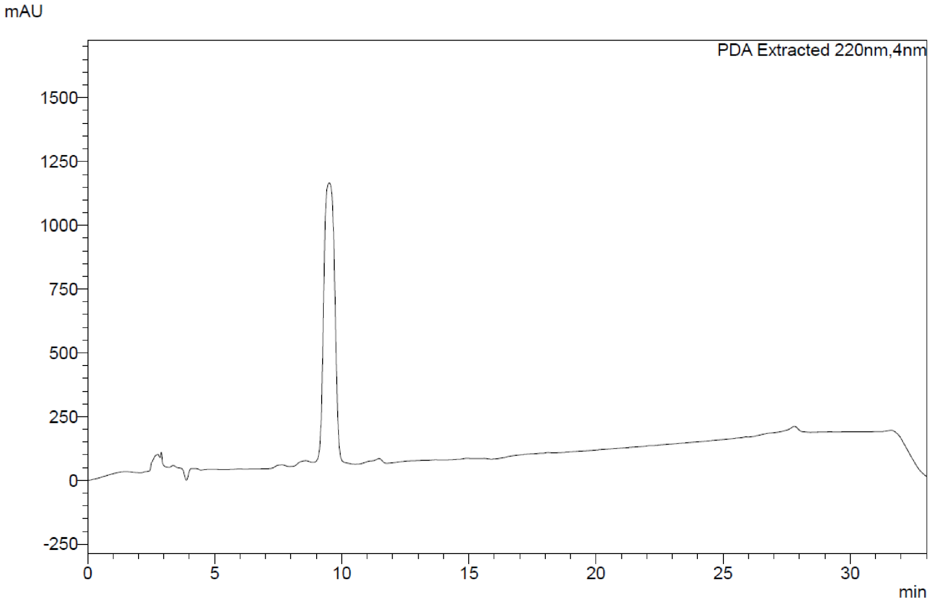

Supplementary Figure 28. Purity of macrocyclic peptides. ESI-MS (**a**) and HPLC purity profile (**b**) of peptide **10**.

a

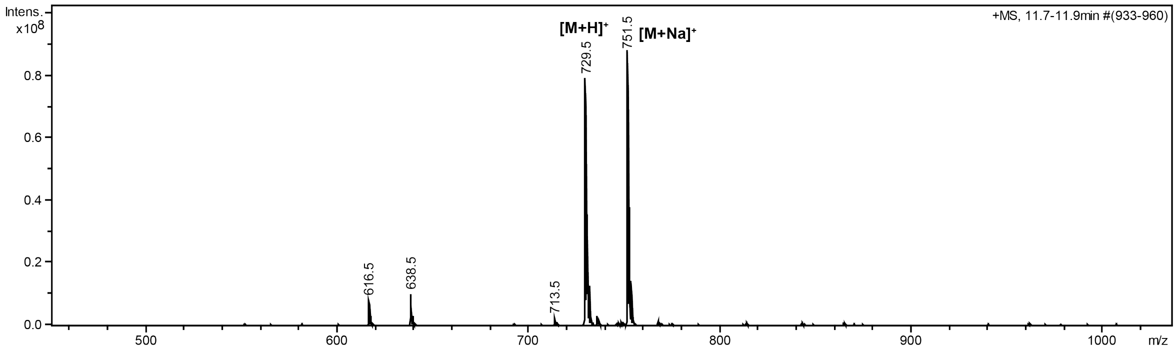

b

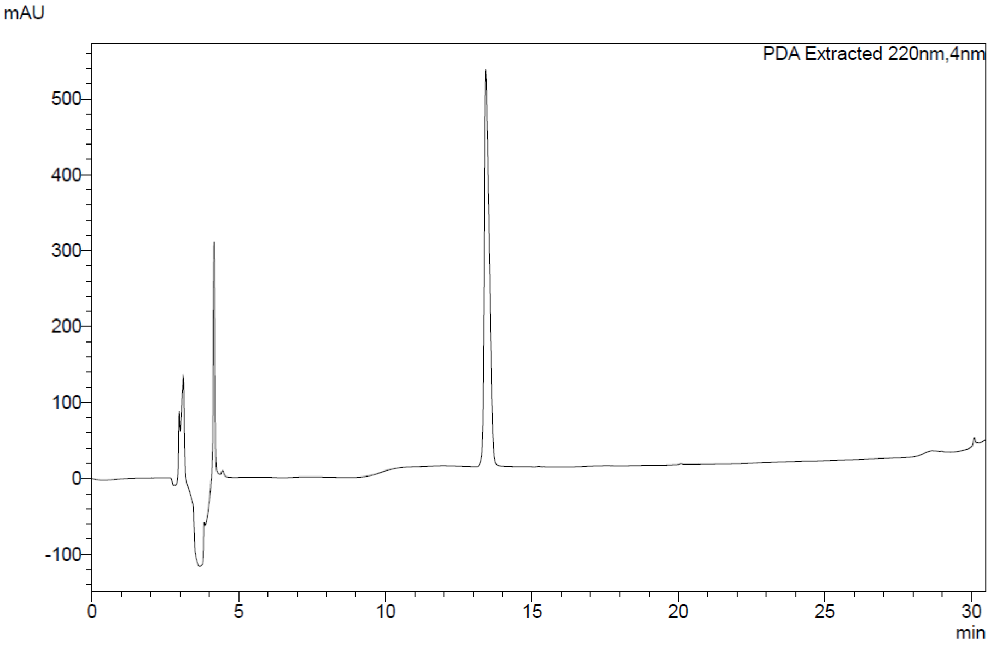

Supplementary Figure 29. Purity of macrocyclic peptides. ESI-MS (**a**) and HPLC purity profile (**b**) of peptide **10a**.

a

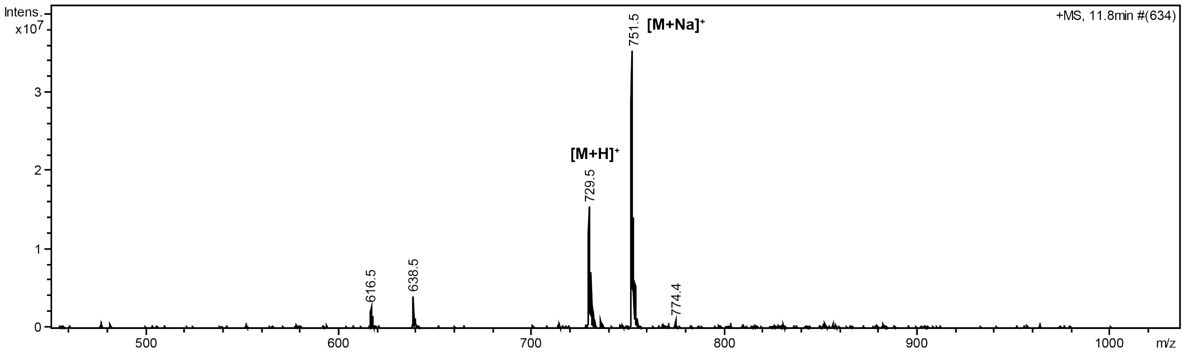

b

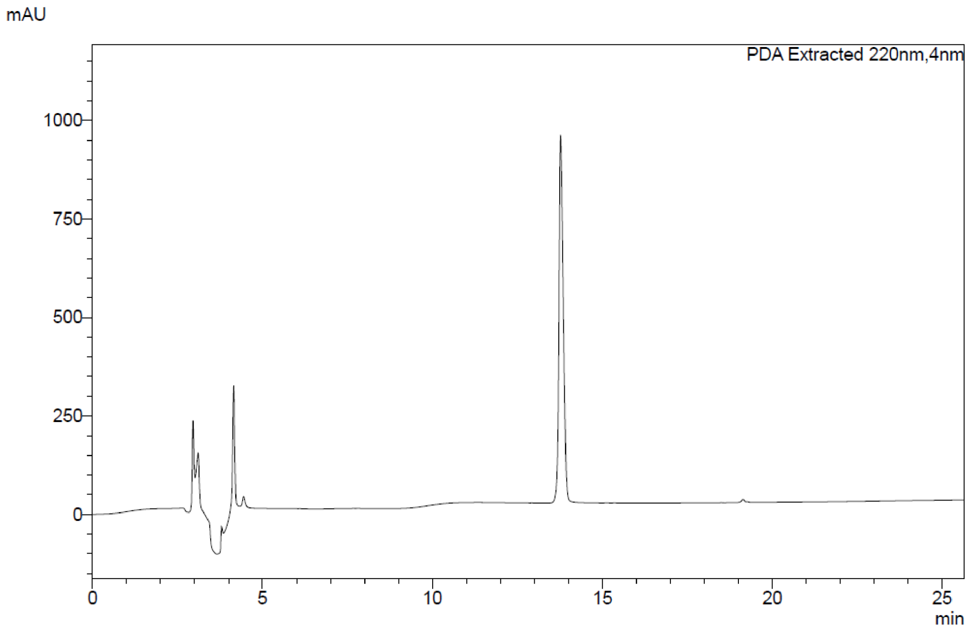

Supplementary Figure 30. Purity of macrocyclic peptides. ESI-MS (**a**) and HPLC purity profile (**b**) of peptide **10b**.

a

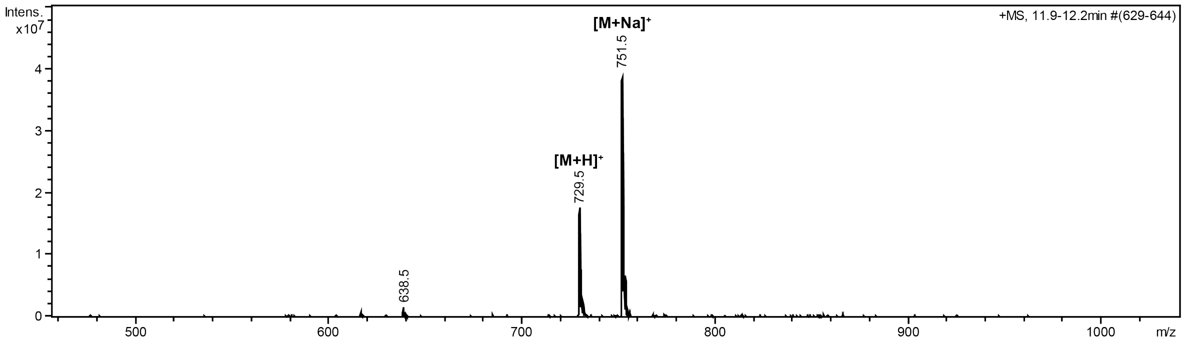

b

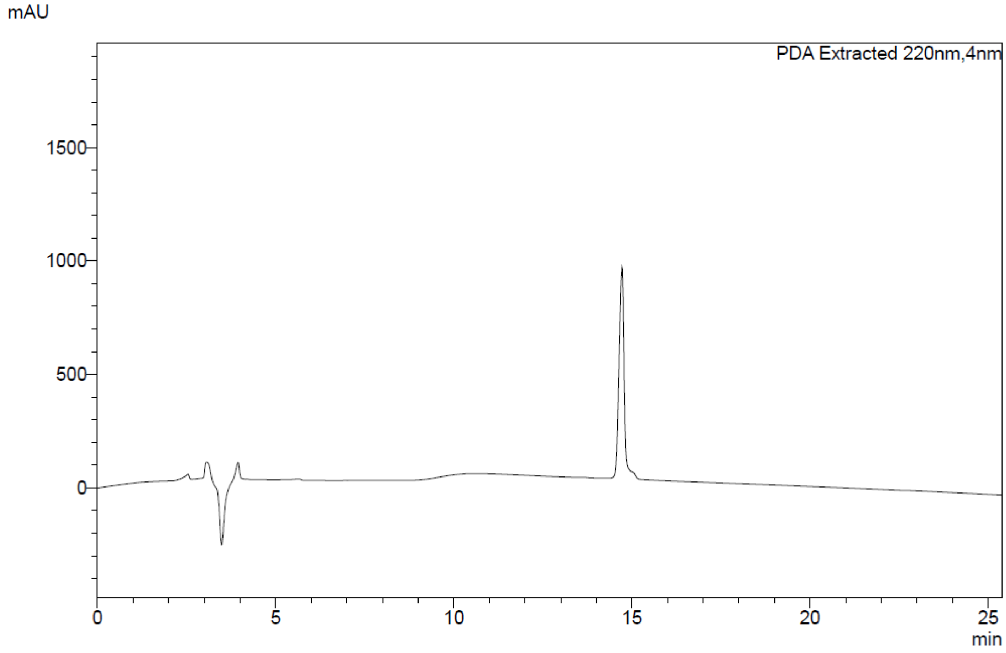

Supplementary Figure 31. Purity of macrocyclic peptides. ESI-MS (**a**) and HPLC purity profile (**b**) of peptide **10c**.

a

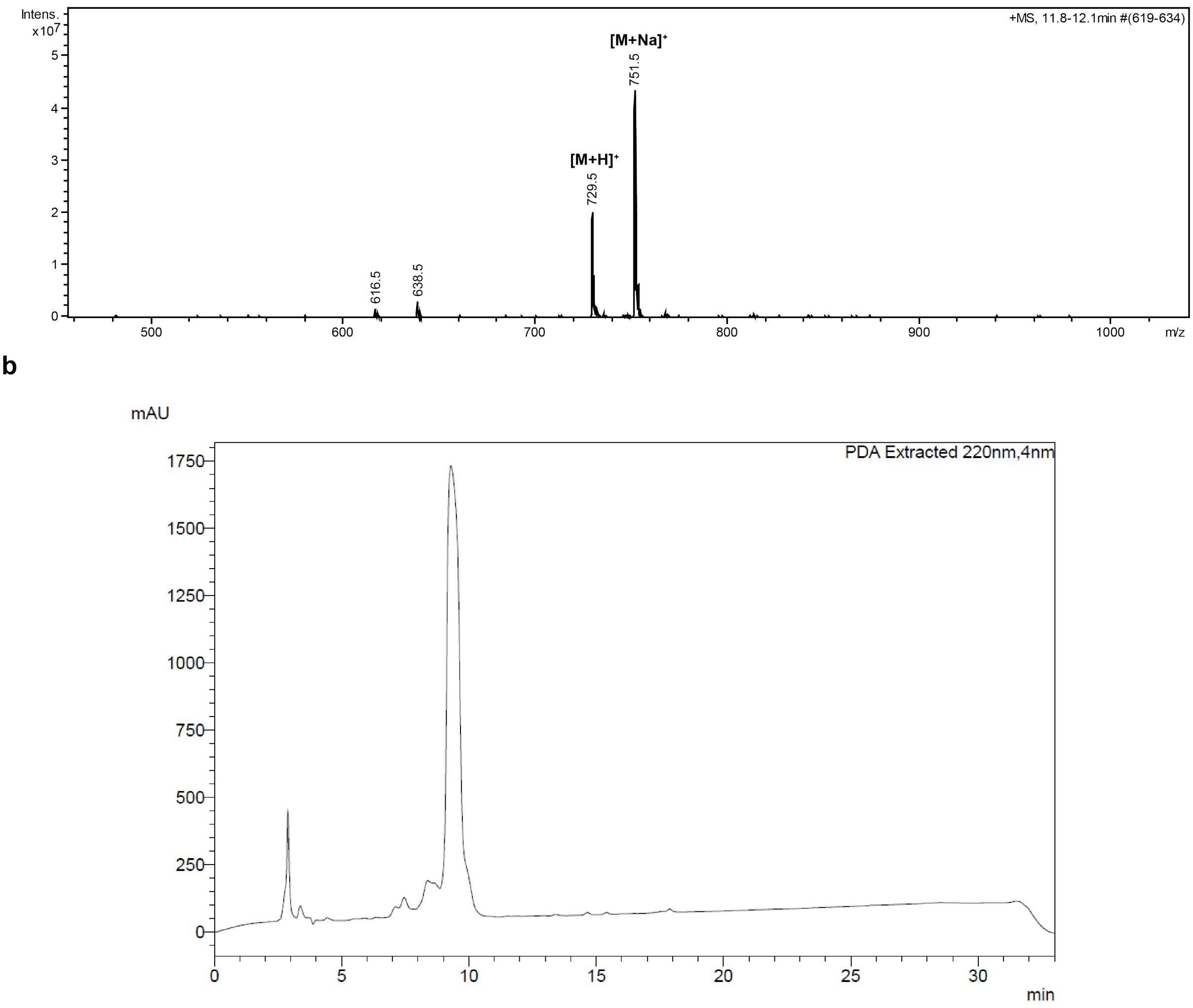

Supplementary Figure 32. Purity of macrocyclic peptides. ESI-MS (**a**) and HPLC purity profile (**b**) of peptide **10d**.

a

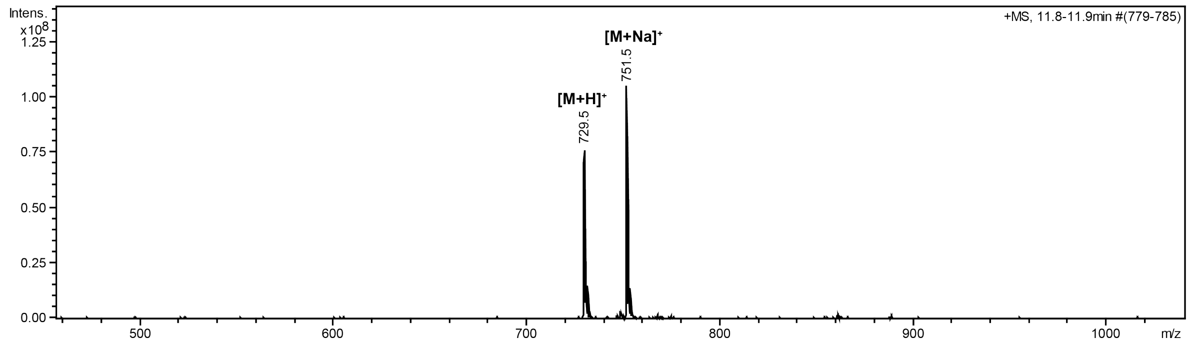

b

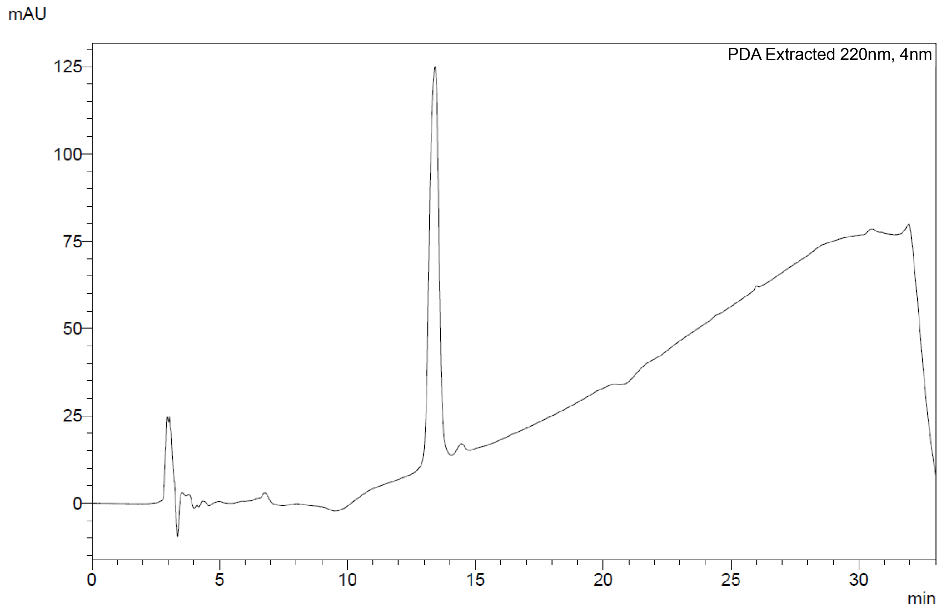

Supplementary Figure 33. Purity of macrocyclic peptides. ESI-MS (**a**) and HPLC purity profile (**b**) of peptide **10e**.

a

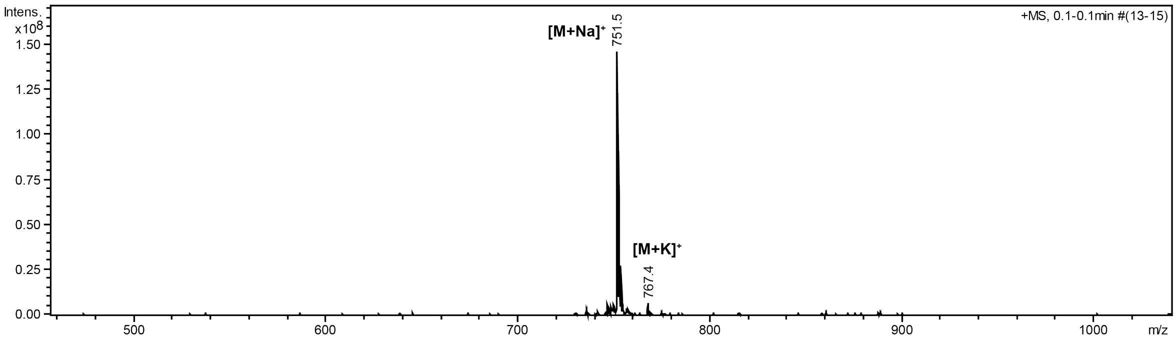

b

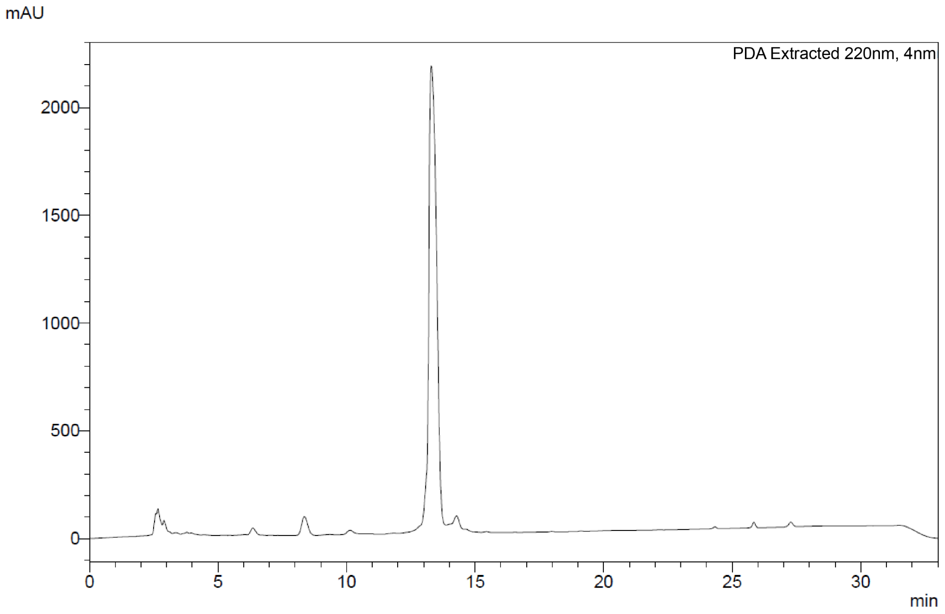

Supplementary Figure 34. Purity of macrocyclic peptides. ESI-MS (**a**) and HPLC purity profile (**b**) of peptide **10f.**

a

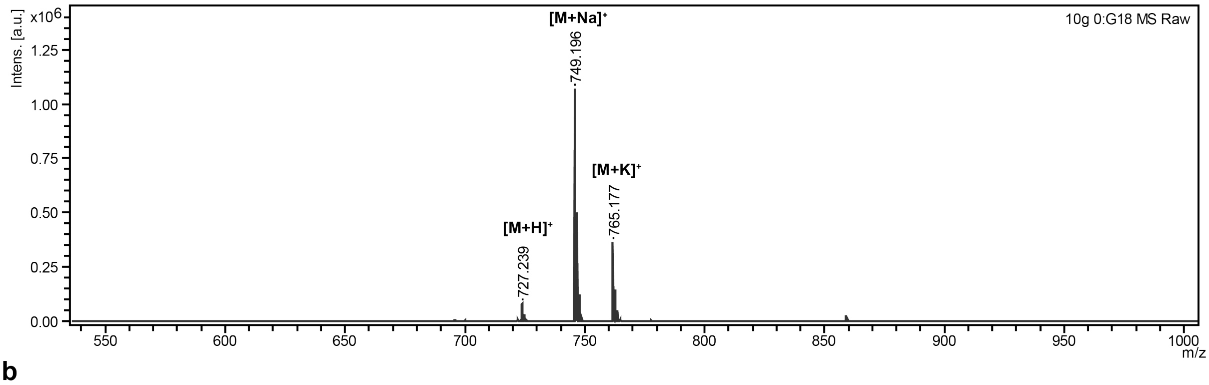

b

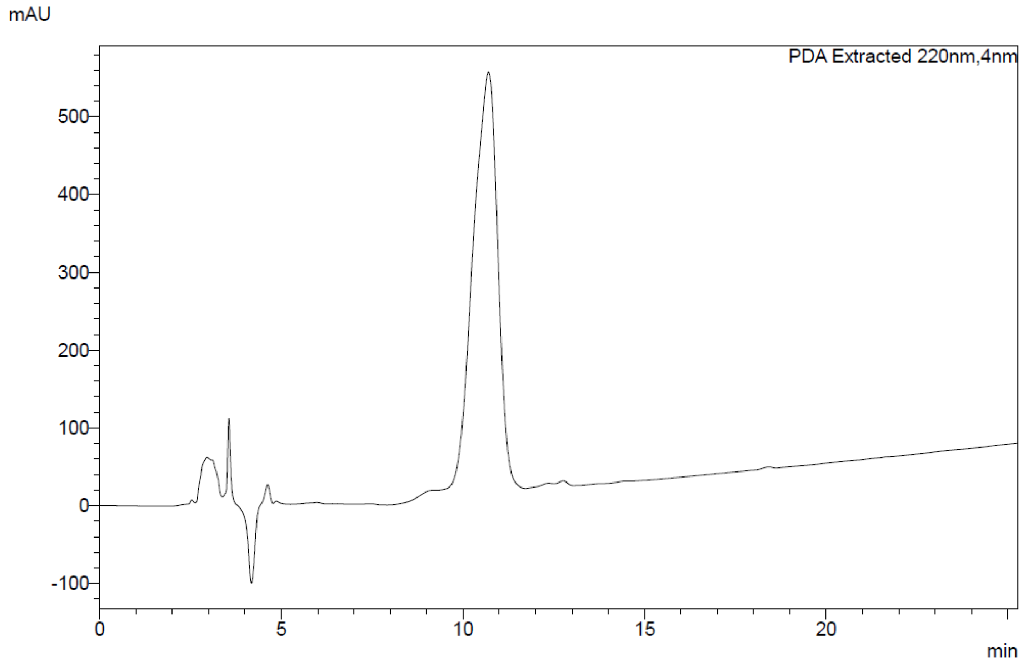

Supplementary Figure 35. Purity of macrocyclic peptides. ESI-MS (**a**) and HPLC purity profile (**b**) of peptide **10g.**

a

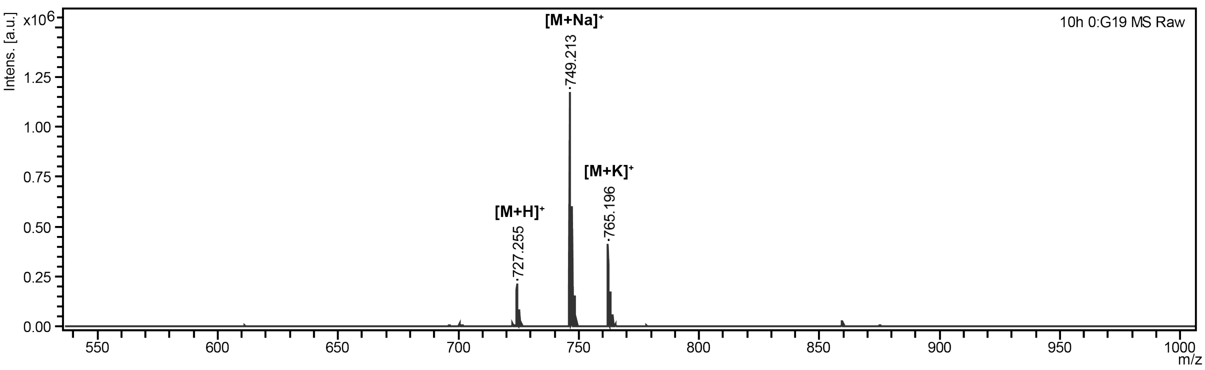

b

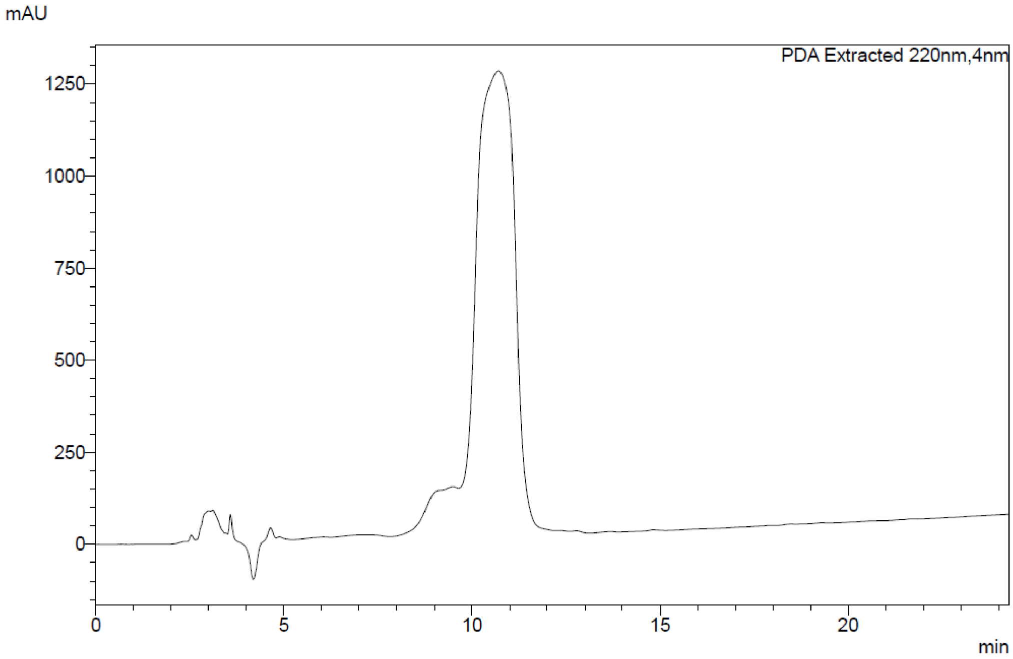

Supplementary Figure 36. Purity of macrocyclic peptides. ESI-MS (**a**) and HPLC purity profile (**b**) of peptide **10h.**

a

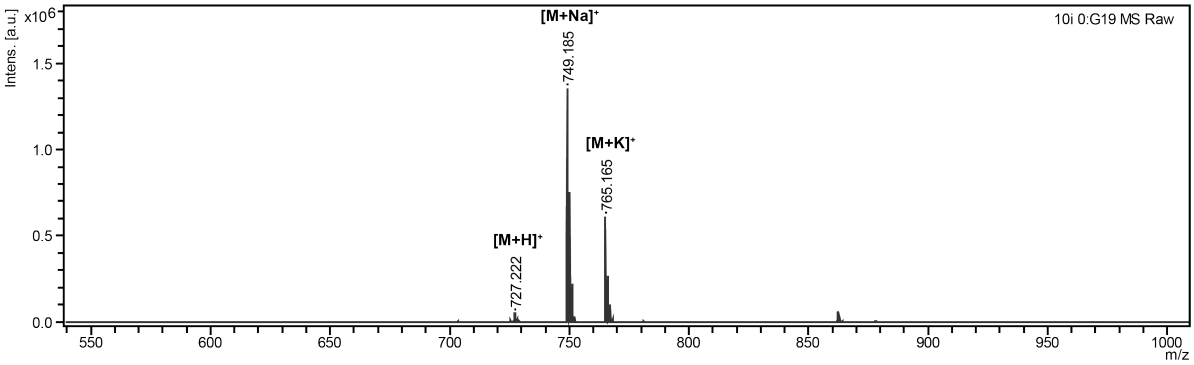

b

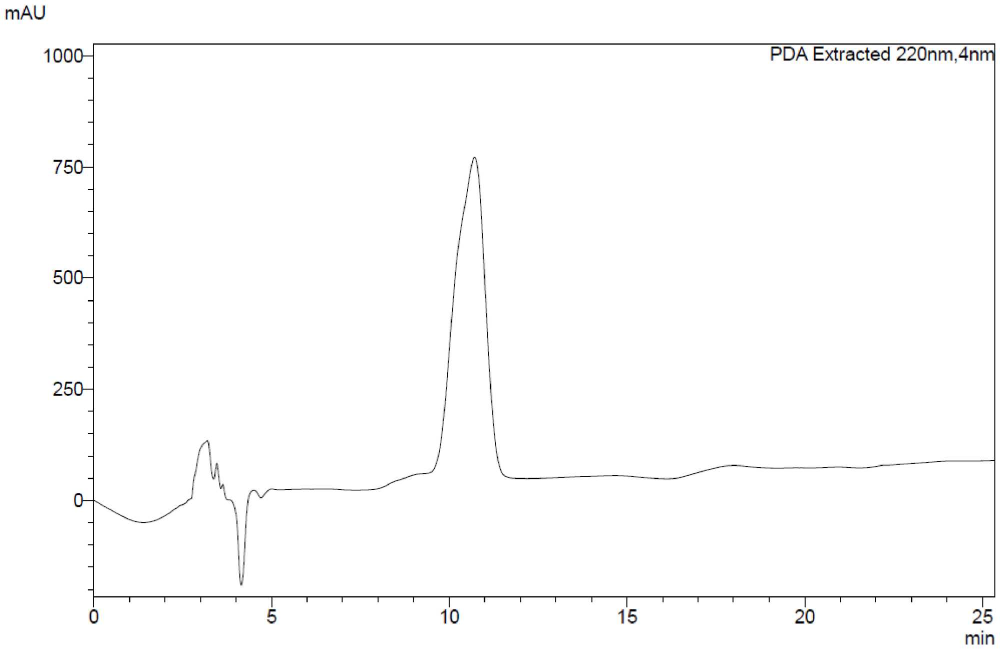

Supplementary Figure 37. Purity of macrocyclic peptides. ESI-MS (**a**) and HPLC purity profile (**b**) of peptide **10i.**

a

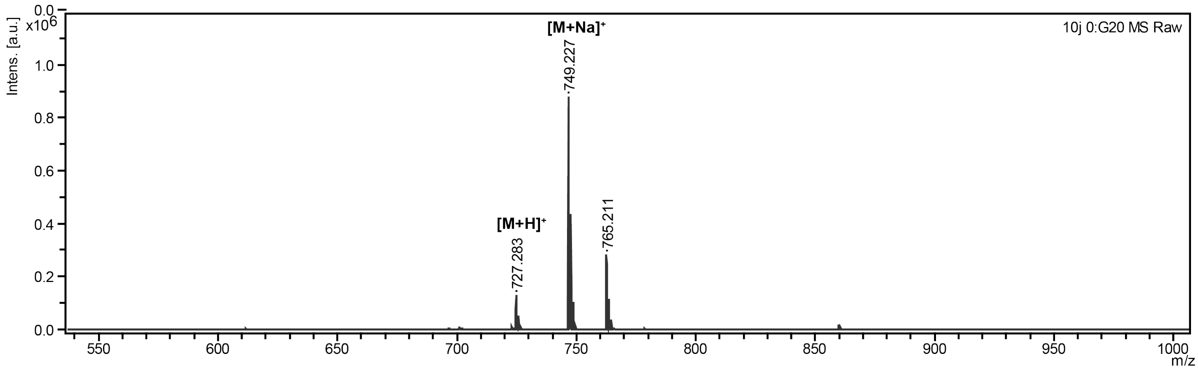

b

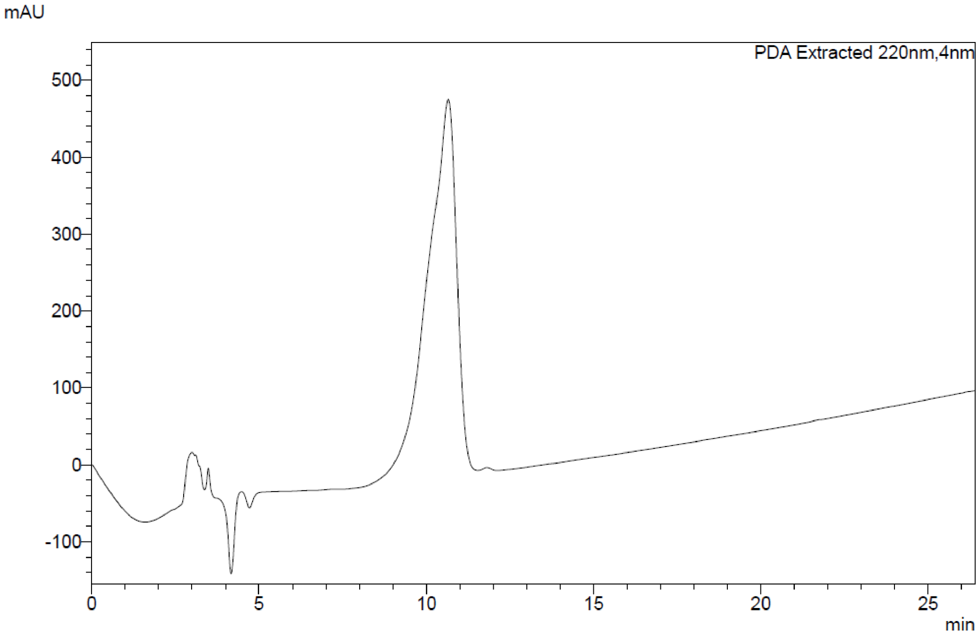

Supplementary Figure 38. Purity of macrocyclic peptides. ESI-MS (**a**) and HPLC purity profile (**b**) of peptide **10j.**

a

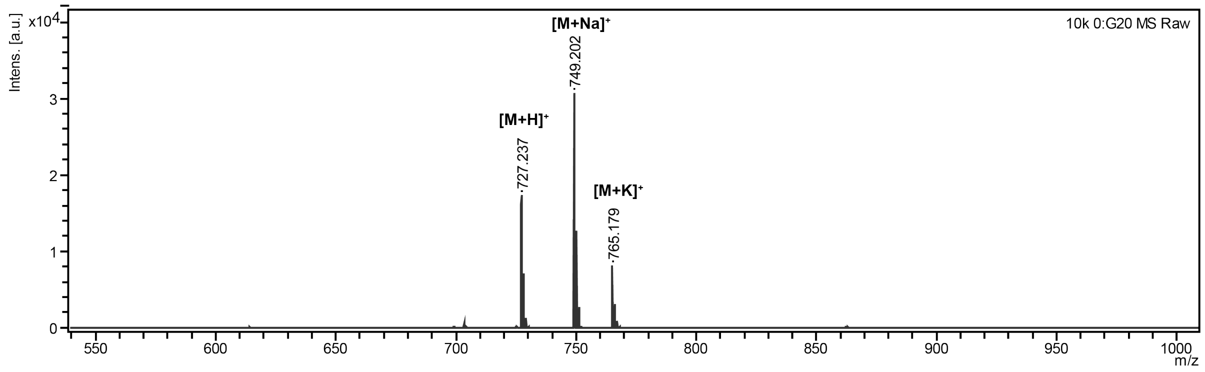

b

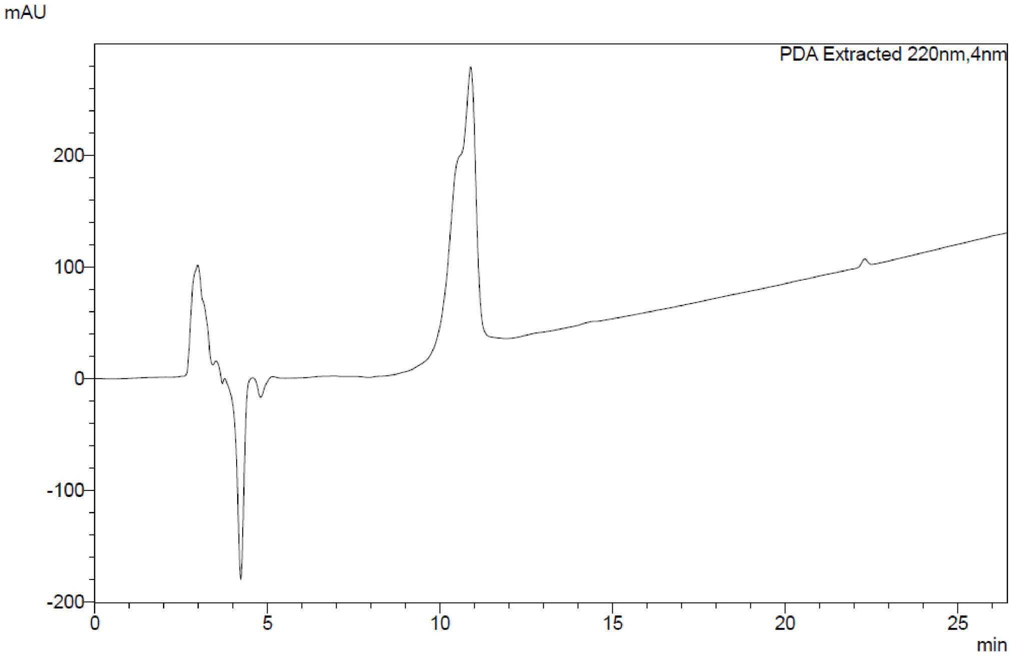

Supplementary Figure 39. Purity of macrocyclic peptides. ESI-MS (**a**) and HPLC purity profile (**b**) of peptide **10k.**

a

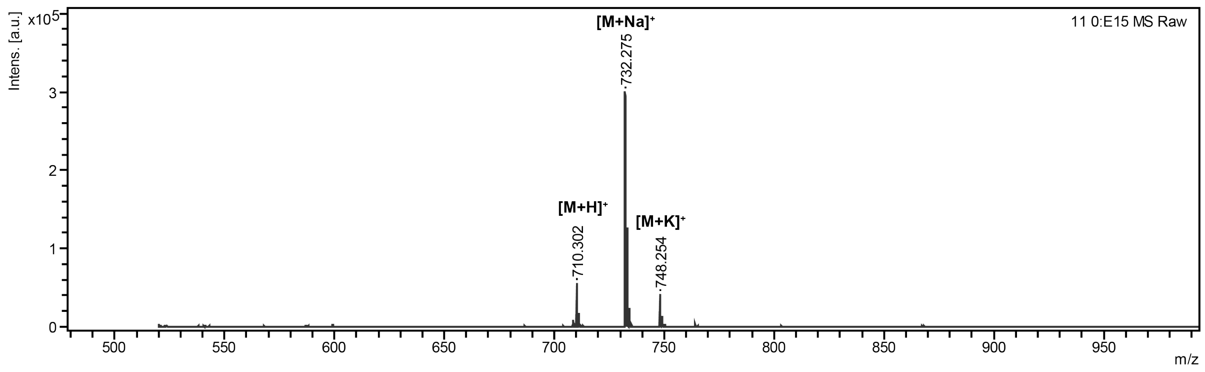

b

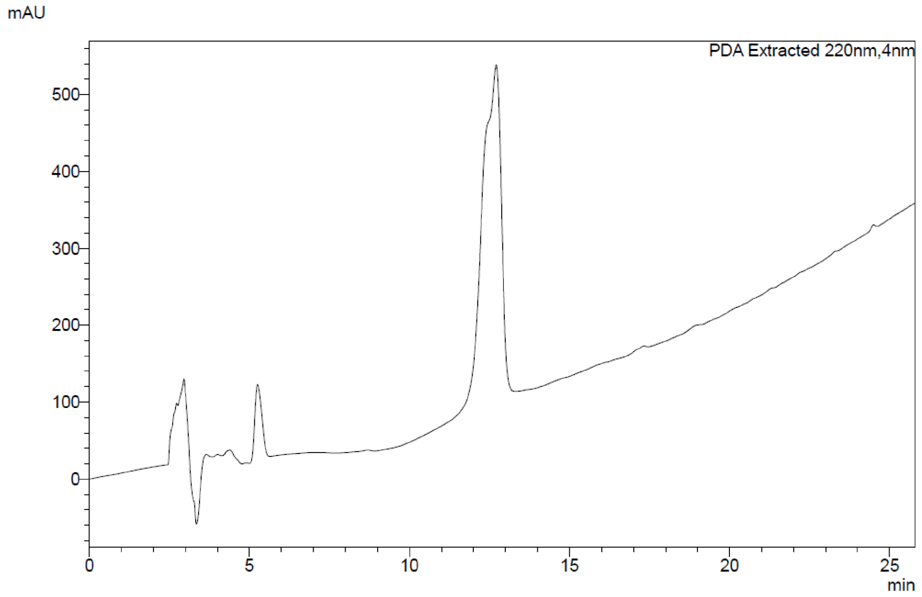

Supplementary Figure 40. Purity of macrocyclic peptides. ESI-MS (**a**) and HPLC purity profile (**b**) of peptide **11.**

a

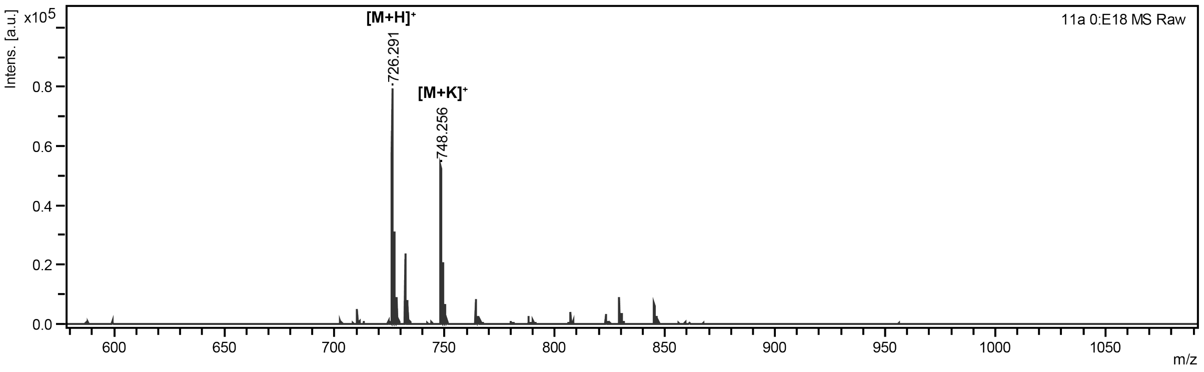

b

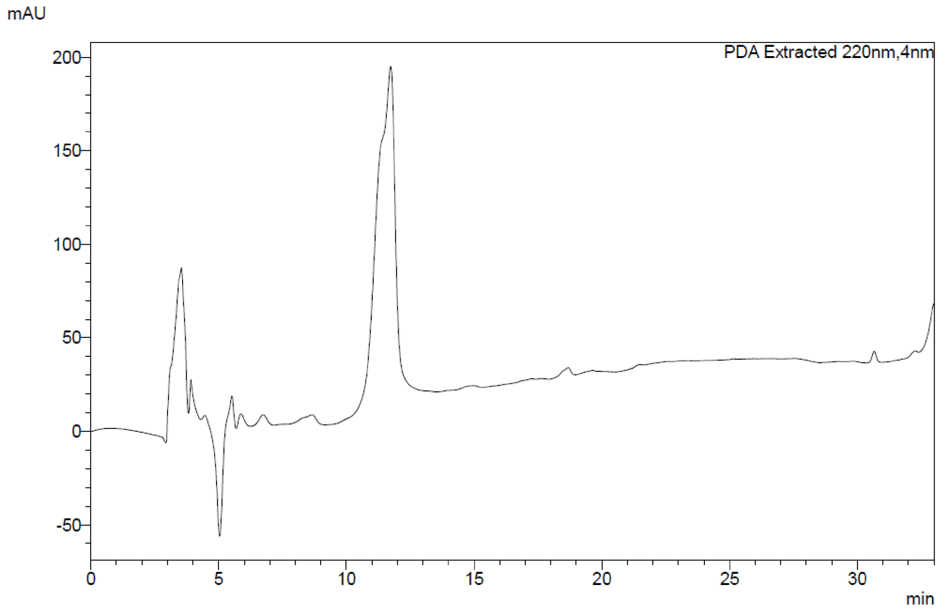

Supplementary Figure 41. Purity of macrocyclic peptides. ESI-MS (**a**) and HPLC purity profile (**b**) of peptide **11a.**

a

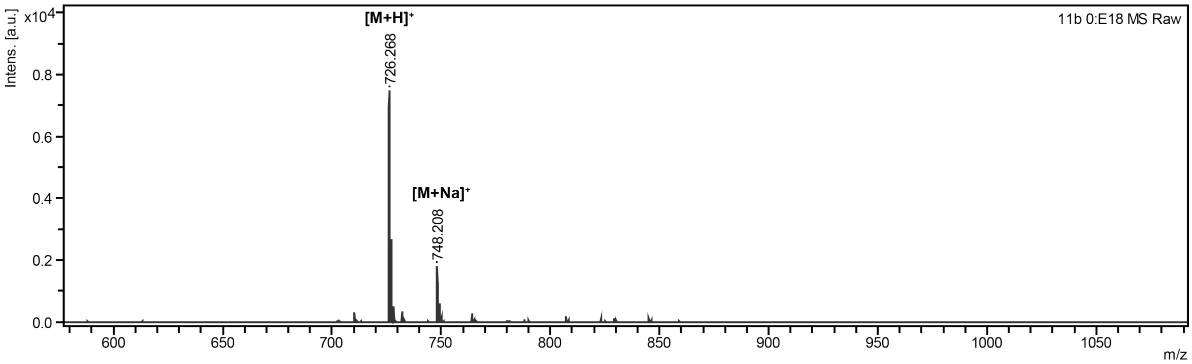

b

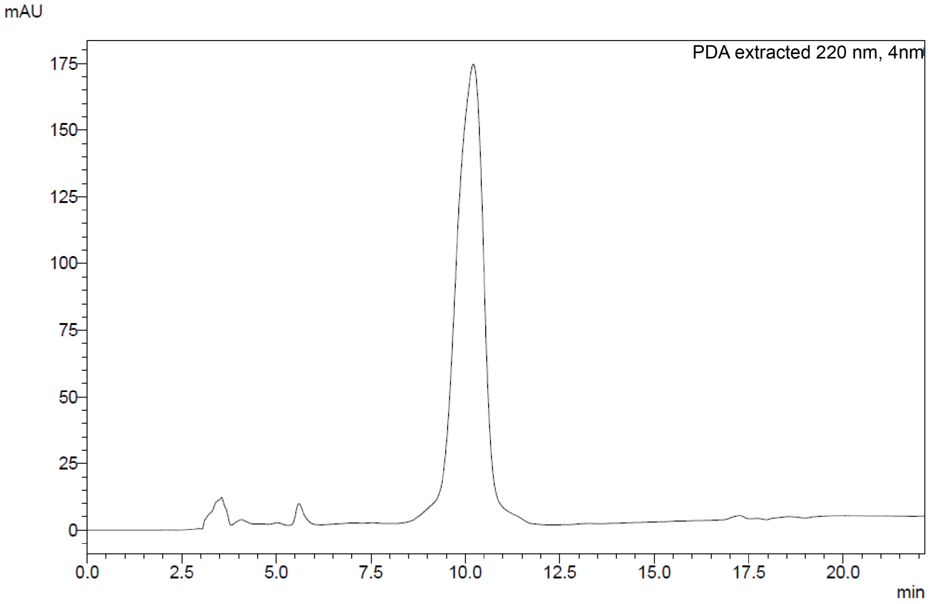

Supplementary Figure 42. Purity of macrocyclic peptides. ESI-MS (**a**) and HPLC purity profile (**b**) of peptide **11b.**

a

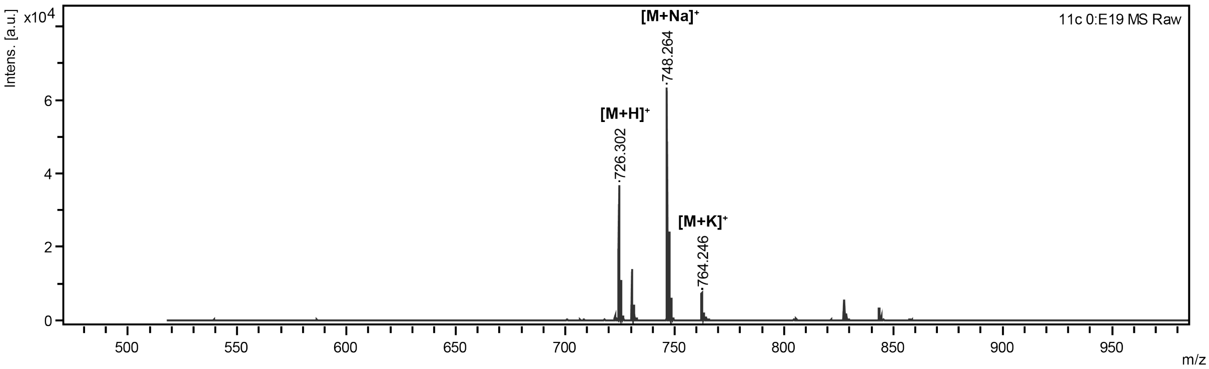

b

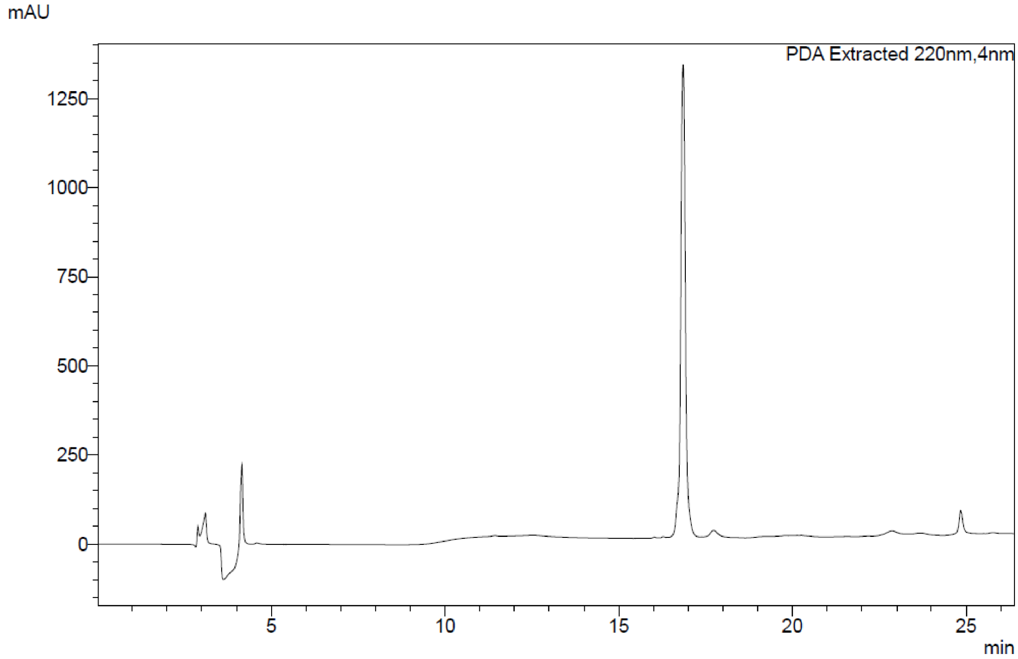

Supplementary Figure 43. Purity of macrocyclic peptides. ESI-MS (**a**) and HPLC purity profile (**b**) of peptide **11c.**

a

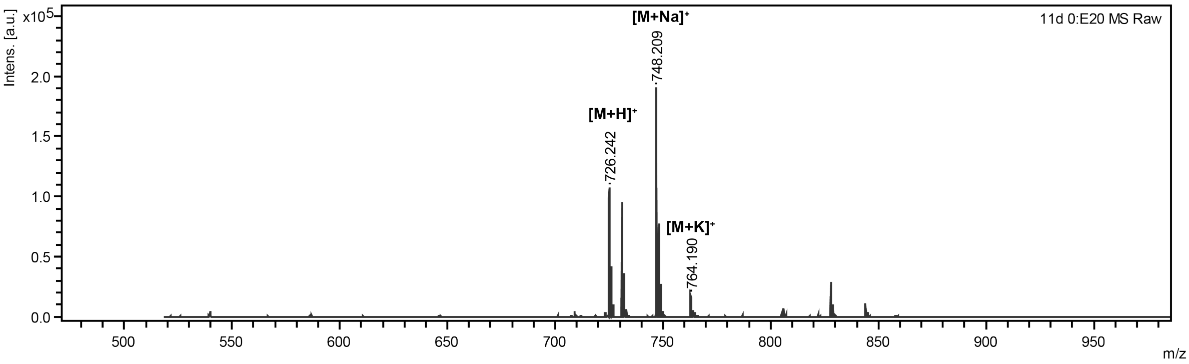

b

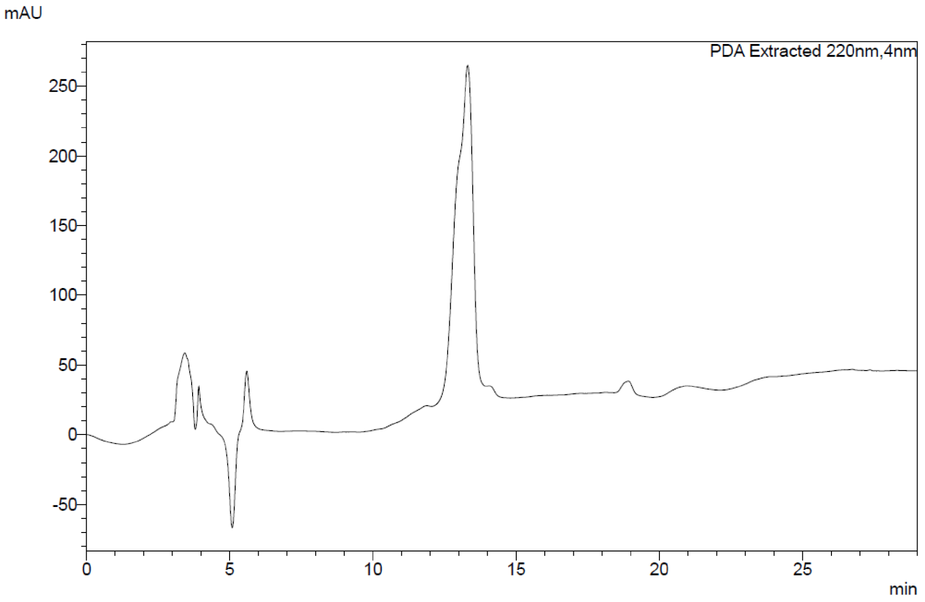

Supplementary Figure 44. Purity of macrocyclic peptides. ESI-MS (**a**) and HPLC purity profile (**b**) of peptide **11d.**

a

b

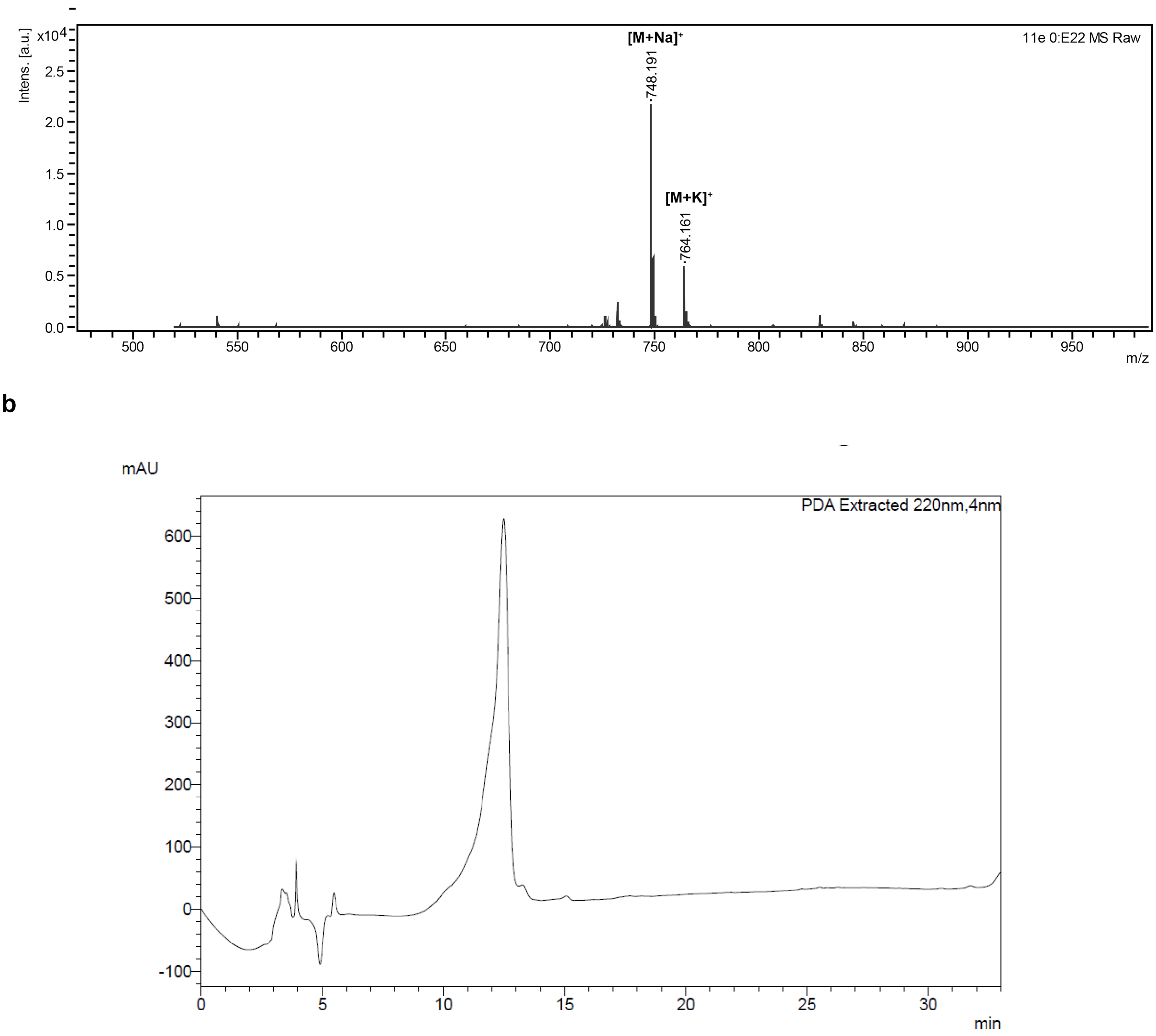

Supplementary Figure 45. Purity of macrocyclic peptides. ESI-MS (**a**) and HPLC purity profile (**b**) of peptide **11e.**

a

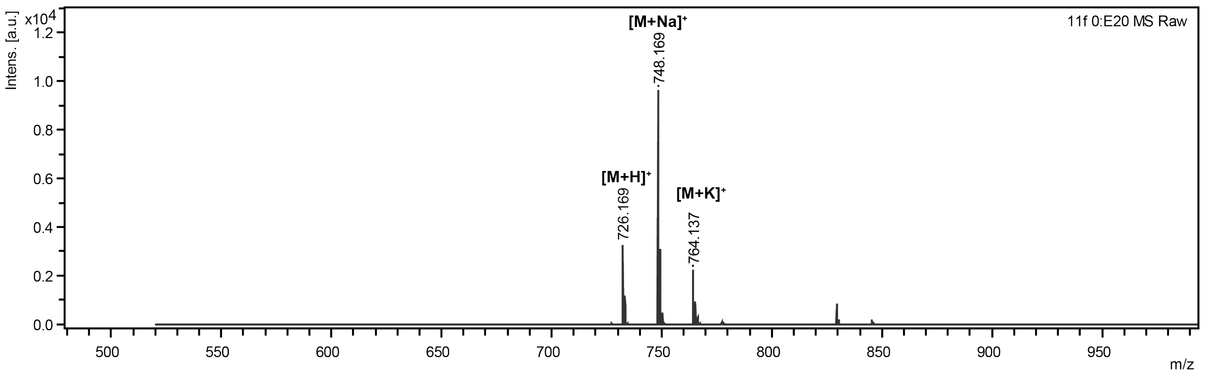

b

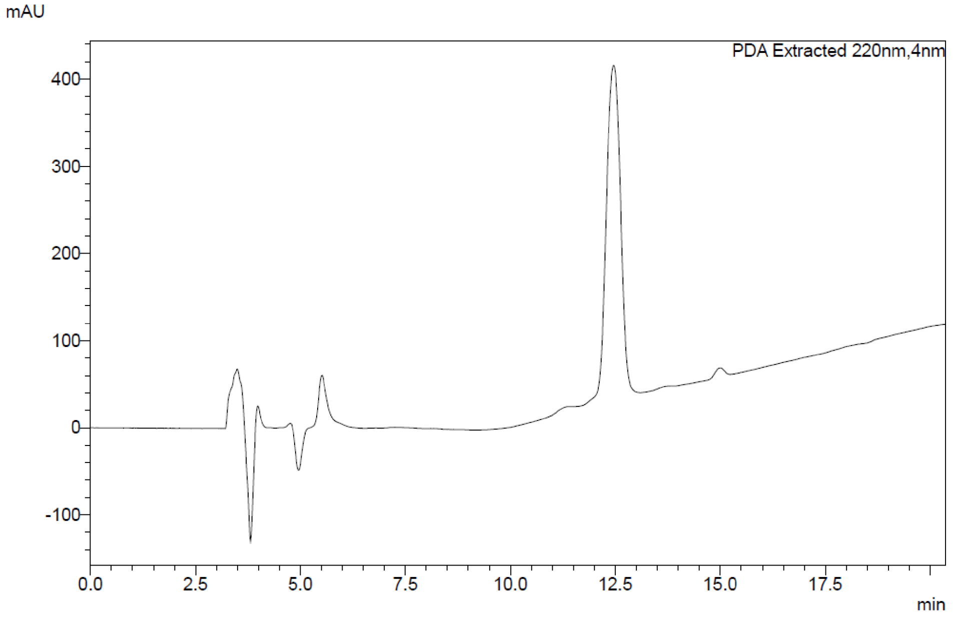

Supplementary Figure 46. Purity of macrocyclic peptides. ESI-MS (**a**) and HPLC purity profile (**b**) of peptide **11f.**

a

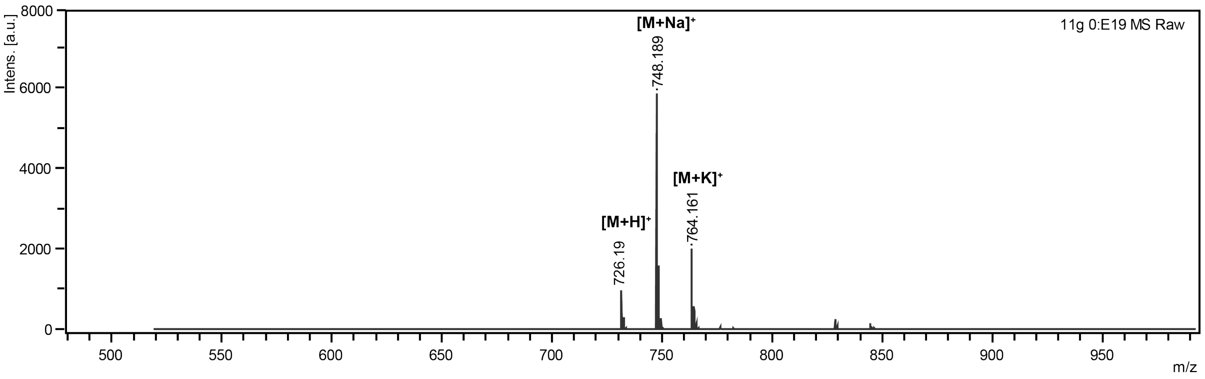

b

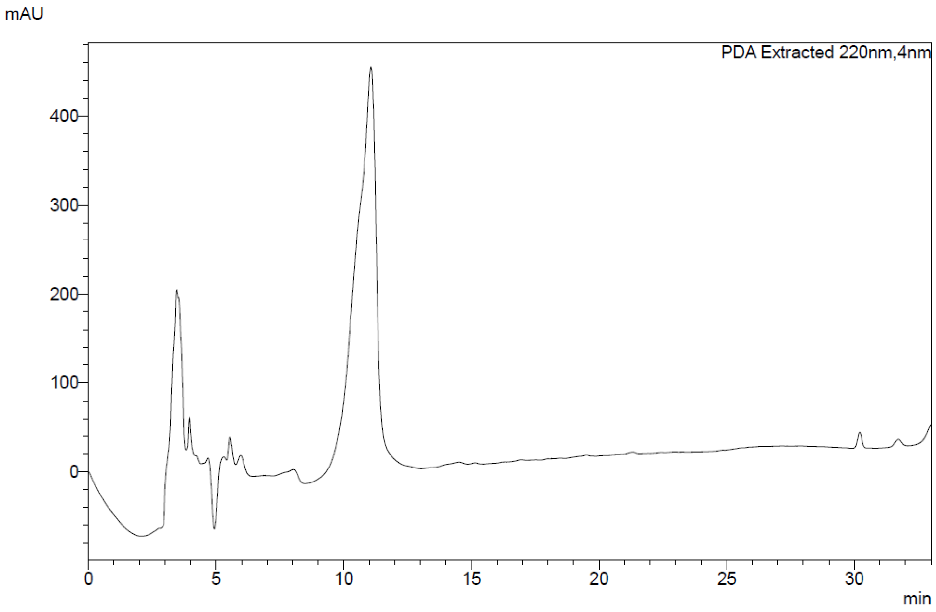

Supplementary Figure 47. Purity of macrocyclic peptides. ESI-MS (**a**) and HPLC purity profile (**b**) of peptide **11g.**

a

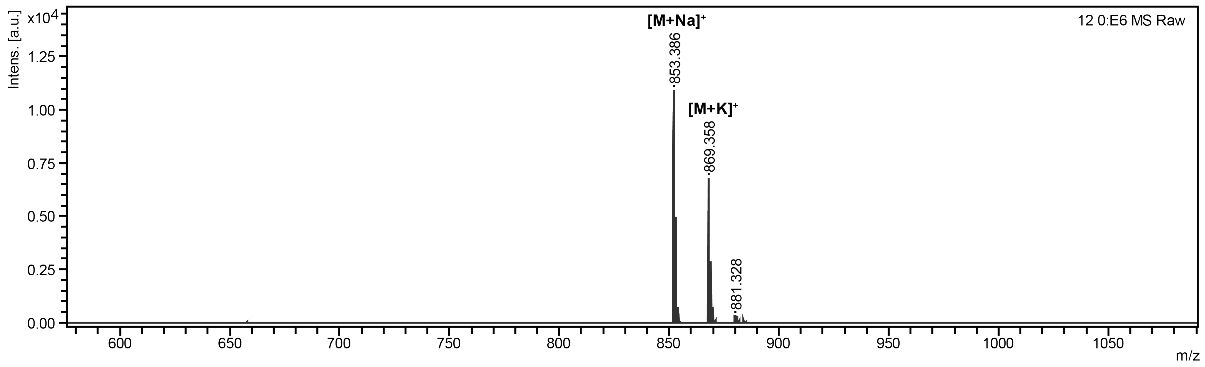

b

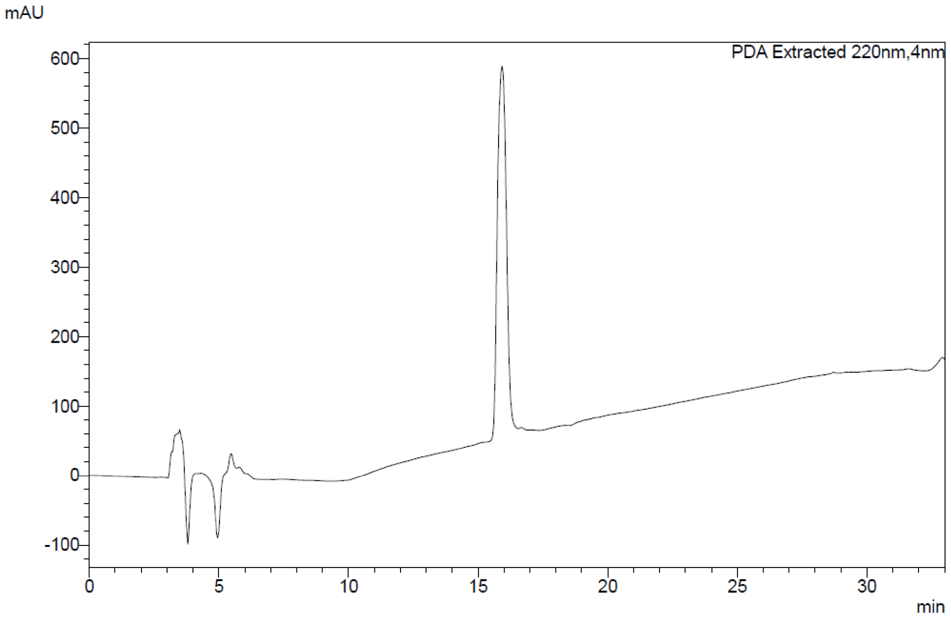

Supplementary Figure 48. Purity of macrocyclic peptides. ESI-MS (**a**) and HPLC purity profile (**b**) of peptide **12.**

a

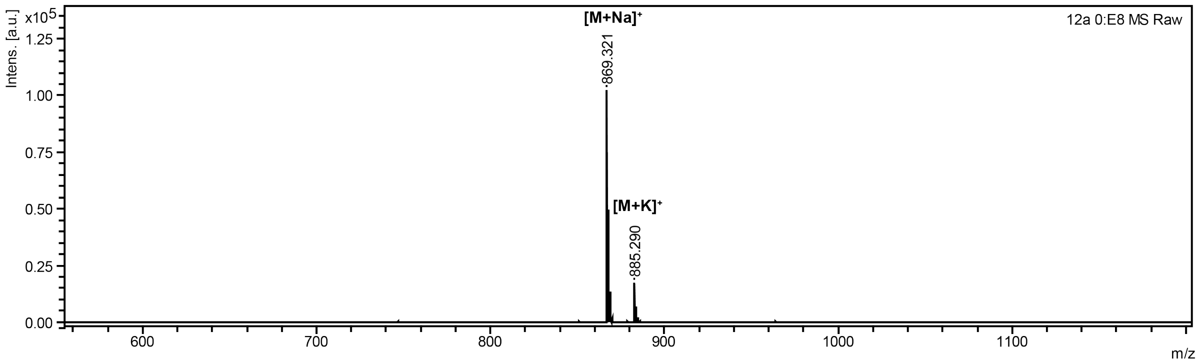

b

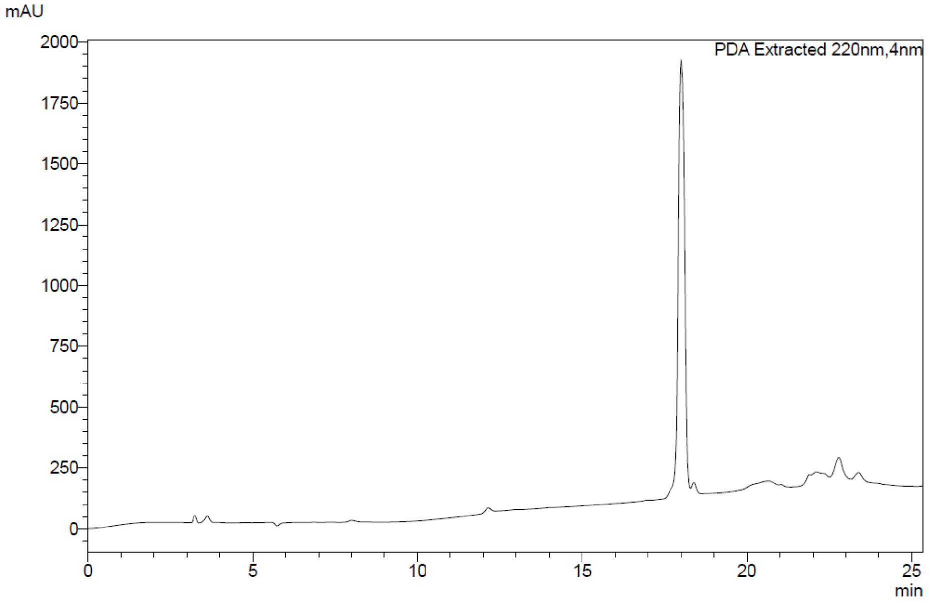

Supplementary Figure 49. Purity of macrocyclic peptides. ESI-MS (**a**) and HPLC purity profile (**b**) of peptide **12a.**

a

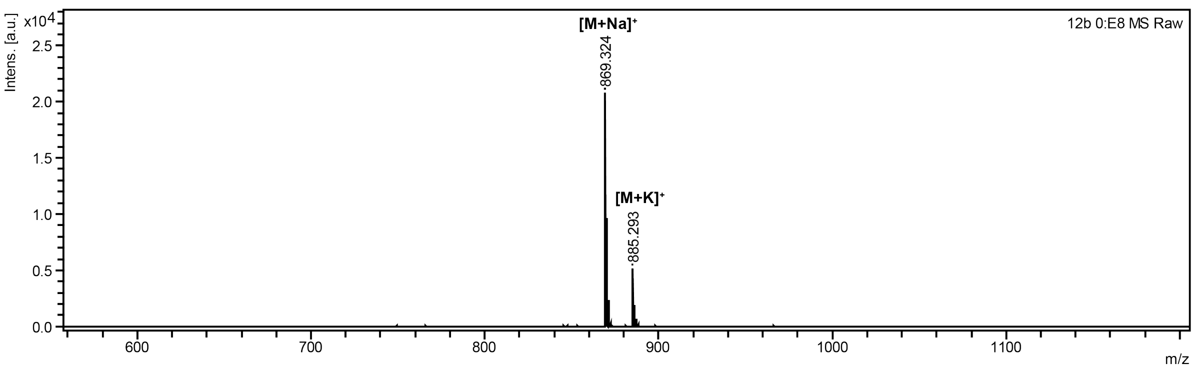

b

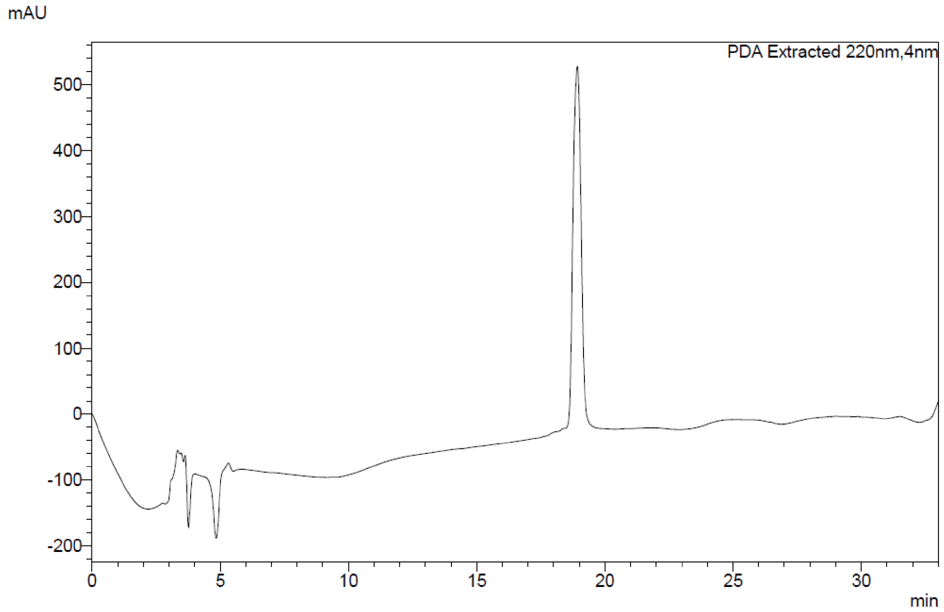

Supplementary Figure 50. Purity of macrocyclic peptides. ESI-MS (**a**) and HPLC purity profile (**b**) of peptide **12b.**

a

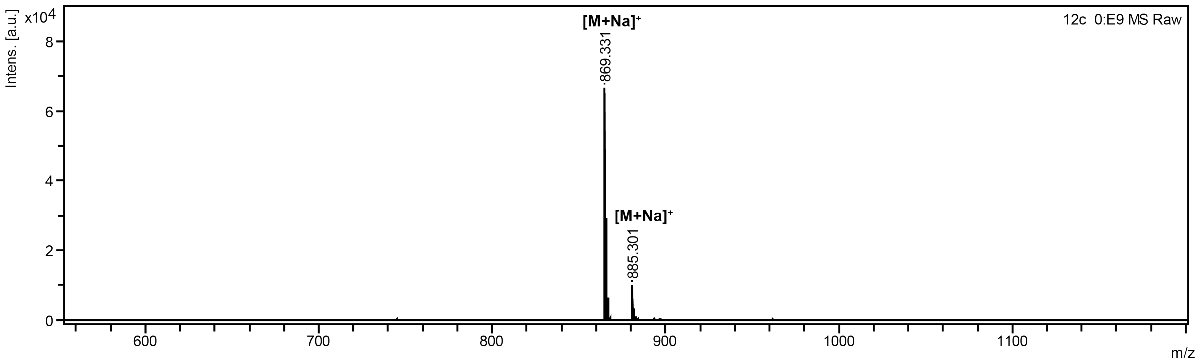

b

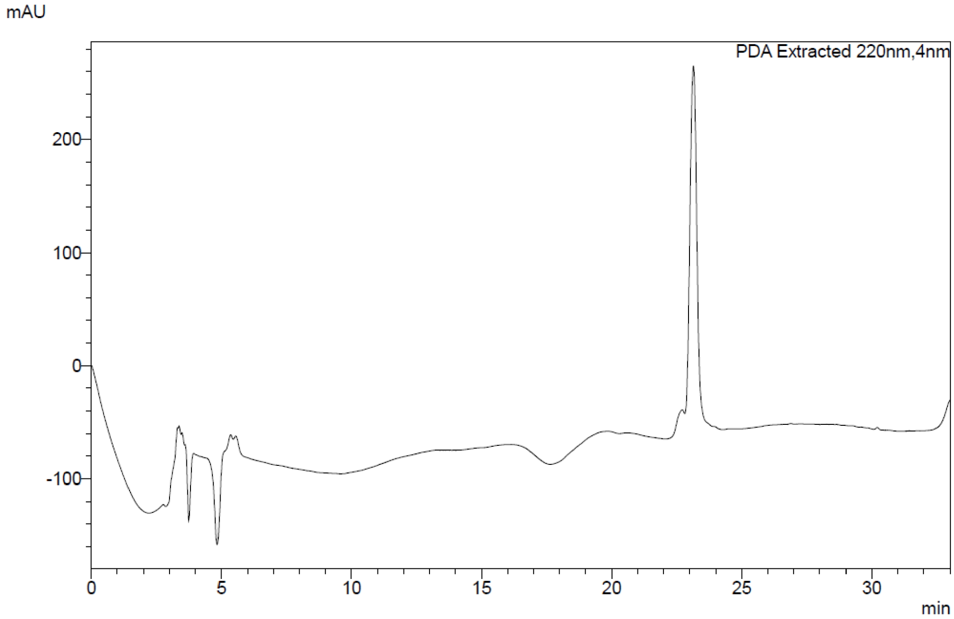

Supplementary Figure 51. Purity of macrocyclic peptides. ESI-MS (**a**) and HPLC purity profile (**b**) of peptide **12c.**

a

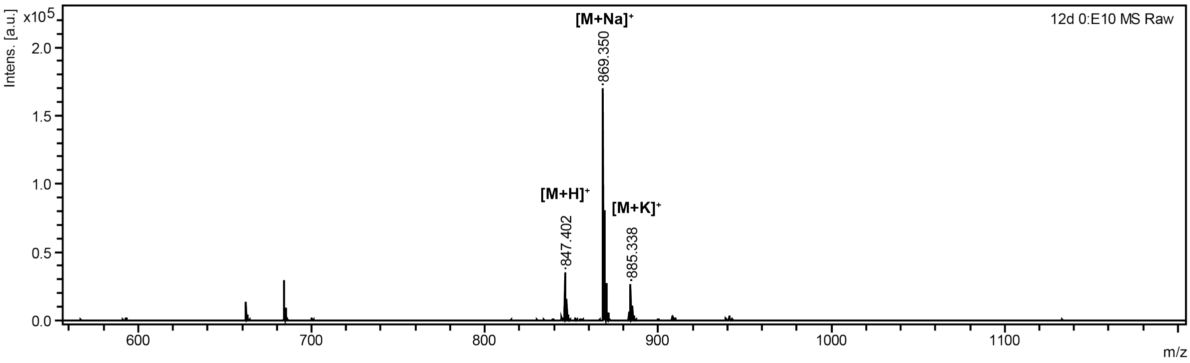

b

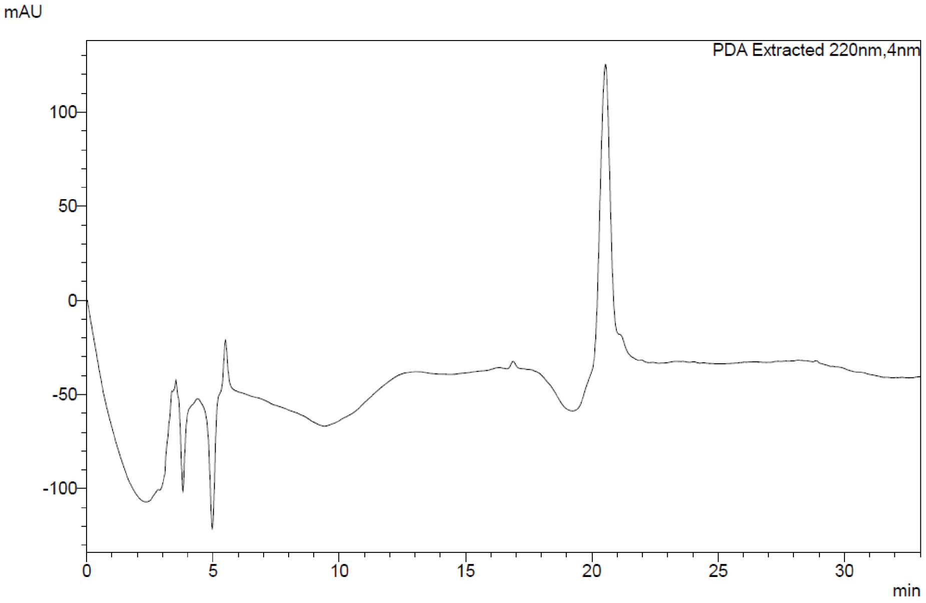

Supplementary Figure 52. Purity of macrocyclic peptides. ESI-MS (**a**) and HPLC purity profile (**b**) of peptide **12d.**

a

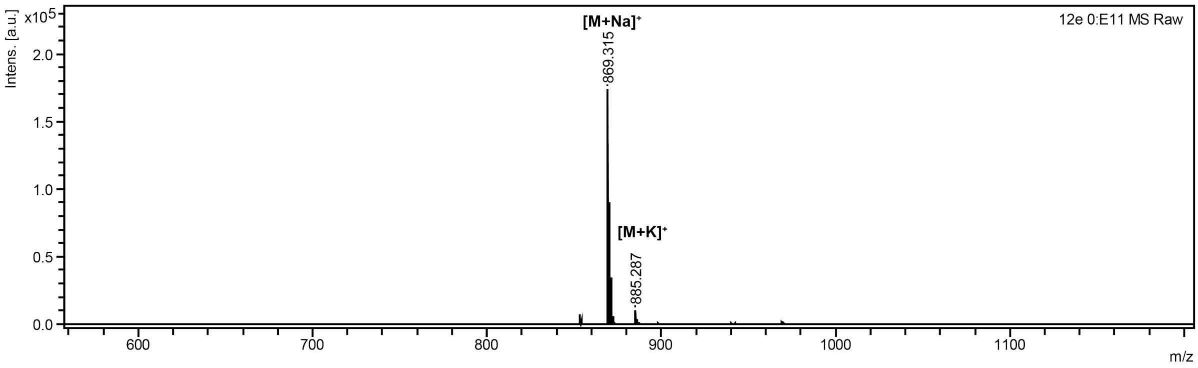

b

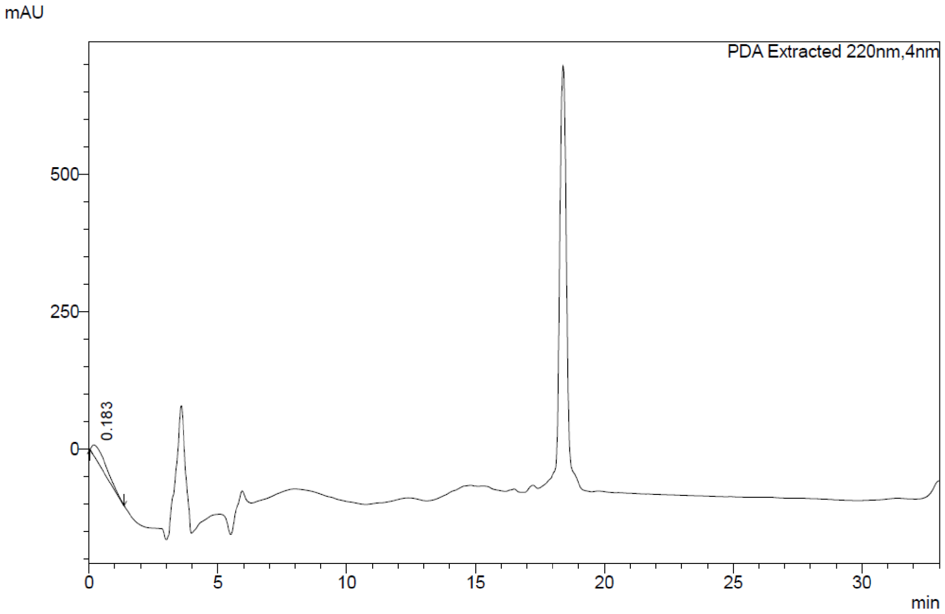

Supplementary Figure 53. Purity of macrocyclic peptides. ESI-MS (**a**) and HPLC purity profile (**b**) of peptide **12e.**

a

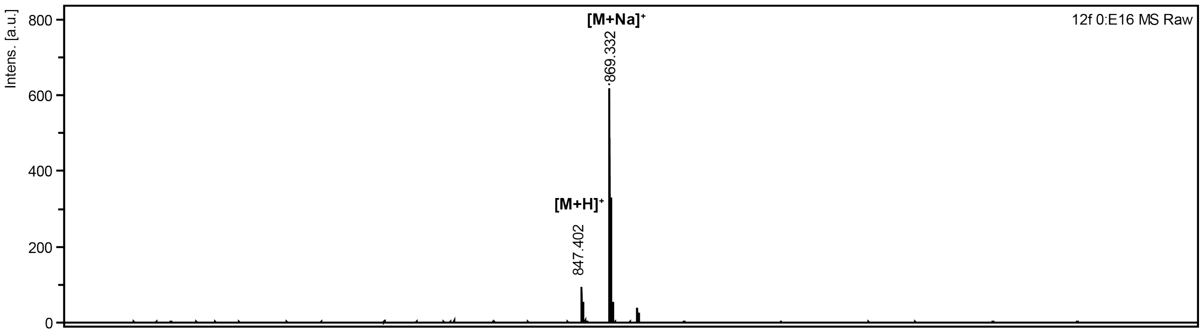

b

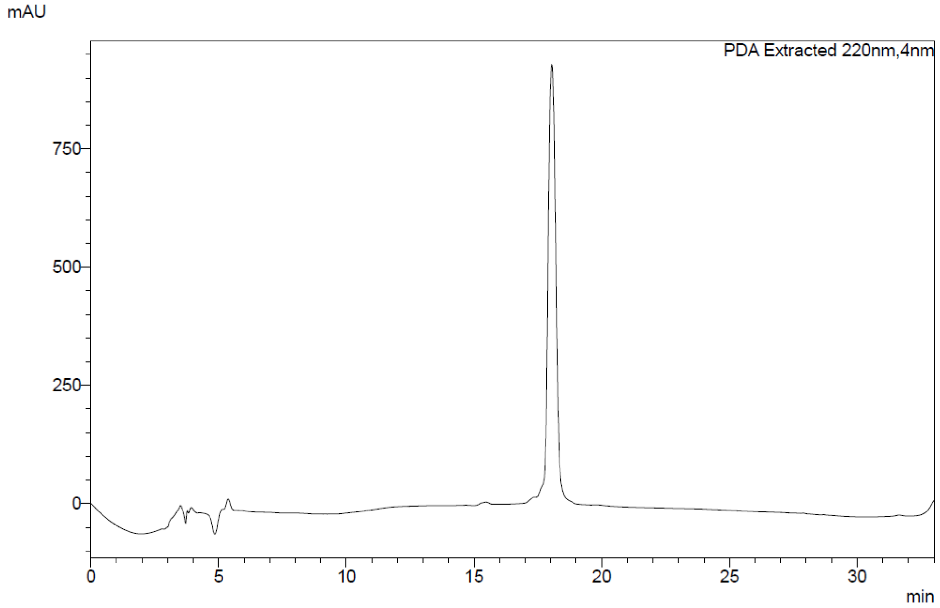

Supplementary Figure 54. Purity of macrocyclic peptides. ESI-MS (**a**) and HPLC purity profile (**b**) of peptide **12f.**

a

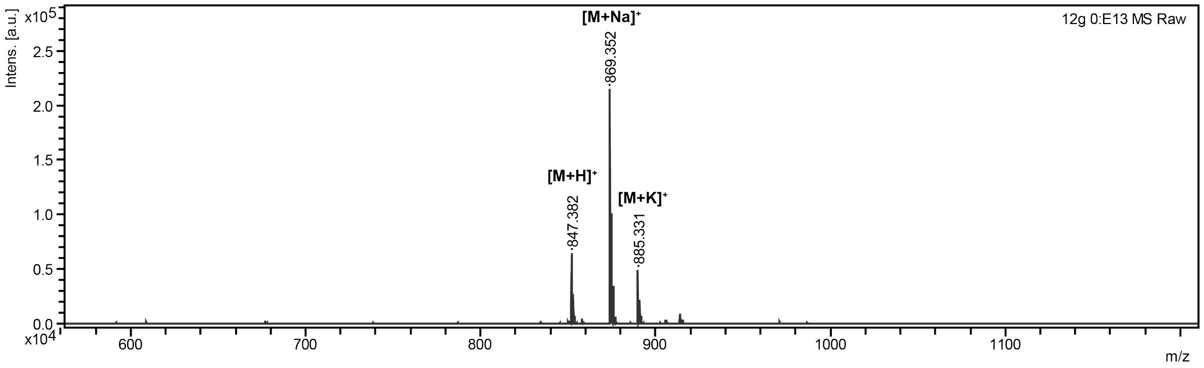

b

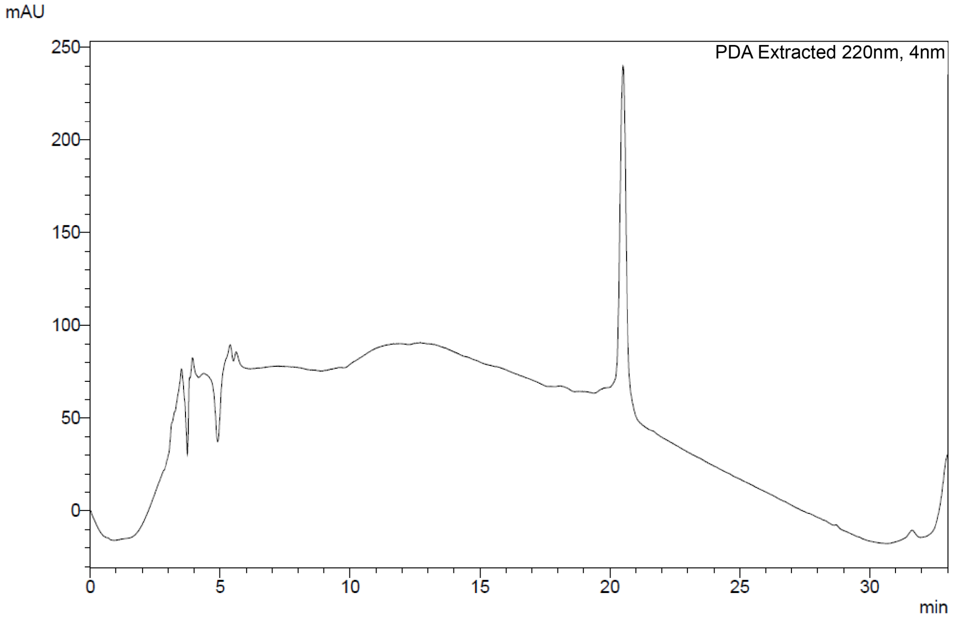

Supplementary Figure 55. Purity of macrocyclic peptides. ESI-MS (**a**) and HPLC purity profile (**b**) of peptide **12g.**

a

b

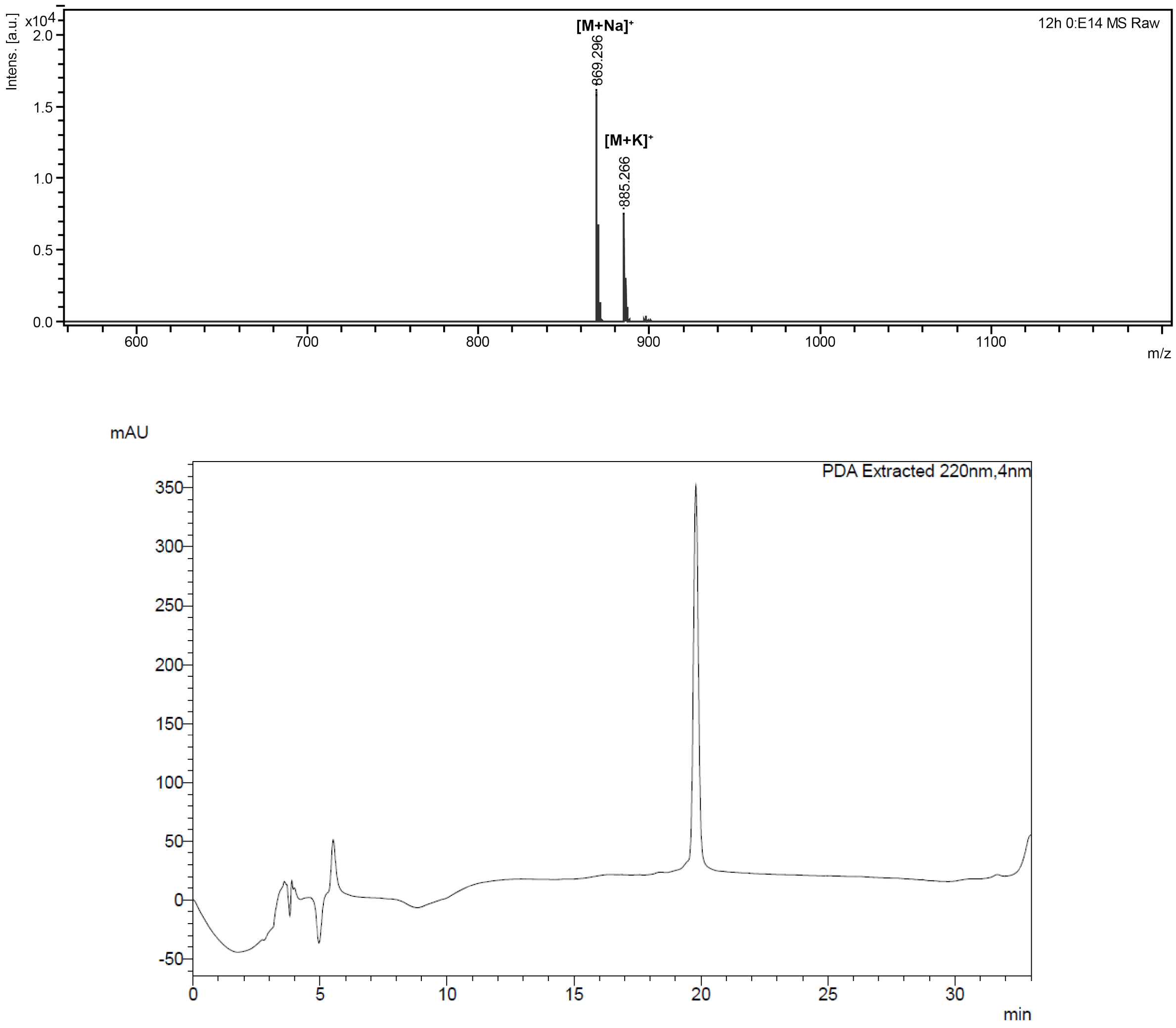

Supplementary Figure 56. Purity of macrocyclic peptides. ESI-MS (**a**) and HPLC purity profile (**b**) of peptide **12h.**

a

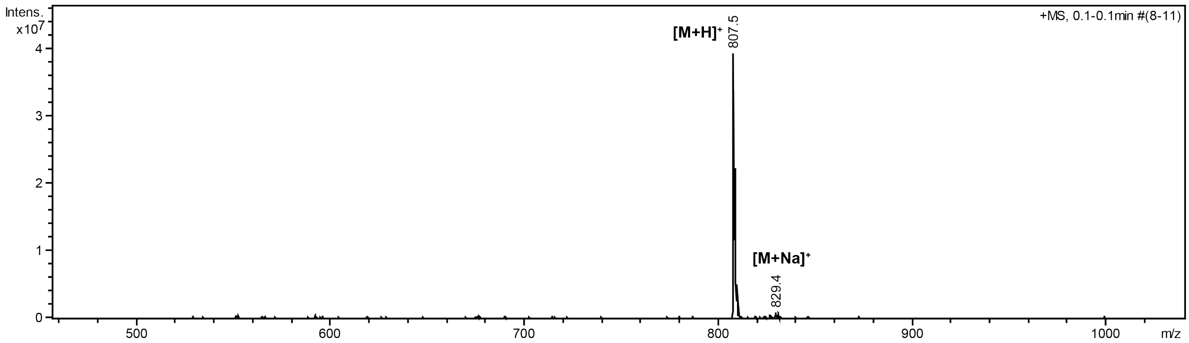

b

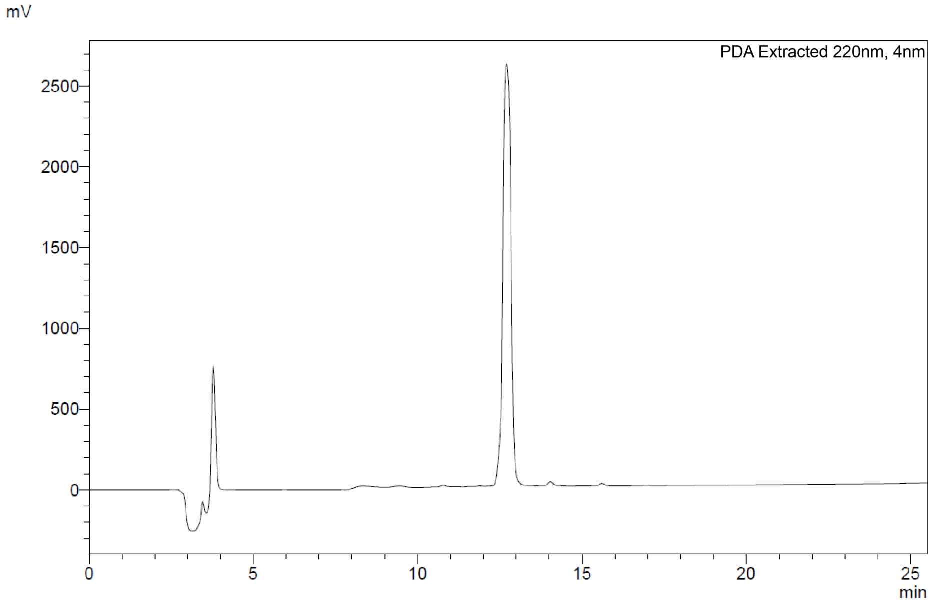

Supplementary Figure 57. Purity of macrocyclic peptides. ESI-MS (**a**) and HPLC purity profile (**b**) of peptide **13**.

a

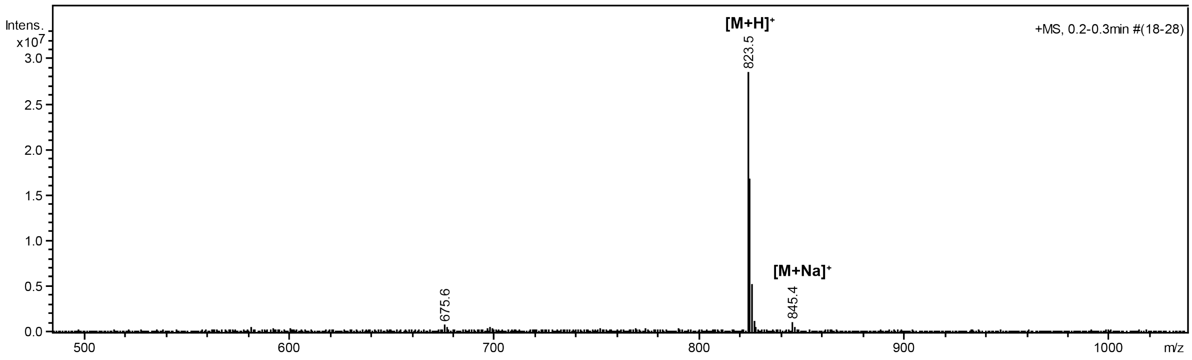

b

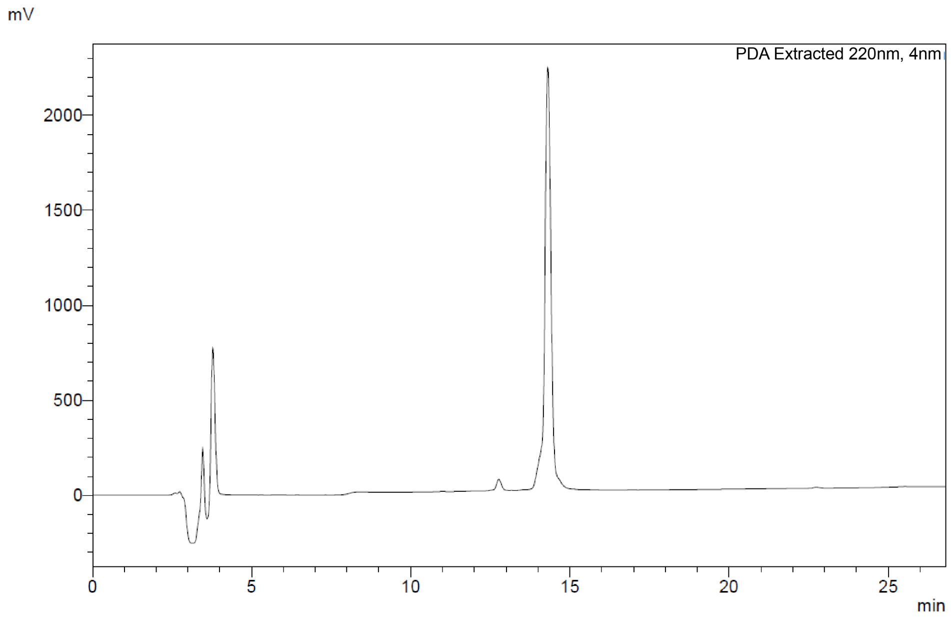

Supplementary Figure 58. Purity of macrocyclic peptides. ESI-MS (**a**) and HPLC purity profile (**b**) of peptide **13a**.

a

b

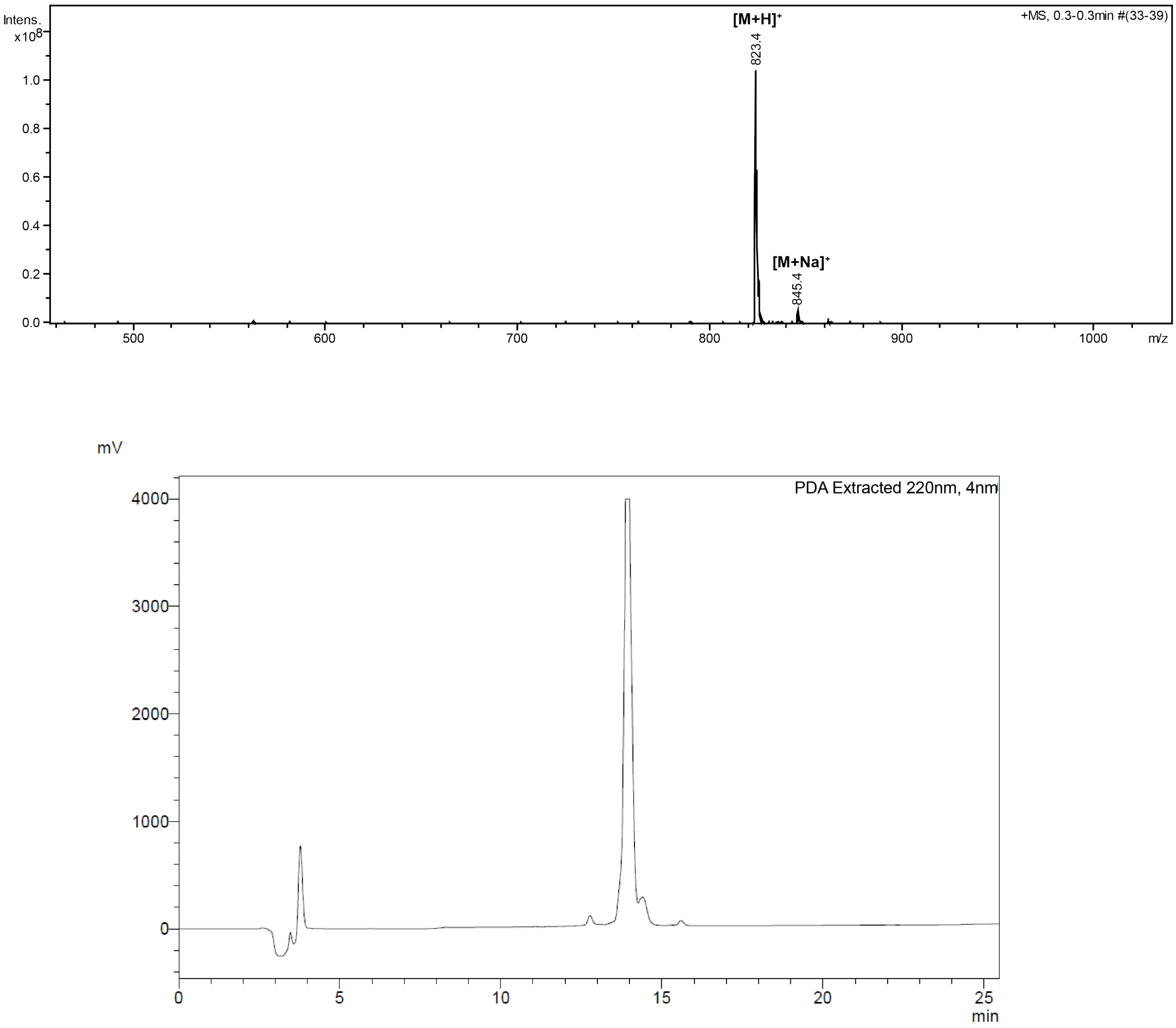

Supplementary Figure 59. Purity of macrocyclic peptides. ESI-MS (**a**) and HPLC purity profile (**b**) of peptide **13b.**

a

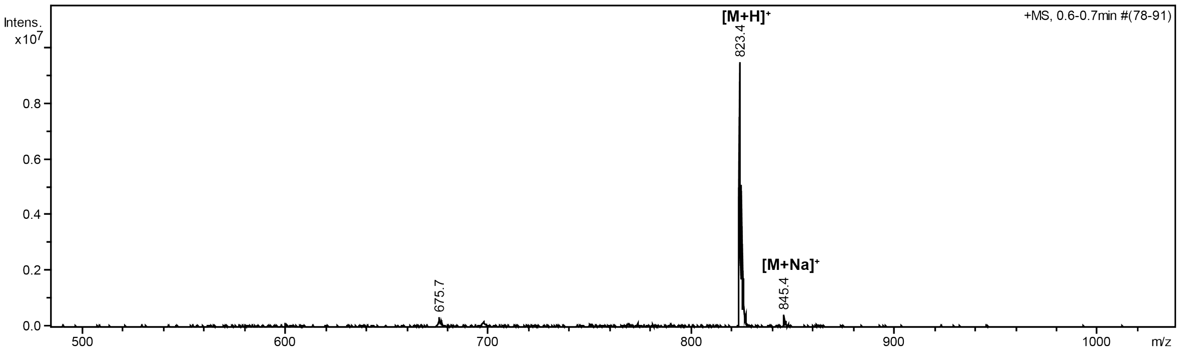

b

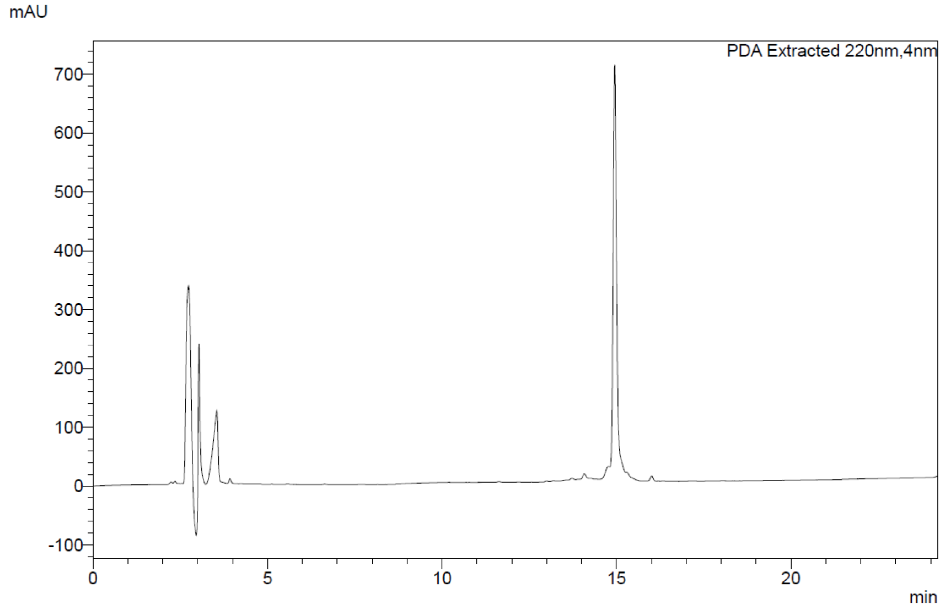

Supplementary Figure 60. Purity of macrocyclic peptides. ESI-MS (**a**) and HPLC purity profile (**b**) of peptide **13c**.

a

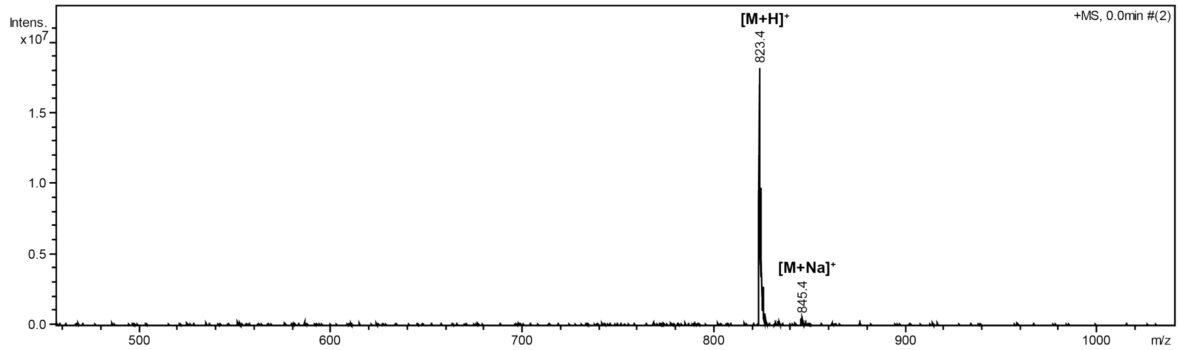

b

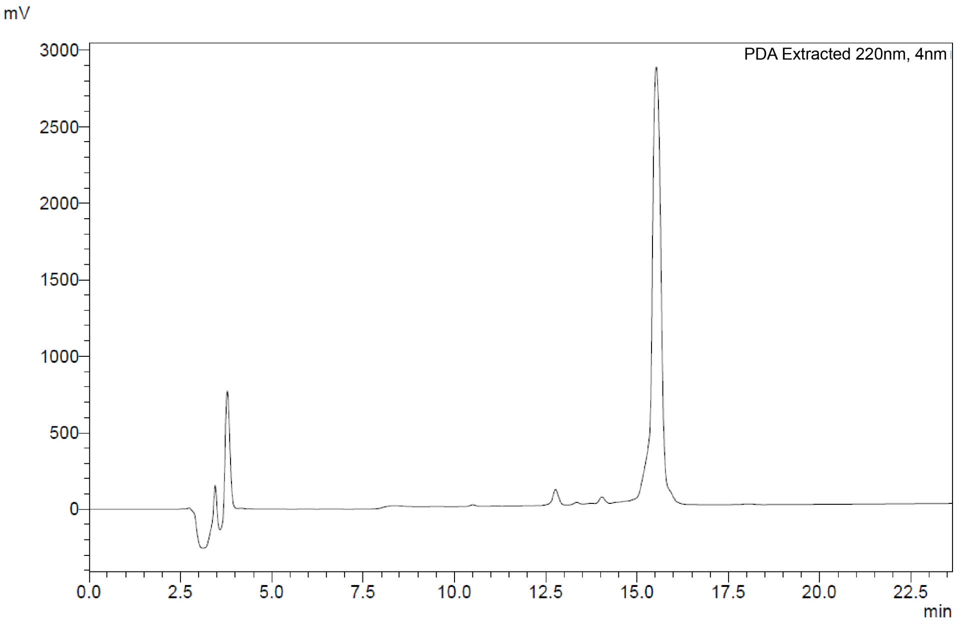

Supplementary Figure 61. Purity of macrocyclic peptides. ESI-MS (**a**) and HPLC purity profile (**b**) of peptide **13d**.

a

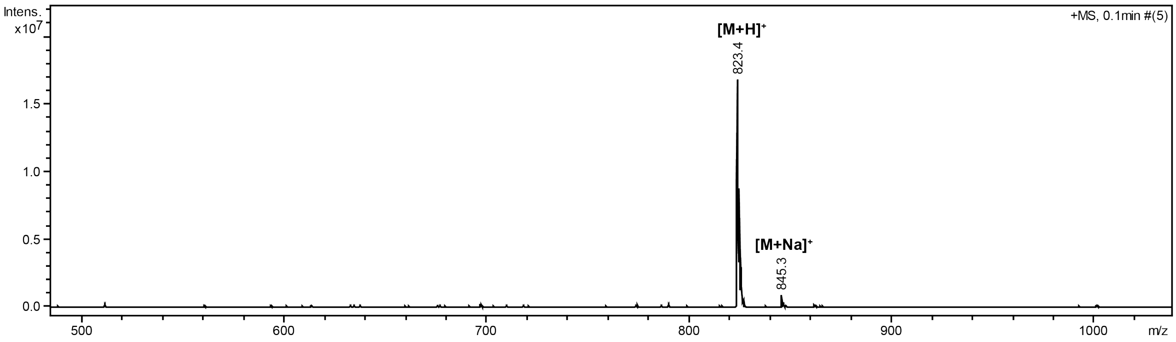

b

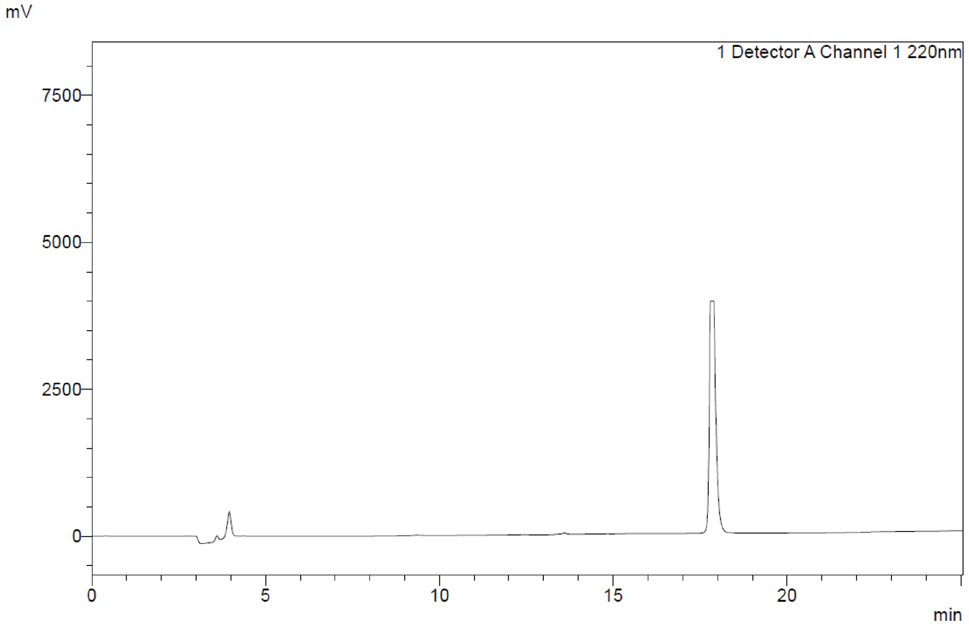

Supplementary Figure 62. Purity of macrocyclic peptides. ESI-MS (**a**) and HPLC purity profile (**b**) of peptide **13e**.

a

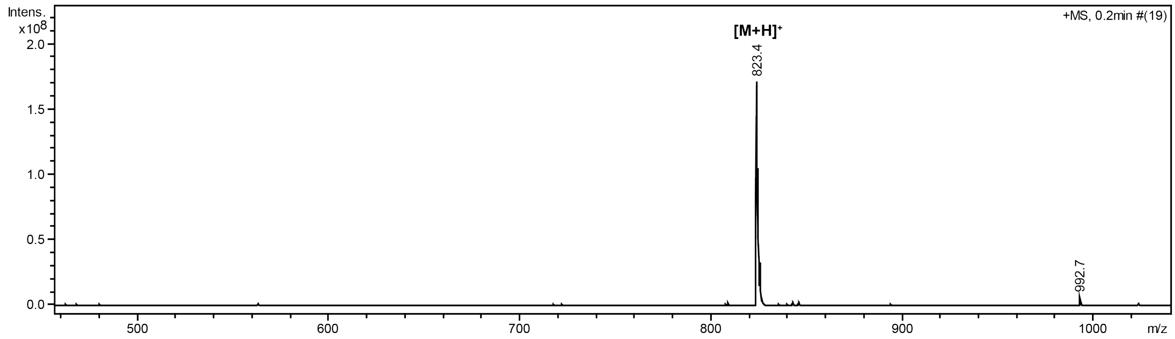

b

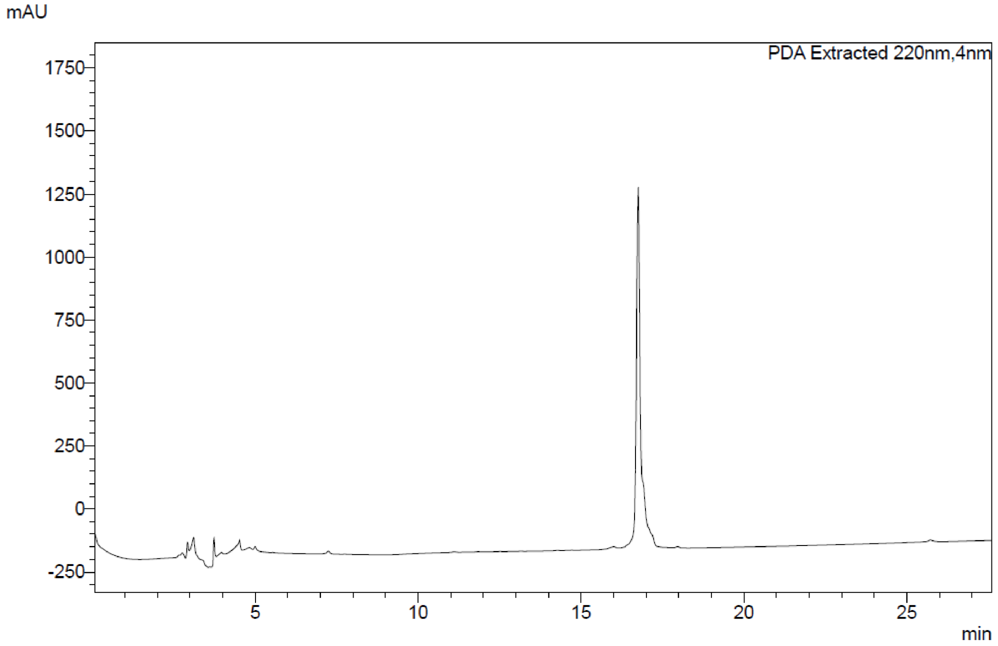

Supplementary Figure 63. Purity of macrocyclic peptides. ESI-MS (**a**) and HPLC purity profile (**b**) of peptide **13f**.

a

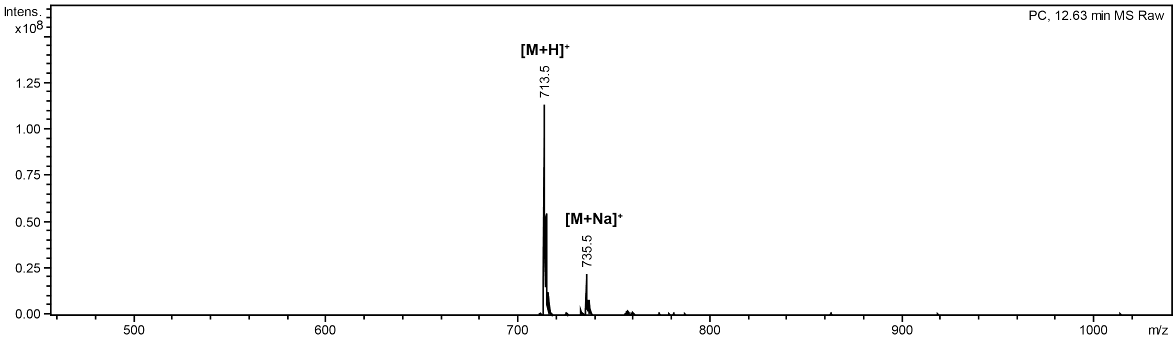

b

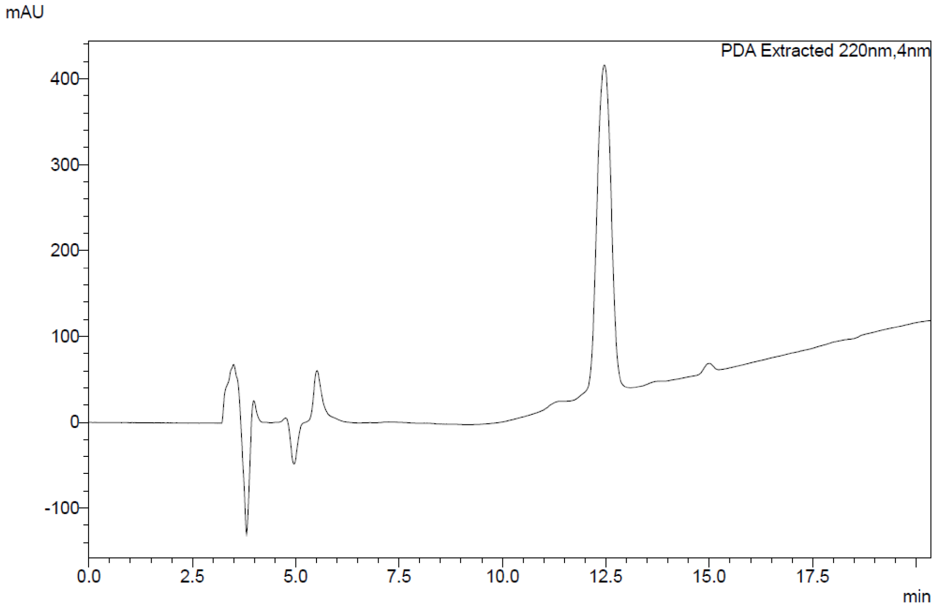

Supplementary Figure 64. Purity of macrocyclic peptides. ESI-MS (**a**) and HPLC purity profile (**b**) of peptide control (**PC**).

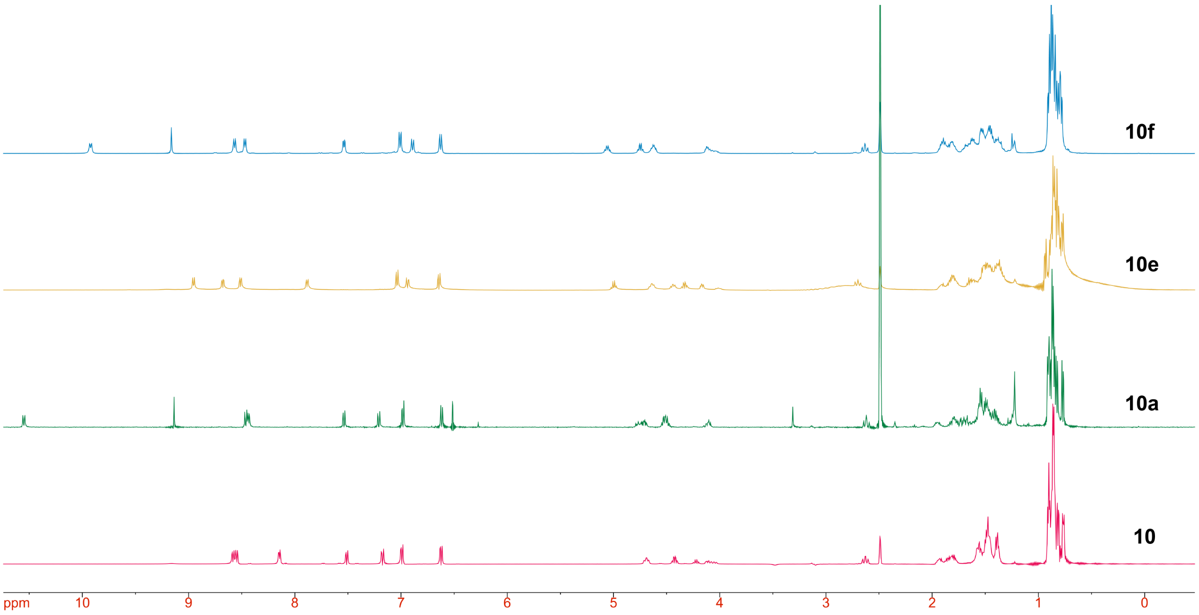

Supplementary Figure 65. Proton (^{1}H) NMR spectra. ^{1}H NMR overlay of **10**, **10a**, **10e**, and **10f** in DMSO-d_6.

a

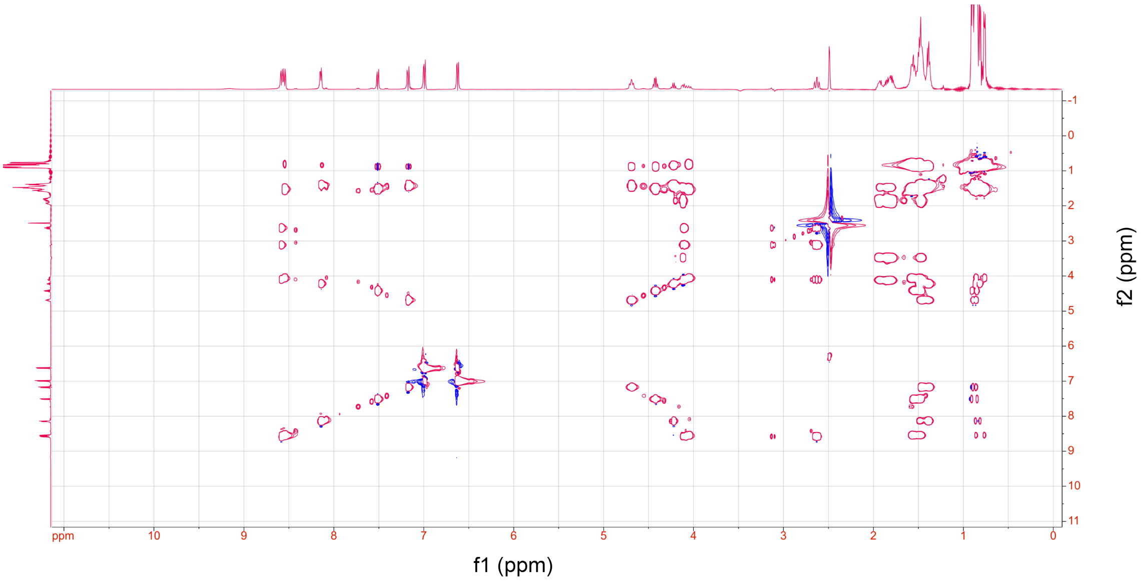

b

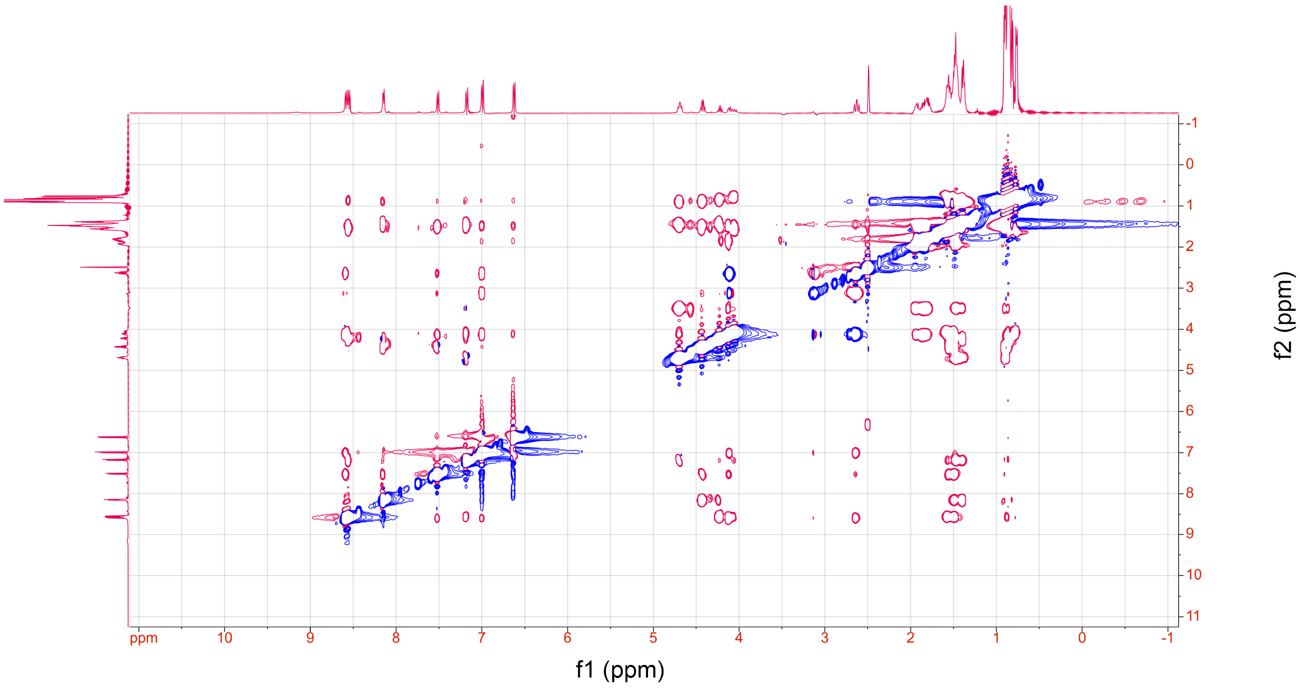

Supplementary Figure 66. Two-dimensional ^{1}H NMR spectra in DMSO-*d_6*. TOCSY (**a**) and ROESY (**b**) spectra of **10**.

a

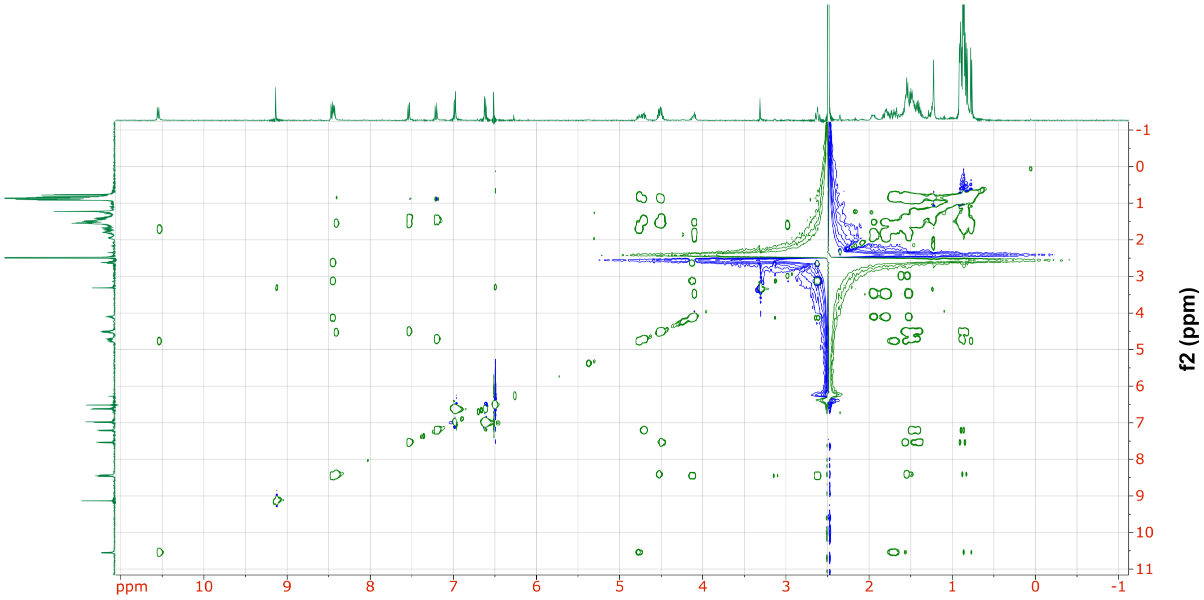

b

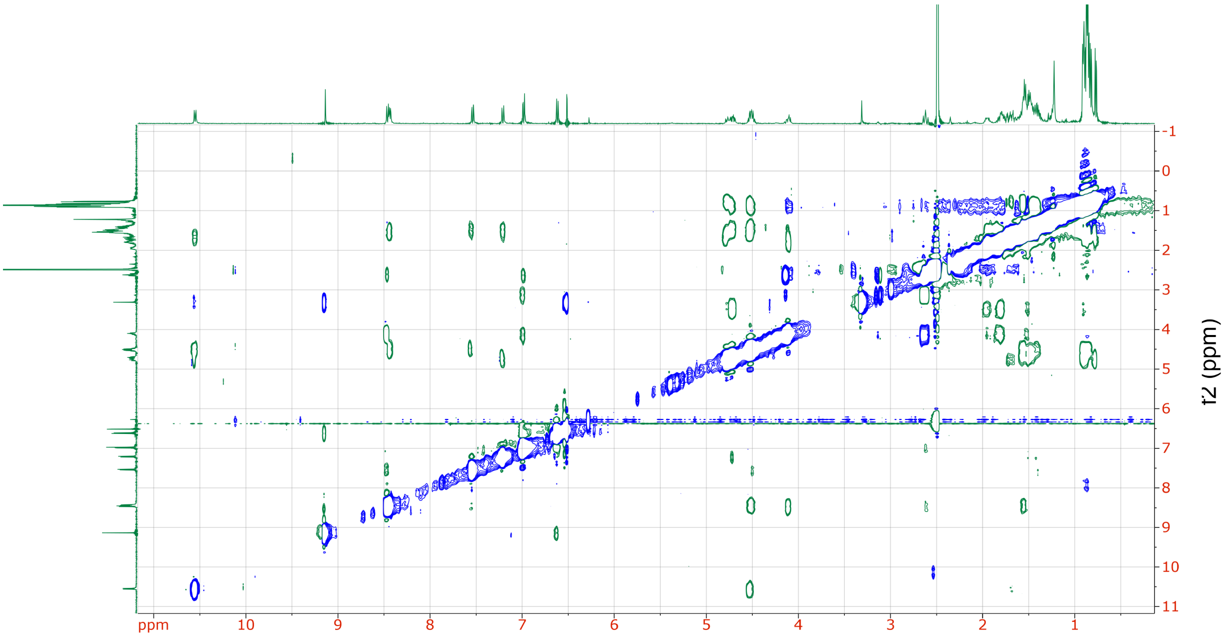

Supplementary Figure 67. Two-dimensional ^{1}H NMR spectra in DMSO-d_6. TOCSY (**a**) and ROESY (**b**) spectra of **10a**.

a

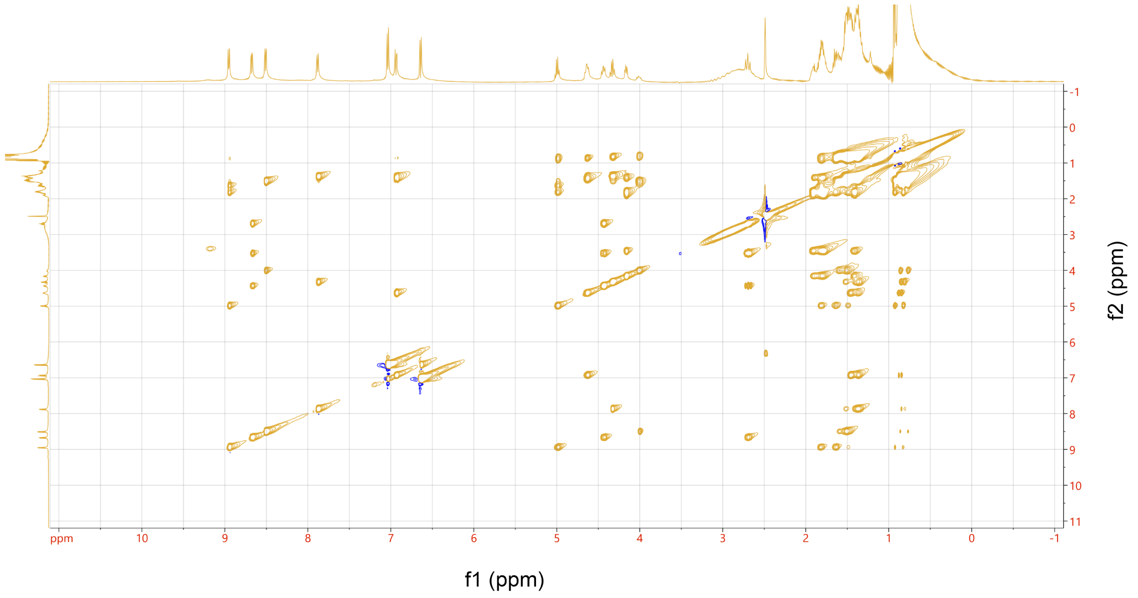

b

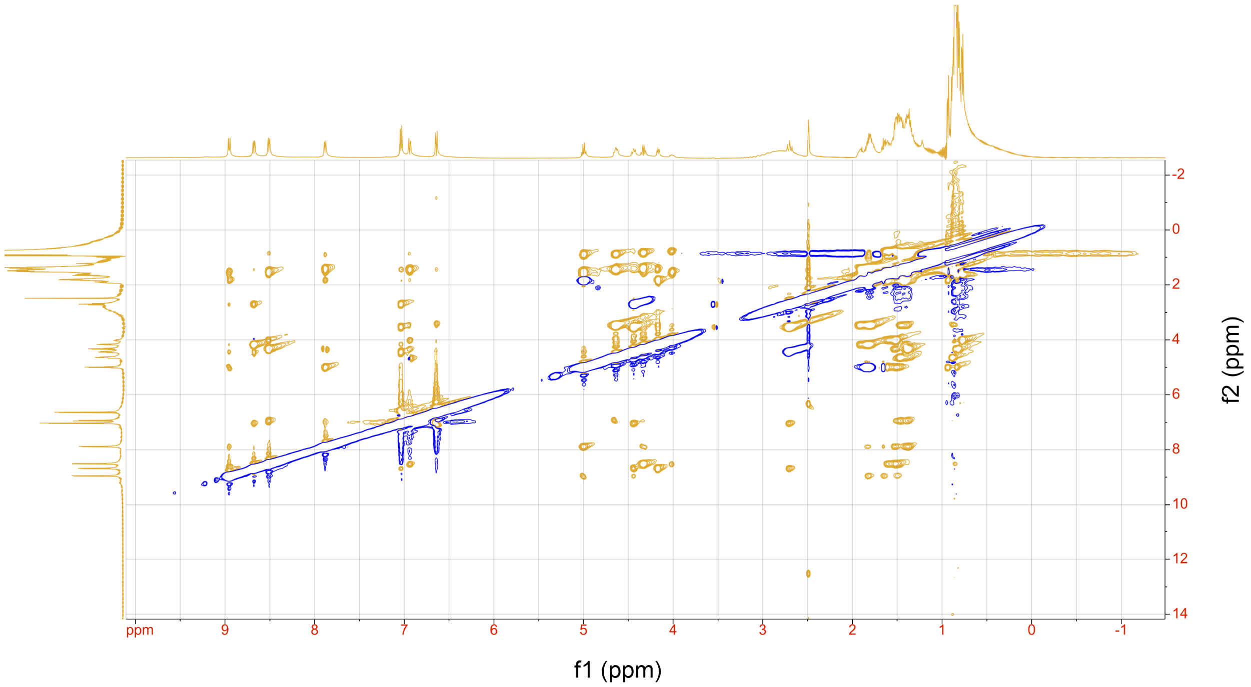

Supplementary Figure 68. Two-dimensional ^{1}H NMR spectra in DMSO-*d*$_6$. TOCSY (**a**) and ROESY (**b**) spectra of **10e**.

a

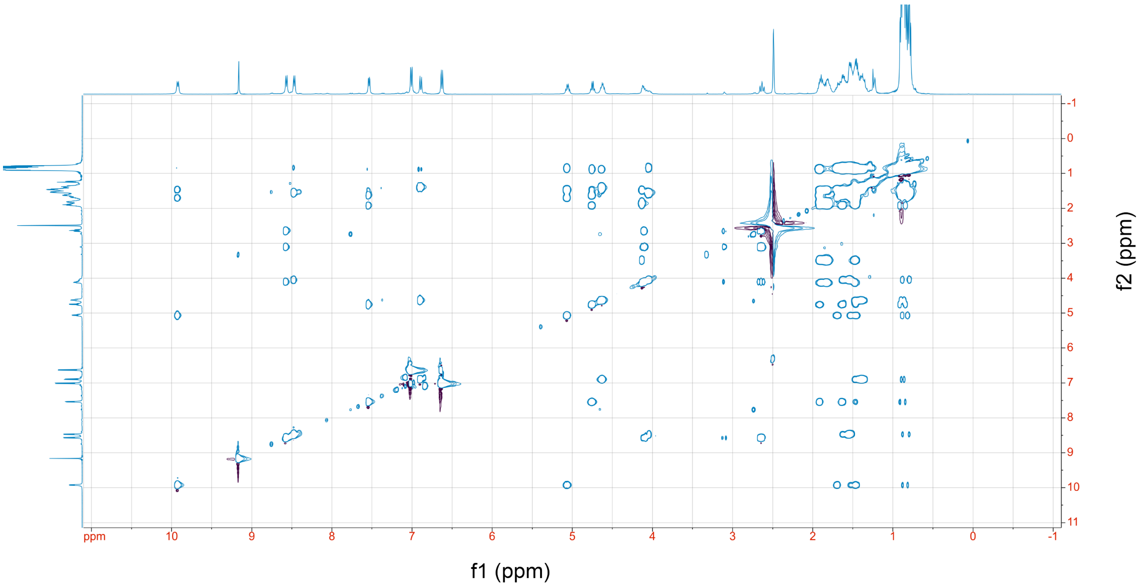

b

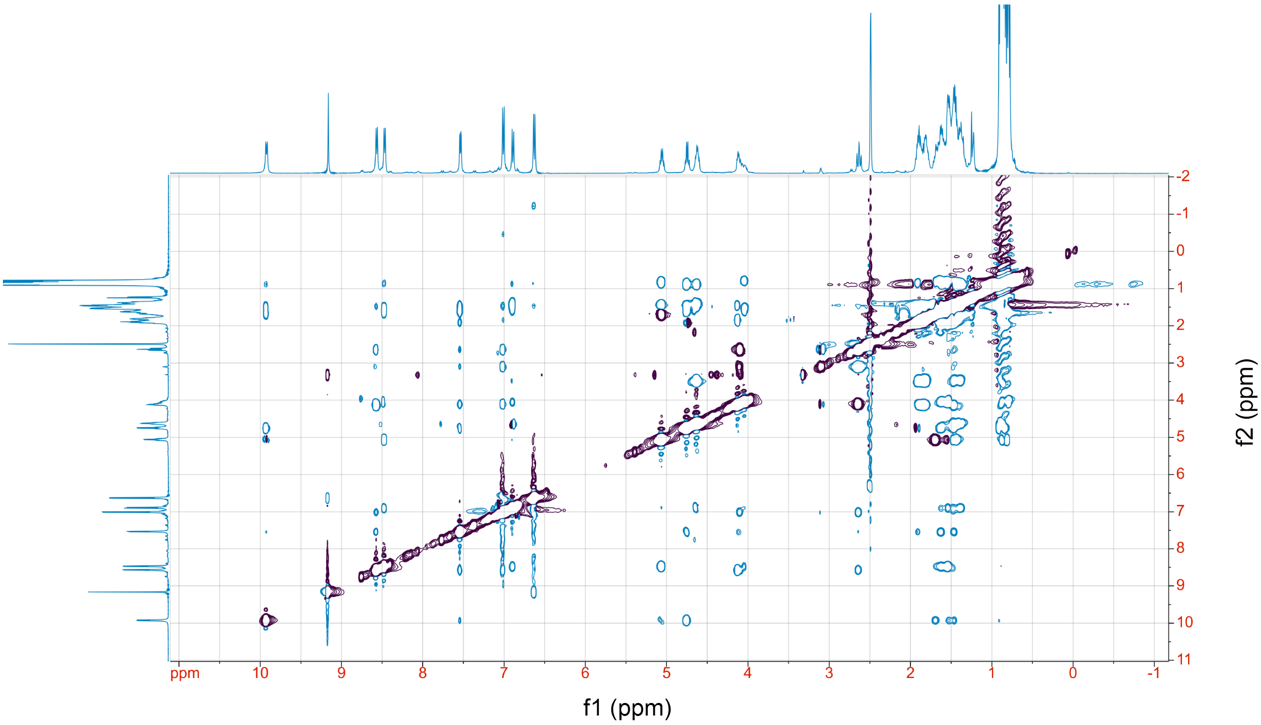

Supplementary Figure 69. Two-dimensional ^{1}H NMR spectra in DMSO-*d*$_6$. TOCSY (**a**) and ROESY (**b**) spectra of **10f**.

Supplementary Table 8. Absolute proton chemical shifts of **10, 10a, 10e** and **10f** in DMSO-d_6.

		**10**	**10a**	**10e**	**10f**
**D-Leu1**	**HN**	8.15	8.40	7.86	9.92
	**HA**	4.23	4.55	4.31	4.95
	**HB**	1.47	1.49	1.44	1.66
	**HG**	1.39	1.40	1.38	1.45
	**HD**	0.81/0.87	0.82/0.89	0.82/0.87	0.81/0.89
**Leu2**	**HN**	8.55	10.53	8.94	8.46
	**HA**	4.08	4.81	4.99	4.52
	**HB**	1.59	1.68	1.56	1.62
	**HG**	1.40	1.44	1.44	1.54
	**HD**	0.85/0.89	0.76/0.86	0.84/0.92	0.80/0.89
**Leu3**	**HN**	7.18	7.18	6.92	6.89
	**HA**	4.70	4.72	4.62	4.48
	**HB**	1.55	1.50	1.47	1.42
	**HG**	1.42	1.44	1.36	1.39
	**HD**	0.85/0.92	0.82/0.91	0.88/0.94	0.81/0.89
**D-Pro4**	**HA**	4.18	4.09	4.17	4.13
	**HB**	1.88	1.89	1.84	1.85
	**HG**	1.45	1.51	1.42	1.46
	**HD**	3.50	3.49	3.47	3.46
**Tyr5**	**HN**	8.60	8.44	8.66	8.57
	**HA**	4.12	4.13	4.44	4.82
	**HB**	2.63/3.13	2.62/3.12	2.69/3.13	2.71
**Leu6**	**HN**	7.51	7.53	8.47	7.53
	**HA**	4.43	4.51	4.01	4.58
	**HB**	1.60	1.57	1.61	1.53
	**HG**	1.47	1.42	1.47	1.43
	**HD**	0.82/0.90	0.83/0.89	0.82/0.90	0.88/0.95

Supplementary Table 9. Coupling constant and dihedral angle determined from ^{1}H NMR in DMSO-d_6.

**Residues**	**10**		**10a**		**10e**		**10f**	
	$^3J_{NH}$	$\theta_{H\text{-}N\text{-}C\alpha\text{-}H\alpha}$	$^3J_{NH}$	$\theta_{H\text{-}N\text{-}C\alpha\text{-}H\alpha}$	$^3J_{NH}$	$\theta_{H\text{-}N\text{-}C\alpha\text{-}H\alpha}$	$^3J_{NH}$	$\theta_{H\text{-}N\text{-}C\alpha\text{-}H\alpha}$
**D-Leu1**	6.5	138.5	8.2	148.7	8.1	148	7.7	145.6
**Leu2**	8.2	148.7	8.2	148.7	8.2	148.7	7.8	146.2
**Leu3**	9.5	158	8.2	148.7	9.5	158	9	154.1
**Tyr5**	8.1	148	5.7	134	5.5	132.9	7.7	145.6
**Leu6**	8.1	148	8.6	151.3	8	147.4	7.8	146.2

Supplementary Table 10. NOEs considered for the structure calculation of **10** in DMSO-d_6 and their violations.

10

Interactions	Lower limit	Upper limit	NOE distance	Violations
Leu1HA-HD1	2.25	2.75	2.99	0.24
Leu1HA-Leu2HN	1.85	2.26	2.28	0.02
Leu1HB1-HA	2.27	2.77	2.60	0.00
Leu1HB1-HD1	1.67	2.04	2.73	0.69
Leu1HN-HB2	2.04	2.50	2.26	0.00
Leu1HN-Leu3HN	3.82	4.66	4.62	0.00
Leu1HN-Leu6HA	2.91	355	3.56	0.01
Leu3HN-Leu2HA	2.31	2.83	3.03	0.20
Leu2HB1-HA	1.65	2.01	2.41	0.40
Leu2HB1-HD1	1.94	2.38	2.86	0.49
Leu2HB1-HN	2.01	2.45	2.60	0.15
Leu2HA-HD1	1.94	2.38	2.79	0.41
Leu2HN-HA	2.38	2.90	2.89	0.00
Leu3HA-HB1	1.65	2.01	2.75	0.73
Leu3HN-HA	1.98	2.42	2.52	0.10
Leu3HB1-HD1	1.75	2.13	2.80	0.66
Leu3HA-HD1	2.16	2.64	3.24	0.60
Leu3HG-HA	1.90	2.32	2.35	0.03
Leu3HN-HG1	2.11	2.57	2.52	0.00
Pro4HA-HD1	2.55	3.11	3.35	0.23
Pro4HA-Leu6HN	2.72	3.32	3.39	0.07
Pro4HD1-HB1	1.95	2.39	2.77	0.39
Pro4HD2-Leu3HA	1.59	1.95	2.22	0.27
Pro4HD2-Leu3HN	2.90	3.54	4.15	0.61
Pro4HD1-HG1	1.86	2.28	2.38	0.10
Tyr5HA-HD1	2.16	2.64	2.67	0.03
Tyr5HA-Leu6HN	2.86	3.50	3.30	0.00
Tyr5HA-HB1	2.22	2.72	3.05	0.33
Tyr5HB2-HB1	1.67	2.04	1.73	0.00
Tyr5HN-HB1	2.35	2.87	2.64	0.00
Tyr5HA-HB2	2.24	2.74	2.48	0.00
Tyr5HD2-HB1	2.21	2.71	2.41	0.00
Tyr5HD1-HB2	2.16	2.64	2.39	0.00
Tyr5HB1-HB2	1.85	2.26	1.73	-0.11
Tyr5HN-Pro4HA	1.84	2.24	2.08	0.00
Tyr5HB1-Leu6HN	3.54	4.32	4.22	0.00
Leu6HA-HN	2.04	2.50	2.90	0.41
Leu6HB1-HD1	2.09	2.55	2.83	0.28
Leu6HN-HB1	2.32	2.84	2.56	0.00
Leu6HA-Pro4HA	3.18	3.89	4.12	0.23

Supplementary Table 11. NOEs considered for the structure calculation of **10a** in DMSO-d_6 and their violations.

10a

Interactions	Lower limit	Upper limit	NOE distance	Violations
Leu1HB1-HA	2.27	2.77	2.61	0.00
Leu1HN-HB1	2.04	2.50	3.32	0.83
Leu1HA-HD1	2.25	2.75	3.00	0.25
Leu1HB1-HD1	1.42	1.74	2.73	1.00
Leu1HA-Leu2HN	1.85	2.26	2.25	0.00
Leu2HB1-HA	1.65	2.01	2.44	0.42
Leu2HN-HA	2.38	2.90	2.90	0.00
Leu2HB1-HN	2.01	2.45	2.61	0.15
Leu2HA-HD1	1.94	2.38	2.81	0.44
Leu2HB1-HD1	1.94	2.38	2.85	0.48
Leu3HN-Leu2HA	2.31	2.83	3.38	0.55
Leu3HA-HB1	1.65	2.01	2.40	0.38
Leu3HN-HA	1.98	2.42	2.89	0.47
Leu3HA-HD1	2.16	2.64	2.94	0.30
Leu3HG-HA	1.90	2.32	2.78	0.46
Leu3HN-HG	2.11	2.57	4.09	1.52
Pro4HA-HD2	2.55	3.11	4.09	0.98
Pro4HD1-HB1	1.95	2.39	2.78	0.40
Pro4HA-Leu6HN	2.72	3.32	3.47	0.15
Pro4HD1-Leu3HA	1.59	1.95	2.60	0.65
Pro4HD1-Leu3HN	2.90	3.54	4.82	1.28
Pro4HD1-HG1	1.86	2.28	2.37	0.10
Tyr5HA-HD1	2.16	2.64	2.62	0.00
Tyr5HA-Leu6HN	2.86	3.50	3.42	0.00
Tyr5HA-HB1	2.22	2.72	2.83	0.11
Tyr5HB2-HB1	1.67	2.04	1.73	0.00
Tyr5HN-HB1	2.35	2.87	2.59	0.00
Tyr5HA-HB2	2.24	2.74	2.71	0.00
Tyr5HD1-HB1	2.21	2.71	3.08	0.37
Tyr5HD1-HB2	2.16	2.64	2.88	0.24
Tyr5HN-Pro4HA	1.84	2.24	2.10	0.00
Leu6HA-HN	2.04	2.50	2.88	0.39
Leu6HB1-HD1	2.09	2.55	2.84	0.28
Leu6HN-HB1	2.32	2.84	2.54	0.00
Leu6HA-Leu3HA	3.86	4.72	7.34	2.62

Supplementary Table 12. NOEs considered for the structure calculation of **10e** in DMSO-d_6 and their violations.

10e

Interactions	Lower limit	Upper limit	NOE distance	Violations
Leu1HB1-HA	2.27	2.77	2.68	0.00
Leu1HN-HB1	2.49	3.05	3.32	0.28
Leu1HA-HD1	2.45	2.99	3.60	0.60
Leu1HB1-HD1	1.98	2.42	3.00	0.58
Leu1HA-Leu2HN	1.85	2.26	2.15	0.00
Leu1HN-Leu3HN	3.64	4.44	4.63	0.19
Leu2HB2-HA	1.65	2.01	2.54	0.52
Leu2HN-HA	2.38	2.90	2.98	0.08
Leu2HB2-HN	2.41	2.95	3.64	0.69
Leu2HB1-HD1	1.94	2.38	2.92	0.54
Leu3HN-Leu2HA	2.31	2.83	3.30	0.47
Leu3HA-HB2	1.65	2.01	2.63	0.62
Leu3HN-HA	1.98	2.42	2.96	0.54
Leu3HB1-HD1	1.75	2.13	2.94	0.81
Leu3HA-HD2	2.16	2.64	3.33	0.69
Leu3HG-HA	1.90	2.32	2.98	0.66
Leu3HN-HG	2.11	2.57	3.38	0.81
Pro4HA-HD1	2.55	3.11	3.60	0.48
Pro4HD1-HB1	2.71	3.31	3.86	0.55
Pro4HA-Leu6HN	2.72	3.32	3.63	0.31
Pro4HD2-Leu3HA	1.98	2.42	2.39	0.00
Pro4HD1-HG1	1.86	2.28	2.34	0.06
Tyr5HA-HD1	2.16	2.64	3.44	0.80
Tyr5HA-Leu6HN	2.86	3.50	2.91	0.00
Tyr5HA-HB1	2.22	2.72	3.06	0.34
Tyr5HB2-HB1	1.67	2.04	1.74	0.00
Tyr5HN-HB1	2.35	2.87	2.78	0.00
Tyr5HA-HB2	2.24	2.74	2.54	0.00
Tyr5HD2-HB1	2.21	2.71	3.02	0.31
Tyr5HD1-HB2	2.16	2.64	3.05	0.41
Tyr5HN-Pro4HA	1.84	2.24	2.09	0.00
Leu6HA-HN	2.04	2.50	2.92	0.42
Leu6HB1-HD1	2.09	2.55	2.93	0.38
Leu6HN-HB1	2.32	2.84	2.88	0.04

Supplementary Table 13. NOEs considered for the structure calculation of **10f** in DMSO-d_6 and their violations.

10f

Interactions	Lower limit	Upper limit	NOE distance	Violations
Leu1HB1-HA	2.27	2.77	2.64	0.00
Leu1HN-HB1	2.49	3.05	3.32	0.27
Leu1HA-HD1	2.45	2.99	3.01	0.02
Leu1HB1-HD1	1.98	2.42	2.73	0.31
Leu1HA-Leu2HN	1.85	2.26	2.21	0.00
Leu2HB2-HA	1.65	2.01	3.04	1.03
Leu2HN-HA	2.38	2.90	2.88	0.00
Leu2HB2-HN	2.41	2.95	2.53	0.00
Leu2HB1-HD1	1.94	2.38	2.86	0.48
Leu3HN-Leu2HA	2.31	2.83	3.44	0.62
Leu3HA-HB2	1.65	2.01	2.82	0.81
Leu3HN-HA	1.98	2.42	2.76	0.34
Leu3HB1-HD1	1.75	2.13	2.78	0.65
Leu3HA-HD2	2.16	2.64	4.40	1.76
Leu3HG-HA	1.90	2.32	2.45	0.13
Leu3HN-HG	2.11	2.57	3.69	1.11
Pro4HA-HD1	2.55	3.11	3.33	0.22
Pro4HD1-HB1	2.71	3.31	2.80	0.00
Pro4HA-Leu6HN	2.72	3.32	3.49	0.17
Pro4HD2-Leu3HA	1.98	2.42	2.18	0.00
Pro4HD2-Leu3HN	2.90	3.54	4.40	0.86
Pro4HD1-HG1	1.86	2.28	2.38	0.10
Tyr5HA-HD1	2.16	2.64	2.63	0.00
Tyr5HA-Leu6HN	2.86	3.50	3.03	0.00
Tyr5HA-HB1	2.22	2.72	3.05	0.33
Tyr5HB2-HB1	1.67	2.04	1.73	0.00
Tyr5HN-HB1	2.35	2.87	2.70	0.00
Tyr5HA-HB2	2.24	2.74	2.49	0.00
Tyr5HD2-HB1	2.21	2.71	2.38	0.00
Tyr5HD1-HB2	2.16	2.64	2.42	0.00
Tyr5HN-Pro4HA	1.84	2.24	2.09	0.00
Leu6HA-HN	2.04	2.50	2.94	0.44
Leu6HB1-HD1	2.09	2.55	2.80	0.25
Leu6HN-HB1	2.32	2.84	2.74	0.00

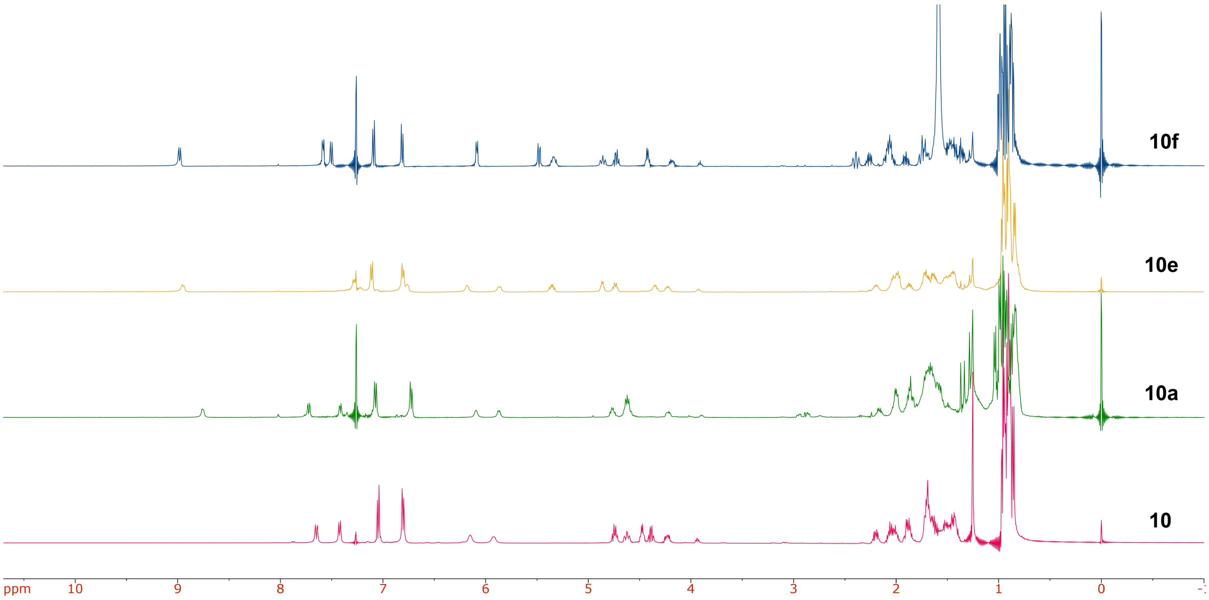

Supplementary Figure 70. Proton (^{1}H) NMR spectra in $CDCl_3$. ^{1}H NMR overlay of **10**, **10a**, **10e**, and **10f** in $CDCl_3$.

a

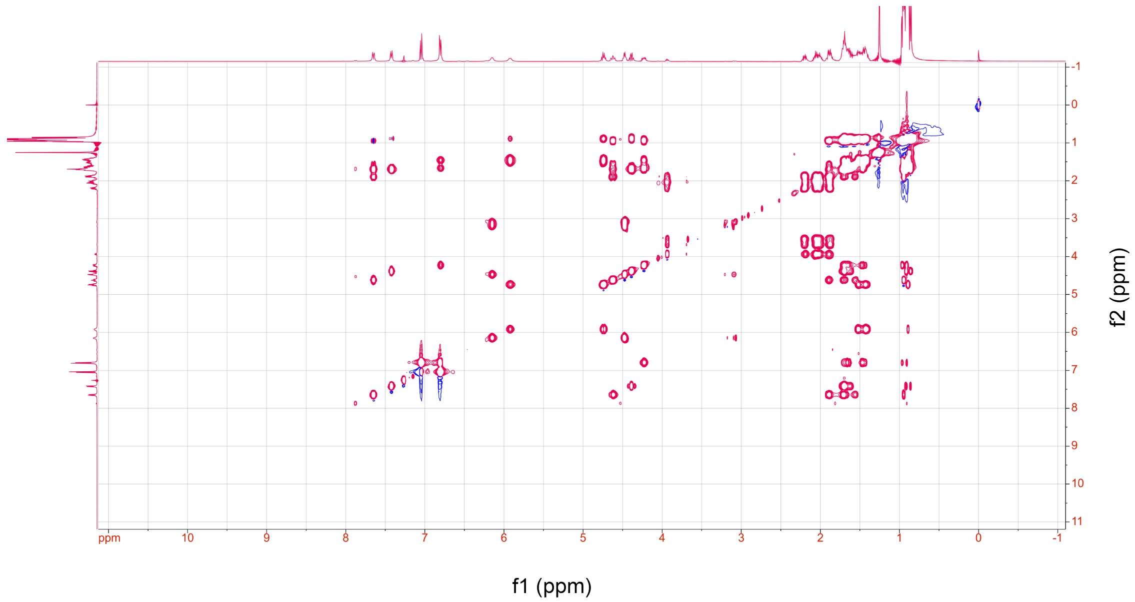

b

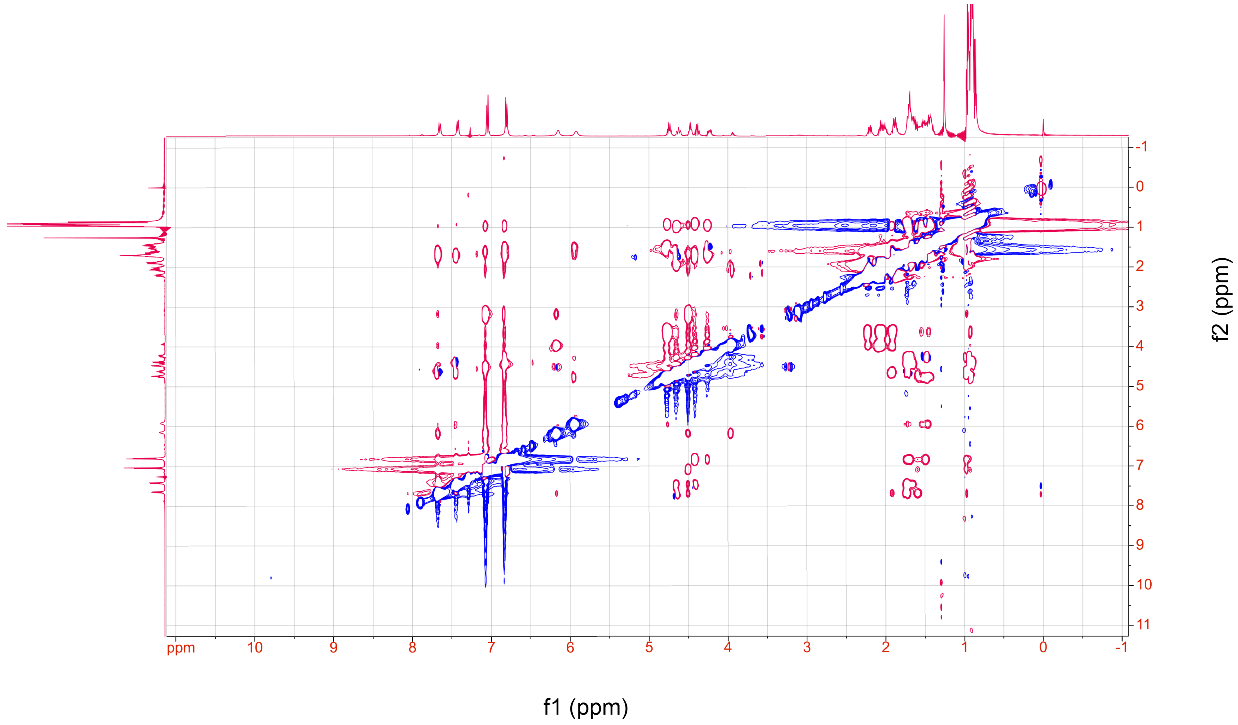

Supplementary Figure 71. Two-dimensional ^{1}H NMR spectra in $CDCl_3$. TOCSY (**a**) and ROESY (**b**) spectra of **10** in $CDCl_3$.

a

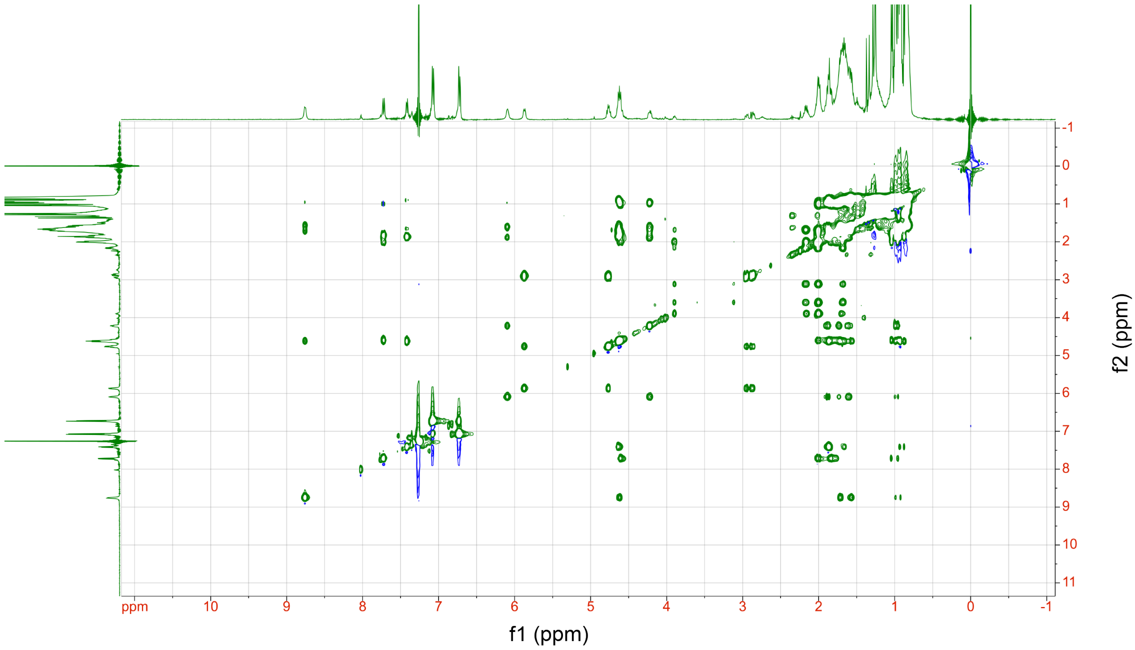

b

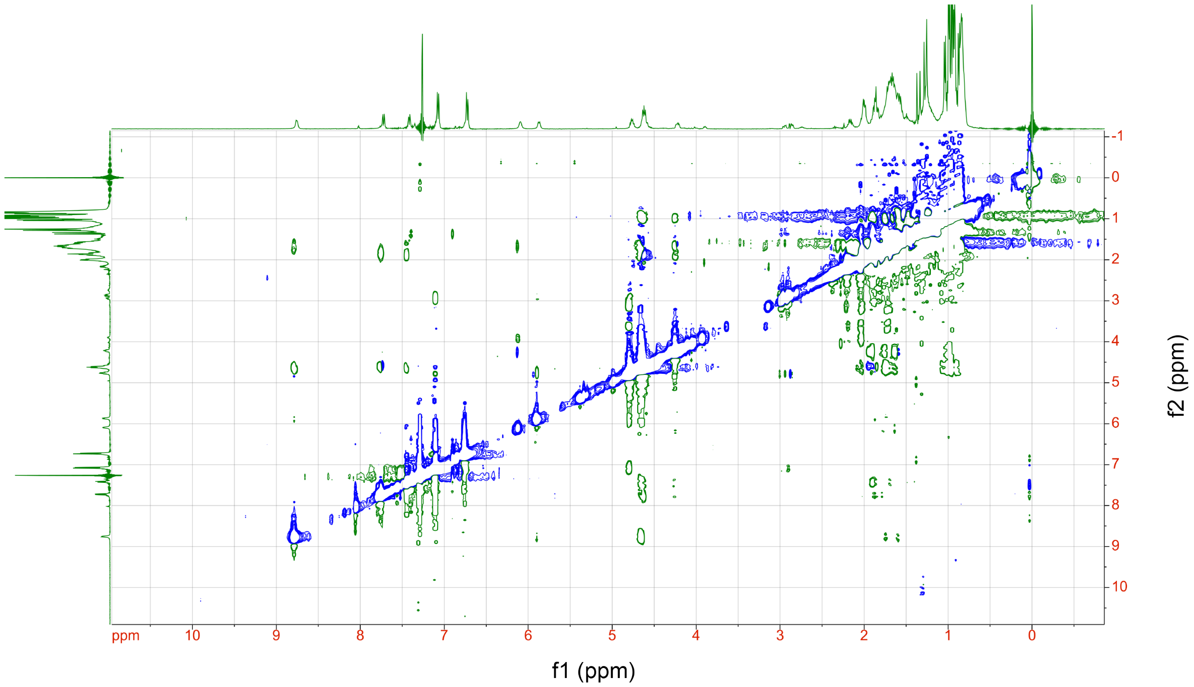

Supplementary Figure 72. Two-dimensional ^{1}H NMR spectra in $CDCl_3$. TOCSY (**a**) and ROESY (**b**) spectra of **10a** in $CDCl_3$.

a

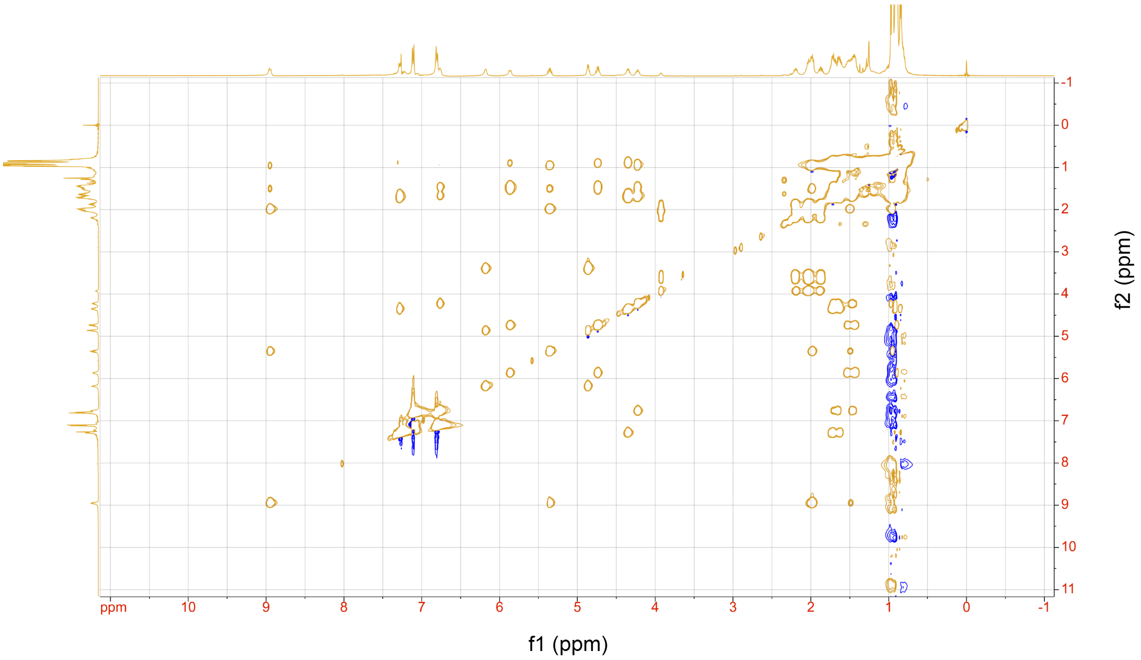

b

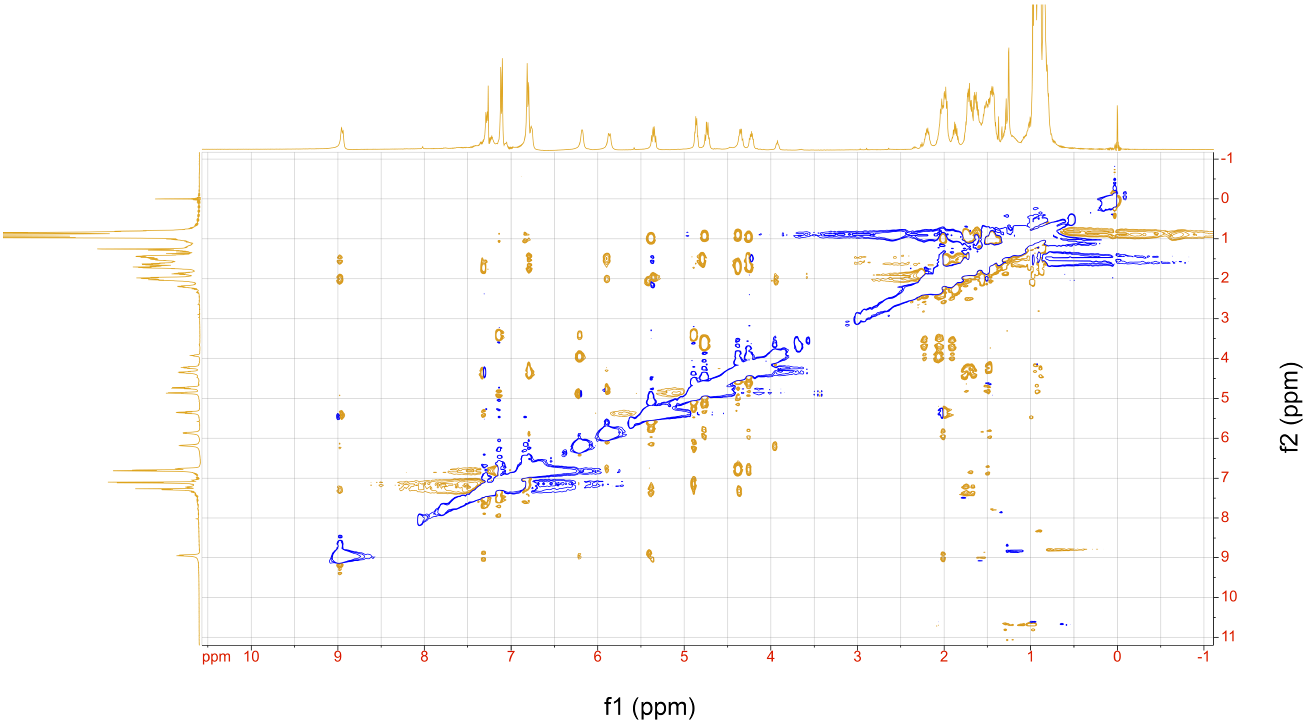

Supplementary Figure 73. Two-dimensional ^{1}H NMR spectra in $CDCl_3$. TOCSY (**a**) and ROESY (**b**) spectra of **10e** in $CDCl_3$.

a

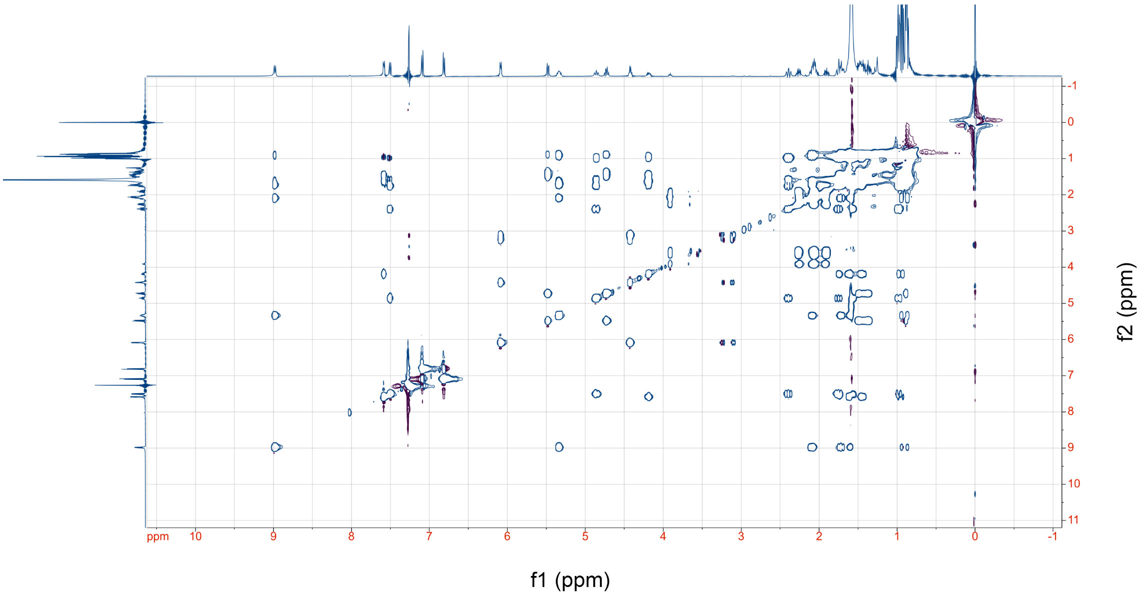

b

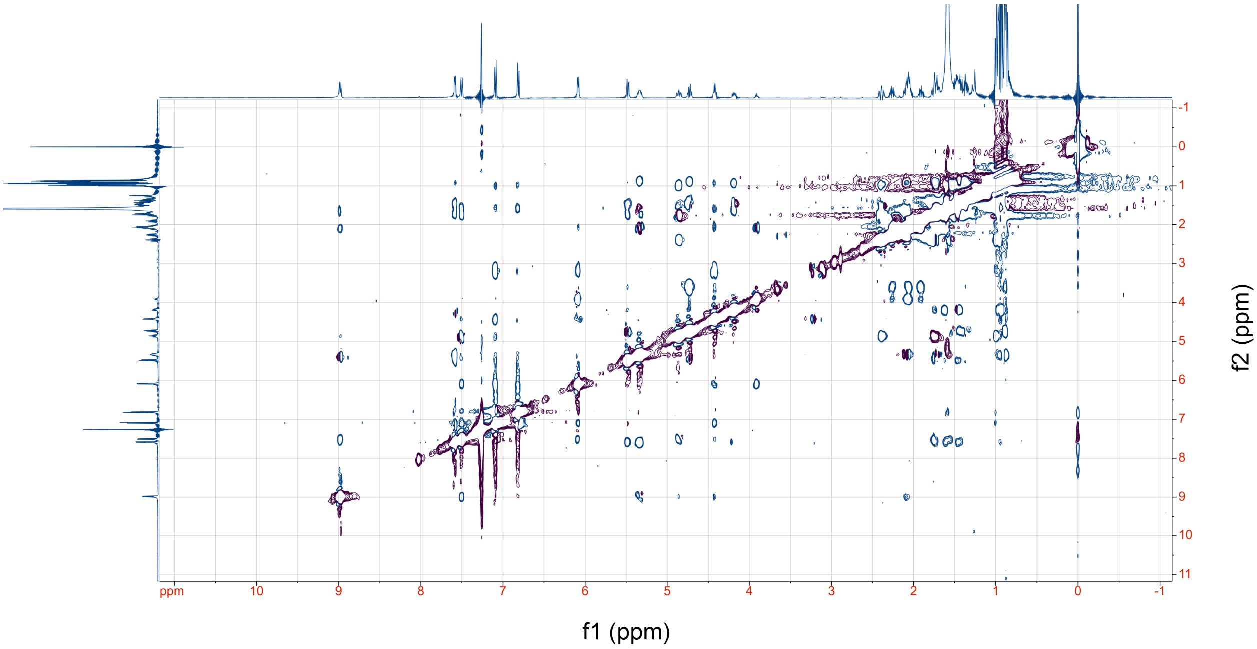

Supplementary Figure 74. Two-dimensional ^{1}H NMR spectra in $CDCl_3$. TOCSY (**a**) and ROESY (**b**) spectra of **10f** in $CDCl_3$.

Supplementary Table 14. Absolute proton chemical shifts of **10, 10a, 10e** and **10f** in $CDCl_3$.

		**10**	**10a**	**10e**	**10f**
**D-Leu1**	**HN**	7.45	7.41	7.29	8.95
	**HA**	4.38	4.64	4.40	5.33
	**HB**	1.76	1.91	1.75	1.76/2.09
	**HG**	1.58	1.64	1.59	1.59
	**HD**	0.89	0.90	0.91	0.92
**Leu2**	**HN**	6.83	8.75	6.74	7.57
	**HA**	4.34	4.62	4.23	4.16
	**HB**	1.69	1.71	1.67	1.62
	**HG**	1.48	1.55	1.43	1.43
	**HD**	0.93	0.96	0.96	0.94
**Leu3**	**HN**	5.95	6.08	5.89	5.48
	**HA**	4.79	4.22	4.76	4.77
	**HB**	1.53	1.88	1.56	1.52
	**HG**	1.43	1.61	1.41	1.37
	**HD**	0.92	0.96	0.92	0.89
**D-Pro4**	**HA**	3.99	3.90	3.96	3.93
	**HB**	2.07/2.18	2.00/2.16	2.02/2.15	2.06/2.23
	**HG**	1.89/1.99	1.78	1.87	1.93
	**HD**	3.55/3.70	3.63	3.62	3.57/3.67
**Tyr5**	**HN**	6.17	5.86	6.17	6.08
	**HA**	4.52	4.77	4.88	4.44
	**HB**	3.12	2.90	3.88	3.09/3.29
**Leu6**	**HN**	7.68	7.72	8.96	7.50
	**HA**	4.67	4.61	5.40	4.86
	**HB**	1.90	1.76/1.97	1.99	1.75/2.44
	**HG**	1.65	1.52	1.49	1.60
	**HD**	0.94	1.01	0.93	0.98

Supplementary Table 15: Coupling constant and dihedral angle determined from ^1H NMR in $CDCl_3$.

**Residues**	**10**		**10a**		**10e**		**10f**	
	$^3J_{NH}$	$\theta_{H\text{-}N\text{-}C\alpha\text{-}H\alpha}$	$^3J_{NH}$	$\theta_{H\text{-}N\text{-}C\alpha\text{-}H\alpha}$	$^3J_{NH}$	$\theta_{H\text{-}N\text{-}C\alpha\text{-}H\alpha}$	$^3J_{NH}$	$\theta_{H\text{-}N\text{-}C\alpha\text{-}H\alpha}$
**D-Leu1**	8	147.4	6.6	139.1	7.7	145.6	8.1	148
**Leu2**	8	147.4	7	141.4	5.8	134.6	8	147.4
**Leu3**	8.1	148	n.d.	n.d.	8.6	151.3	9.4	157.2
**Tyr5**	n.d.	n.d.	8	147.4	n.d.	n.d.	7.8	146.2
**Leu6**	9.4	157.2	8.1	148	7.7	145.6	8.1	1481

n.d.: could not be determined.

Supplementary Table 16. NOEs considered for the structure calculation of **10** in $CDCl_3$ and their violations.

10

Interactions	Lower limit	Upper limit	NOE distance	Violations
Leu1HA-HD1	2.25	2.75	3.49	0.74
Leu1HA-Leu2HN	1.85	2.26	2.20	0.00
Leu1HB1-HA	2.27	2.77	2.65	0.00
Leu1HB1-HD1	1.67	2.04	3.00	0.96
Leu1HN-HB2	2.04	2.50	2.54	0.04
Leu3HN-Leu2HA	2.31	2.83	3.31	0.48
Leu2HB1-HA	1.65	2.01	3.04	1.03
Leu2HB1-HD1	1.94	2.38	2.95	0.57
Leu2HB1-HN	2.01	2.45	2.70	0.25
Leu2HN-HA	2.38	2.90	2.99	0.09
Leu3HA-HB1	1.65	2.01	2.96	0.95
Leu3HN-HA	1.98	2.42	2.96	0.54
Leu3HB1-HD1	1.75	2.13	2.92	0.78
Leu3HG-HA	1.90	2.32	3.12	0.80
Leu3HN-HG1	2.11	2.57	2.96	0.38
Pro4HA-HD1	2.55	3.11	3.59	0.48
Pro4HA-Leu6HN	2.72	3.32	3.63	0.30
Pro4HD2-Leu3HA	1.59	1.95	2.35	0.40
Pro4HD2-Leu3HN	2.90	3.54	4.66	1.12
Pro4HD1-HG1	1.86	2.28	2.35	0.07
Tyr5HA-HD1	3.50	4.28	3.77	0.00
Tyr5HA-Leu6HN	2.86	3.50	2.87	0.00
Tyr5HA-HB1	2.22	2.72	3.03	0.32
Tyr5HB2-HB1	1.67	2.04	1.74	0.00
Tyr5HN-HB1	2.35	2.87	2.78	0.00
Tyr5HA-HB2	2.24	2.74	2.54	0.00
Tyr5HD2-HB1	2.21	2.71	3.28	0.58
Tyr5HD1-HB2	2.16	2.64	3.25	0.61
Tyr5HB1-HB2	1.85	2.26	1.74	-0.11
Tyr5HN-Pro4HA	1.84	2.24	2.12	0.00
Leu6HA-HN	2.04	2.50	2.94	0.44
Leu6HB1-HD1	2.09	2.55	2.95	0.40
Leu6HN-HB1	2.32	2.84	2.91	0.07

Supplementary Table 17. NOEs considered for the structure calculation of **10a** in $CDCl_3$ and their violations.

10a

Interactions	Lower limit	Upper limit	NOE distance	Violations
Leu1HB1-HA	2.27	2.77	2.60	0.00
Leu1HN-HB1	2.04	2.50	3.31	0.81
Leu1HA-HD1	2.25	2.75	3.00	0.25
Leu1HB1-HD1	1.42	1.74	2.73	0.99
Leu1HA-Leu2HN	1.85	2.26	2.18	0.00
Leu2HB1-HA	1.65	2.01	2.46	0.45
Leu2HN-HA	2.38	2.90	2.93	0.02
Leu2HB1-HN	2.01	2.45	2.73	0.27
Leu2HA-HD1	1.94	2.38	2.78	0.41
Leu2HB1-HD1	1.94	2.38	2.88	0.51
Leu3HN-Leu2HA	2.31	2.83	3.25	0.43
Leu3HA-HB1	1.65	2.01	2.38	0.37
Leu3HN-HA	1.98	2.42	2.90	0.48
Leu3HB2-HD1	2.26	2.76	3.78	1.02
Leu3HA-HD1	2.16	2.64	2.91	0.27
Leu3HG-HA	1.90	2.32	2.79	0.47
Leu3HN-HG	3.82	4.66	4.10	0.00
Pro4HA-HD2	2.55	3.11	4.09	0.98
Pro4HD1-HB1	1.95	2.39	2.76	0.38
Pro4HA-Leu6HN	2.72	3.32	3.49	0.17
Pro4HD1-Leu3HA	1.59	1.95	2.62	0.67
Pro4HD1-Leu3HN	2.90	3.54	4.61	1.06
Pro4HD1-HG1	1.86	2.28	2.37	0.09
Tyr5HA-HD1	2.16	2.64	2.51	0.00
Tyr5HA-Leu6HN	2.86	3.50	3.45	0.00
Tyr5HA-HB1	2.22	2.72	3.05	0.33
Tyr5HB2-HB1	1.67	2.04	1.73	0.00
Tyr5HN-HB1	2.35	2.87	2.61	0.00
Tyr5HA-HB2	2.24	2.74	2.51	0.00
Tyr5HD1-HB2	2.16	2.64	2.44	0.00
Tyr5HN-Pro4HA	1.84	2.24	2.13	0.00
Leu6HA-HN	2.04	2.50	2.90	0.41
Leu6HB1-HD1	2.09	2.55	2.82	0.27
Leu6HN-HB1	2.32	2.84	2.51	0.00

Supplementary Table 18. NOEs considered for the structure calculation of **10e** in $CDCl_3$ and their violations.

10e

Interactions	Lower limit	Upper limit	NOE distance	Violations
Leu1HB1-HA	2.27	2.77	2.59	0.00
Leu1HN-HB1	2.49	3.05	3.35	0.30
Leu1HA-HD1	2.45	2.99	3.00	0.01
Leu1HB1-HD1	1.98	2.42	2.74	0.32
Leu1HA-Leu2HN	1.85	2.26	2.29	0.04
Leu2HB2-HA	1.65	2.01	3.05	1.03
Leu2HN-HA	2.38	2.90	2.85	0.00
Leu2HB2-HN	2.41	2.95	2.56	0.00
Leu2HB1-HD1	1.94	2.38	2.88	0.50
Leu3HN-Leu2HA	2.31	2.83	3.46	0.63
Leu3HA-HB2	1.65	2.01	3.01	0.99
Leu3HN-HA	1.98	2.42	2.88	0.46
Leu3HB1-HD1	1.75	2.13	2.77	0.63
Leu3HG-HA	1.90	2.32	2.77	0.45
Pro4HA-HD1	2.55	3.11	3.37	0.26
Pro4HD1-HB1	2.71	3.31	2.77	0.00
Pro4HA-Leu6HN	2.72	3.32	3.44	0.12
Pro4HD2-Leu3HA	1.98	2.42	2.28	0.00
Pro4HD2-Leu3HN	2.90	3.54	4.06	0.52
Pro4HD1-HG1	1.86	2.28	2.37	0.10
Tyr5HA-HD1	2.16	2.64	2.64	0.00
Tyr5HA-Leu6HN	2.86	3.50	3.28	0.00
Tyr5HA-HB1	2.22	2.72	3.04	0.33
Tyr5HB2-HB1	1.67	2.04	1.74	0.00
Tyr5HN-HB1	2.35	2.87	2.64	0.00
Tyr5HA-HB2	2.24	2.74	2.49	0.00
Tyr5HD2-HB1	2.21	2.71	2.39	0.00
Tyr5HD1-HB2	2.16	2.64	2.40	0.00
Tyr5HN-Pro4HA	1.84	2.24	2.07	0.00
Leu6HA-HN	2.04	2.50	2.90	0.41
Leu6HB1-HD1	2.09	2.55	2.81	0.25
Leu6HN-HB1	2.32	2.84	2.53	0.00

Supplementary Table 19. NOEs considered for the structure calculation of **10f** in $CDCl_3$ and their violations.

10f

Interactions	Lower limit	Upper limit	NOE distance	Violations
Leu1HB1-HA	2.27	2.77	2.64	0.00
Leu1HN-HB1	2.49	3.05	3.32	0.27
Leu1HA-HD1	2.45	2.99	3.01	0.02
Leu1HB1-HD1	1.98	2.42	2.73	0.31
Leu1HA-Leu2HN	1.85	2.26	2.21	0.00
Leu2HB2-HA	2.68	3.28	3.04	0.00
Leu2HN-HA	2.38	2.90	2.88	0.00
Leu2HB2-HN	2.41	2.95	2.53	0.00
Leu2HB1-HD1	1.94	2.38	2.86	0.48
Leu3HN-Leu2HA	2.31	2.83	3.44	0.62
Leu3HA-HB2	1.65	2.01	2.82	0.81
Leu3HN-HA	1.98	2.42	2.76	0.34
Leu3HB1-HD1	1.75	2.13	2.78	0.65
Leu3HG-HA	1.90	2.32	2.45	0.13
Pro4HA-HD1	2.55	3.11	3.33	0.22
Pro4HD1-HB1	2.71	3.31	2.80	0.00
Pro4HA-Leu6HN	2.72	3.32	3.49	0.17
Pro4HD2-Leu3HA	1.98	2.42	2.18	0.00
Pro4HD2-Leu3HN	2.90	3.54	4.40	0.86
Pro4HD1-HG1	1.86	2.28	2.38	0.10
Tyr5HA-HD1	2.16	2.64	2.63	0.00
Tyr5HA-Leu6HN	2.86	3.50	3.03	0.00
Tyr5HA-HB1	2.22	2.72	3.05	0.33
Tyr5HB2-HB1	1.67	2.04	1.73	0.00
Tyr5HN-HB1	2.35	2.87	2.70	0.00
Tyr5HA-HB2	2.24	2.74	2.49	0.00
Tyr5HD2-HB1	2.21	2.71	2.38	0.00
Tyr5HD1-HB2	2.16	2.64	2.42	0.00
Tyr5HN-Pro4HA	1.84	2.24	2.09	0.00
Leu6HA-HN	2.04	2.50	2.94	0.44
Leu6HB1-HD1	2.09	2.55	2.80	0.25
Leu6HN-HB1	2.32	2.84	2.74	0.00

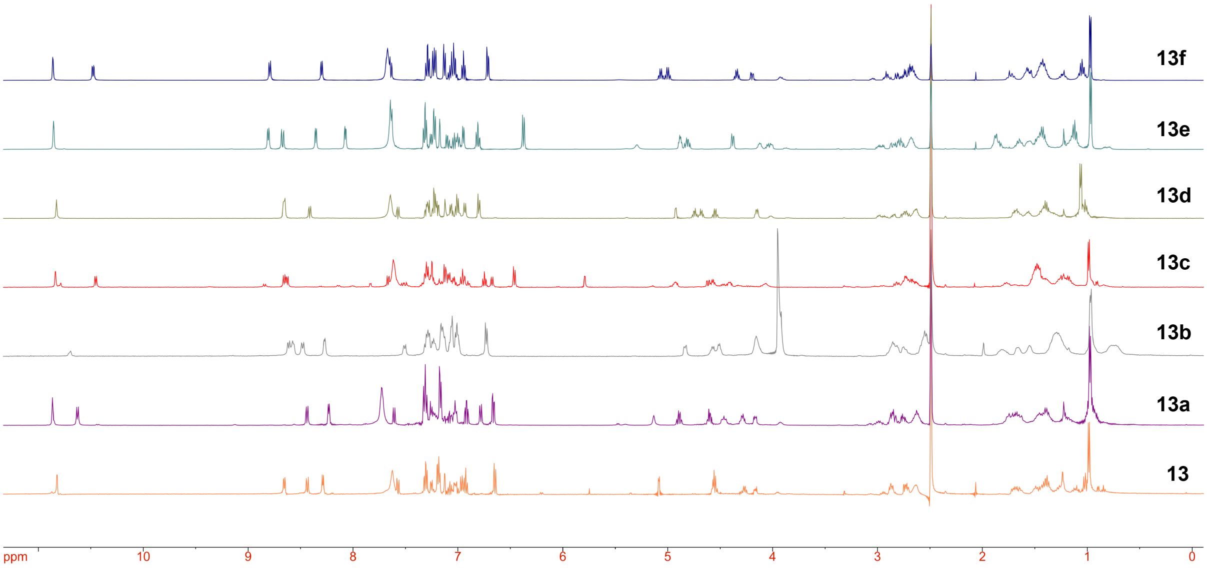

Supplementary Figure 75. Proton (^{1}H) NMR spectra in DMSO-d_6. ^{1}H NMR overlay of **13**, **13a-f** in DMSO-d_6.

a

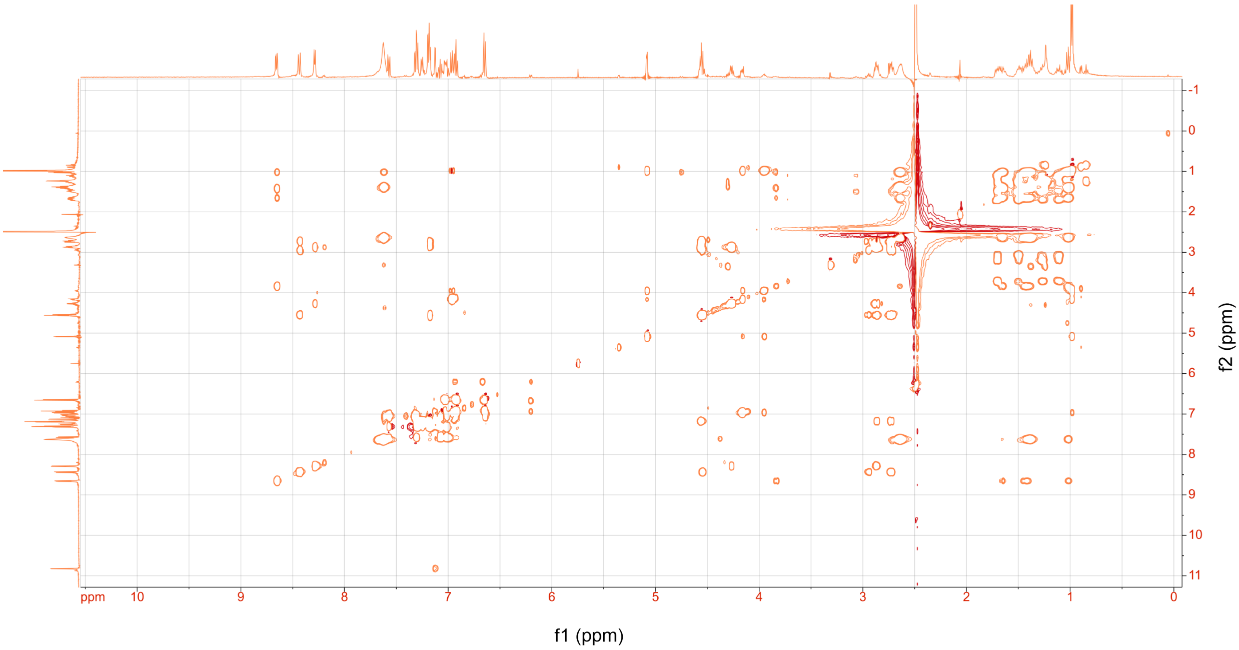

b

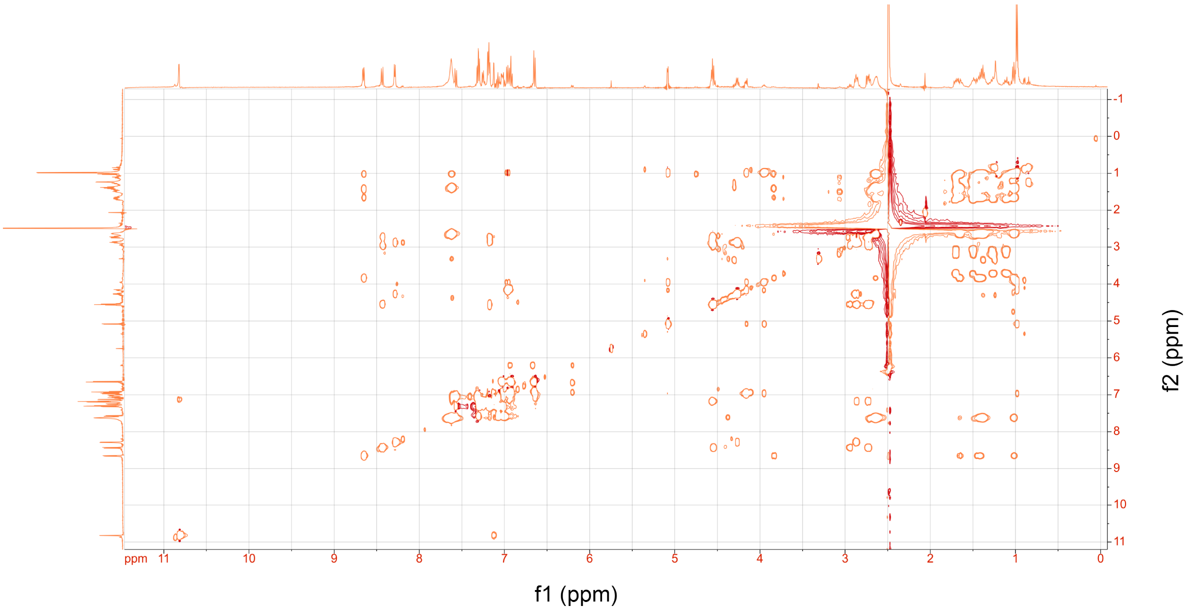

Supplementary Figure 76. Two-dimensional ^{1}H NMR spectra in DMSO-d_6. TOCSY (**a**) and ROESY (**b**) spectra of **13**.

a

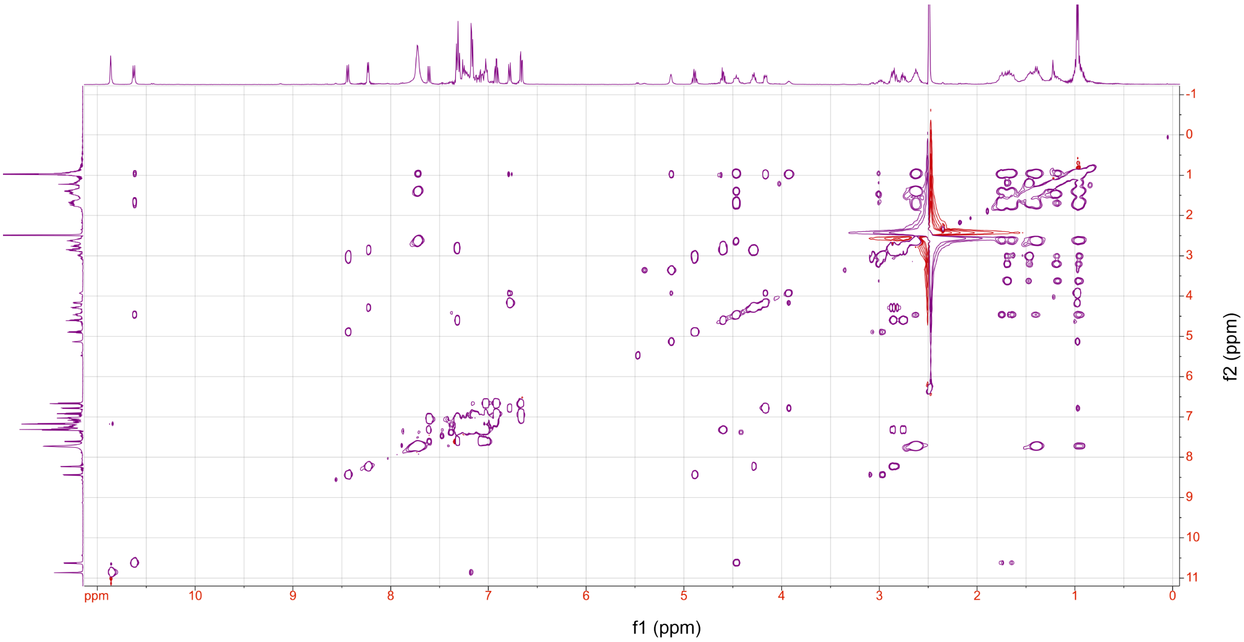

b

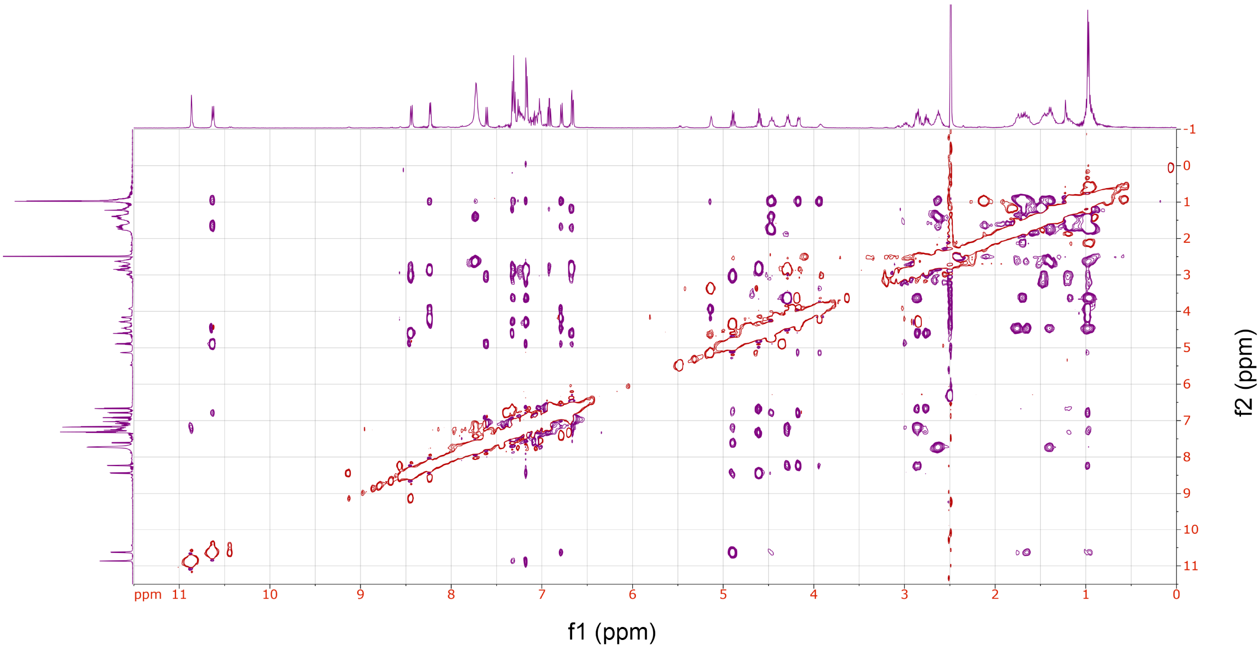

Supplementary Figure 77. Two-dimensional ^{1}H NMR spectra in DMSO-*d*$_6$. TOCSY (**a**) and ROESY (**b**) spectra of **13a.**

a

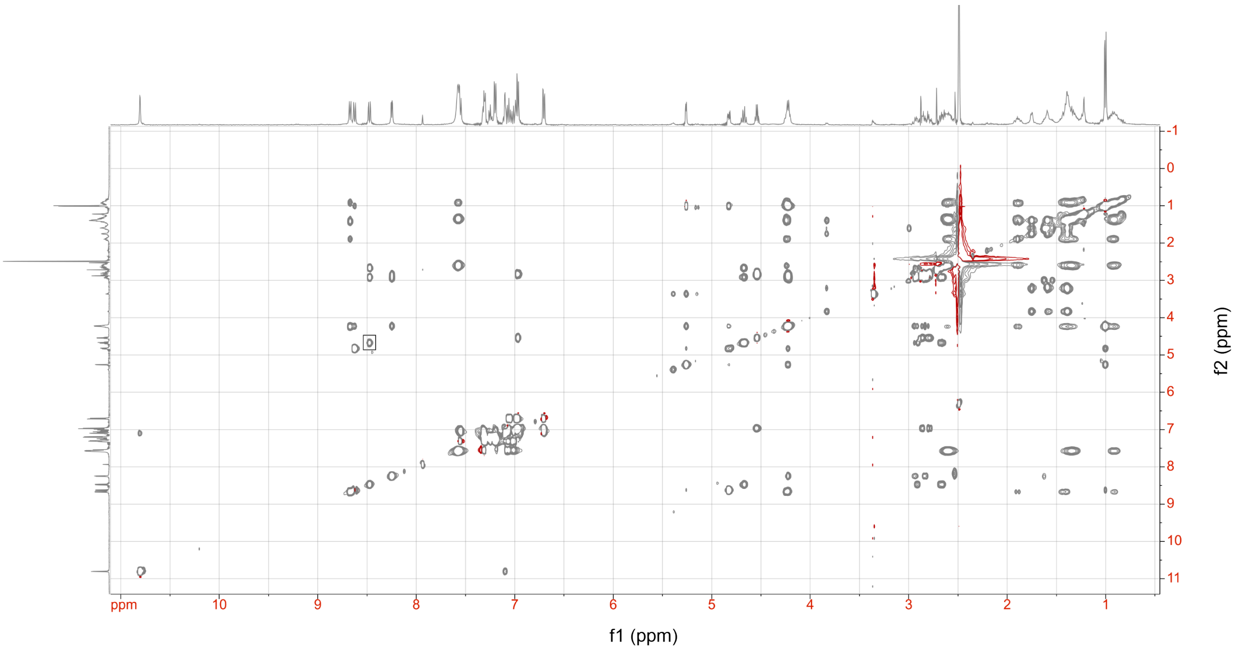

b

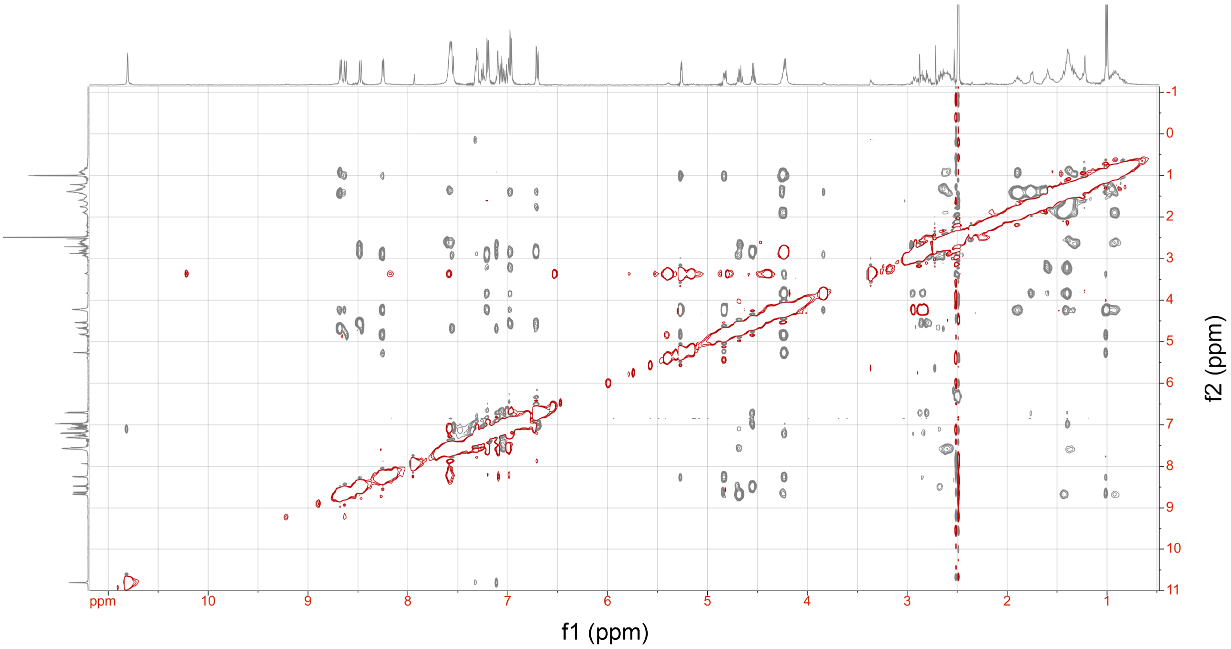

Supplementary Figure 78. Two-dimensional ^{1}H NMR spectra in DMSO-*d*$_6$. TOCSY (**a**) and ROESY (**b**) spectra of **13b.**

a

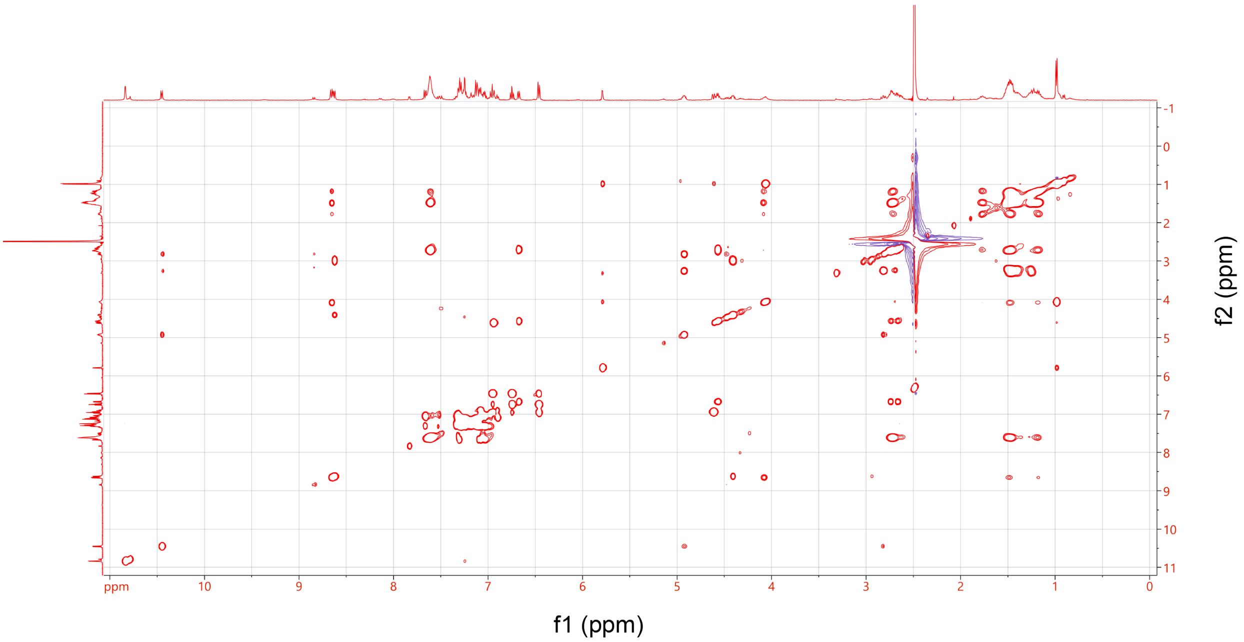

b

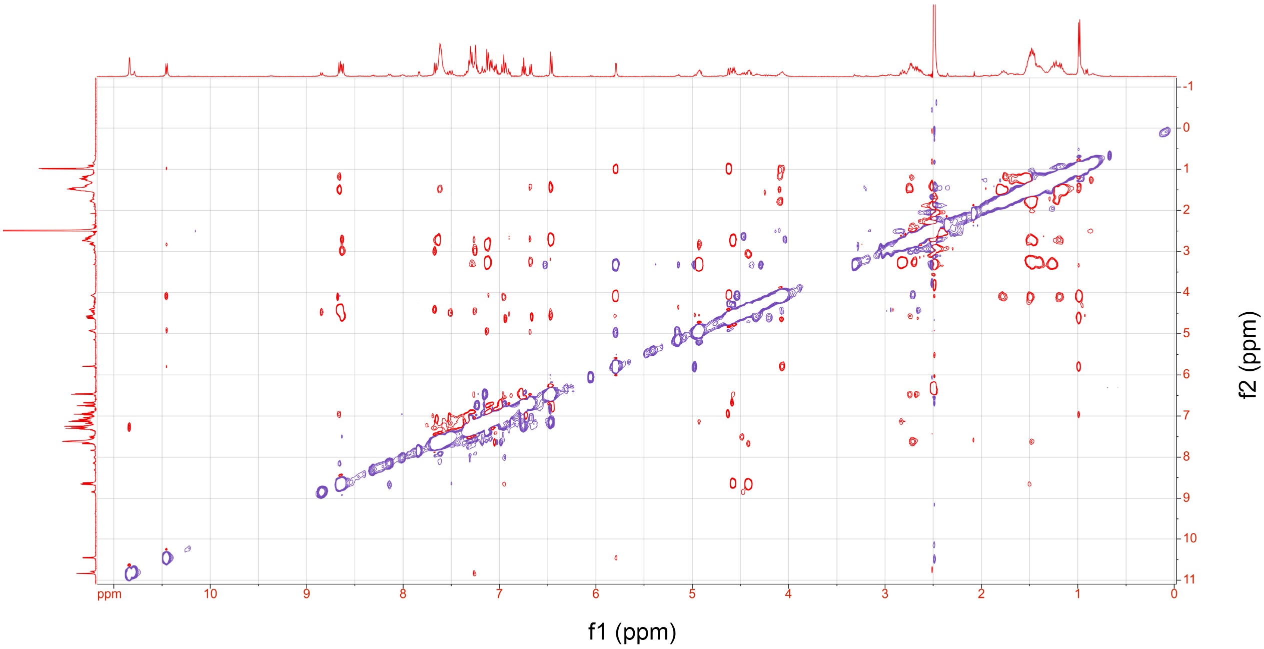

Supplementary Figure 79. Two-dimensional ^{1}H NMR spectra in DMSO-d_6. TOCSY (**a**) and ROESY (**b**) spectra of **13c.**

a

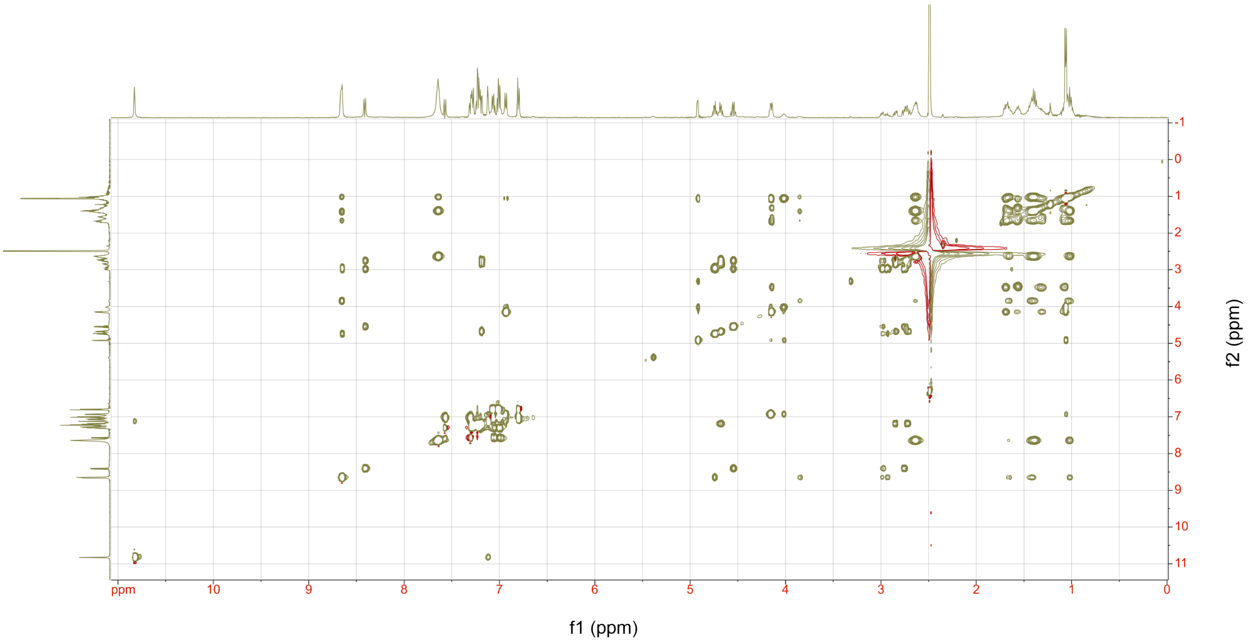

b

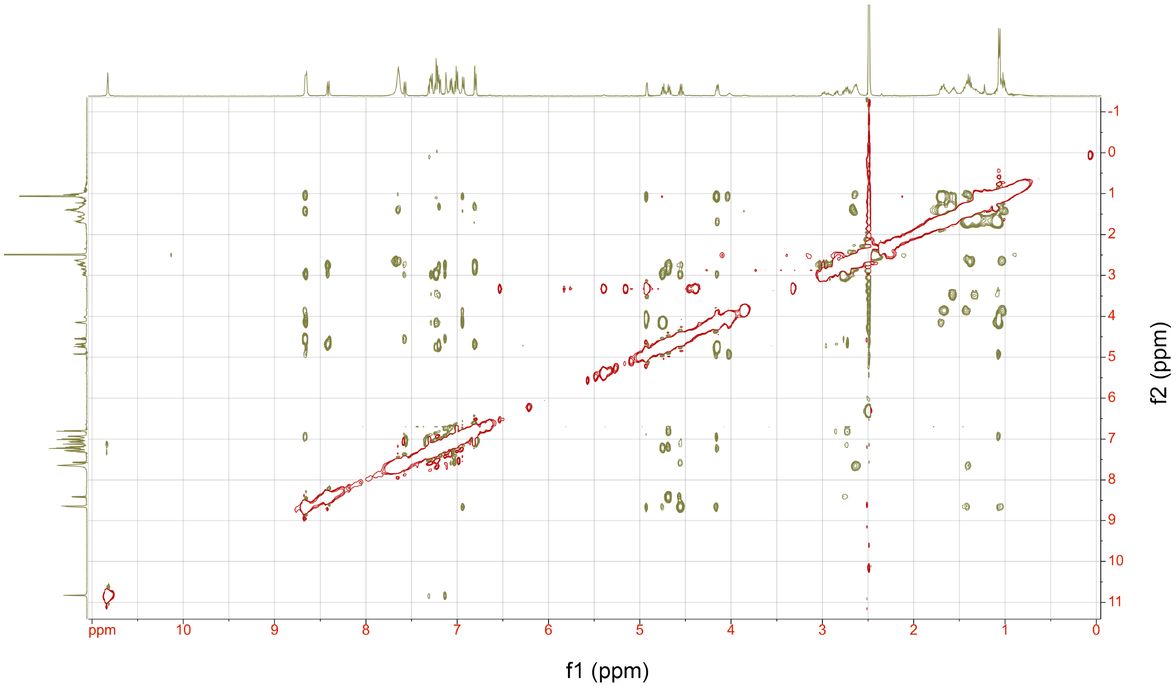

Supplementary Figure 80. Two-dimensional ^{1}H NMR spectra in DMSO-d_6. TOCSY (**a**) and ROESY (**b**) spectra of **13d.**

a

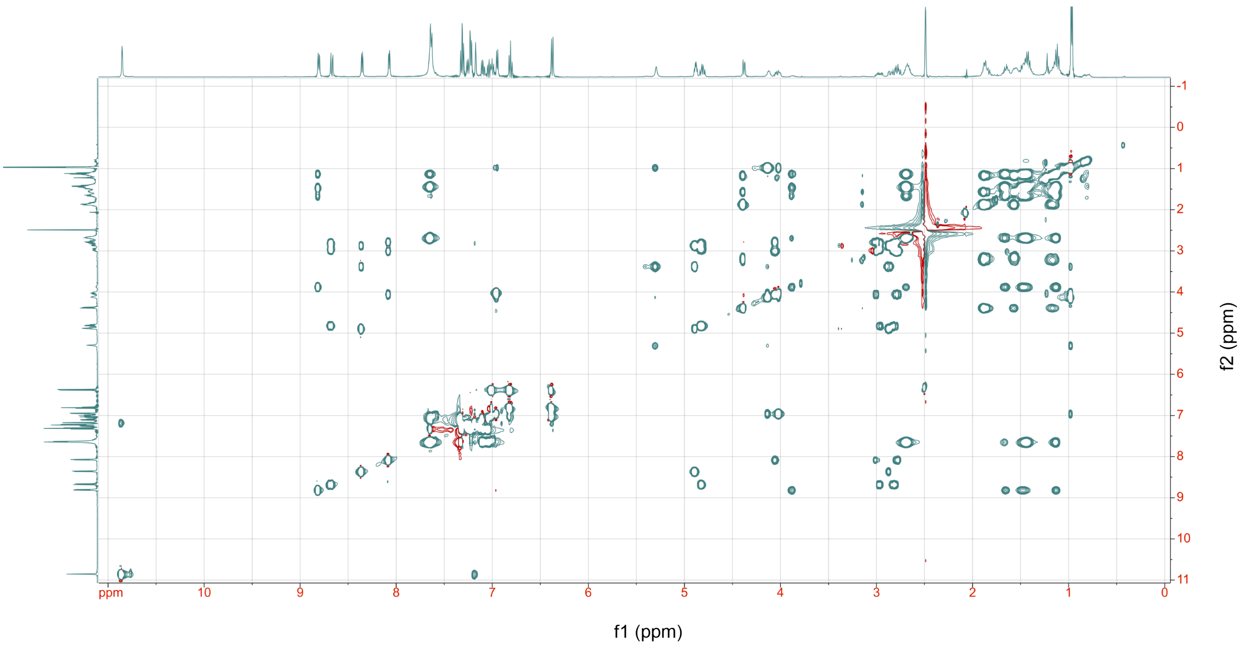

b

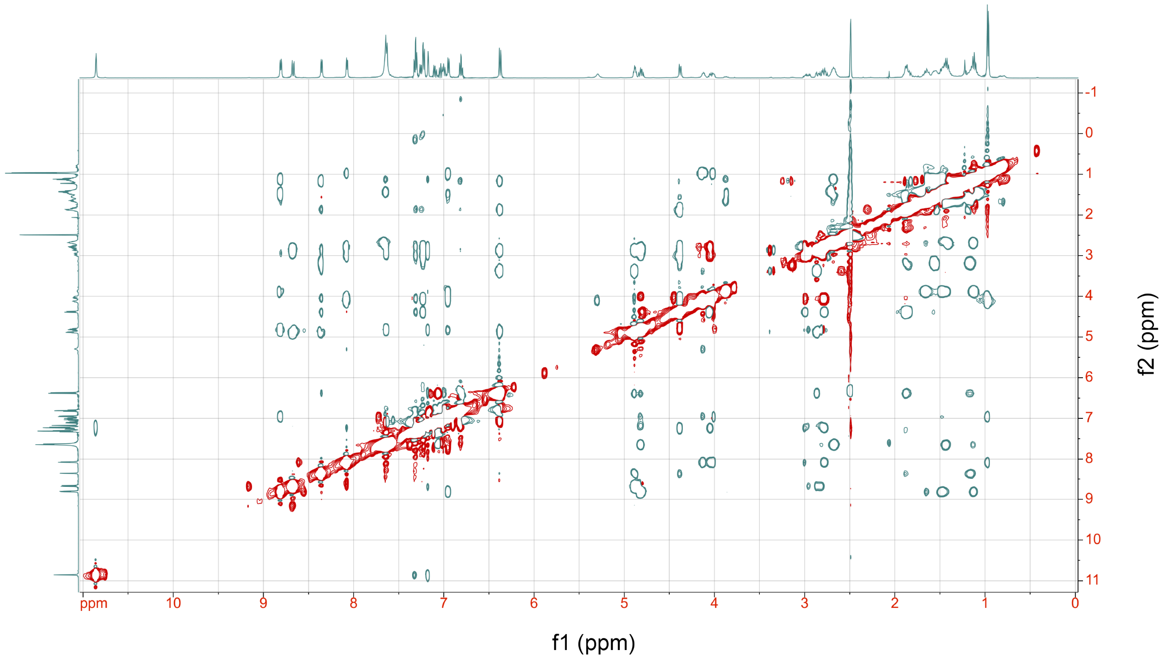

Supplementary Figure 81. Two-dimensional ^{1}H NMR spectra in DMSO-d_6. TOCSY (**a**) and ROESY (**b**) spectra of **13e.**

a

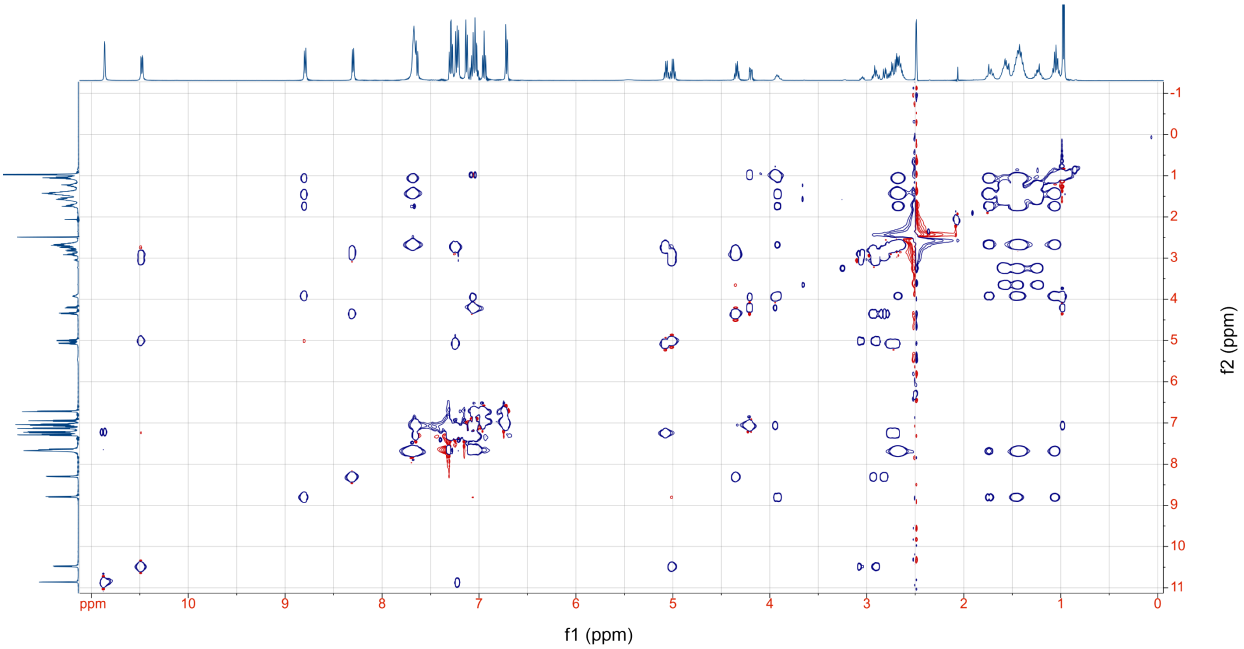

b

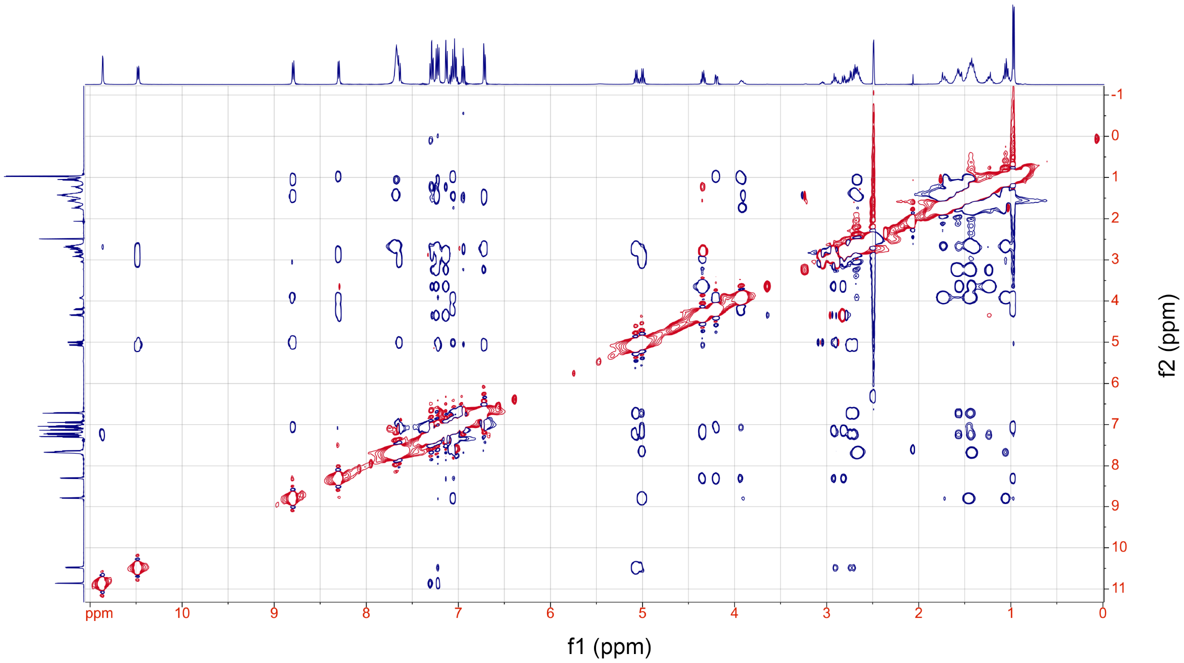

Supplementary Figure 82. Two-dimensional ^{1}H NMR spectra in DMSO-d_6. TOCSY (**a**) and ROESY (**b**) spectra of **13f.**

Supplementary Table 20. Absolute proton chemical shifts of **13** and thio analogs in DMSO-d_6.

		13	13a	13b	13c	13d	13e	13f
D-Trp1	HN	8.45	8.43	8.47	8.60	8.40	8.68	10.48
	HA	4.57	4.90	4.68	4.42	4.53	4.85	4.99
	HB	2.72/2.95	3.03	2.66/2.92	2.93/3.08	2.76/2.97	2.82/2.98	2.91/3.09
Lys2	HN	8.67	10.61	8.67	8.64	8.65	8.81	8.80
	HA	3.84	4.47	4.24	4.09	3.84	3.89	3.93
	HB	1.65	1.67	1.88	1.78	1.68	1.68	1.73
	HG	1.01	0.95	0.92	1.16	1.03	1.12	1.07
	HD	1.42	1.39	1.41	1.50	1.42	1.46	1.46
	HE	2.98	2.99	3.00	2.99	2.99	2.99	2.98
Thr3	HN	6.98	6.76	8.62	6.95	6.93	6.96	7.05
	HA	4.12	4.16	4.83	4.59	4.18	4.18	4.22
	HB	3.93	3.93	4.22	4.09	4.03	4.04	3.98
	HG1	0.88	0.98	1.00	1.00	1.05	0.96	0.98
	HG2	5.38	5.11	5.27	5.77	4.93	5.29	5.13
Phe4	HN	8.30	8.23	8.24	10.44	8.64	8.36	8.31
	HA	4.28	4.31	4.23	4.95	4.75	4.91	4.37
	HB	2.85	2.80/2.89	2.89	2.82/3.36	2.95	2.87/3.38	2.80/2.97
Pro5	HA	3.71	3.66	3.83	4.03	4.18	4.38	3.65
	HB	1.69	1.67	1.70	1.67	1.64	1.55/1.87	1.43/1.58
	HG	1.09	1.42	1.40	1.41	1.42	1.15	1.23
	HD	3.06	3.00	3.22	3.25	3.48	3.22	3.26
Phe6	HN	7.19	7.32	6.96	6.65	7.18	8.07	7.24
	HA	4.58	4.60	4.55	4.56	4.68	4.08	5.13
	HB	2.70/2.90	2.80	2.84	2.64/2.72	2.70/2.84	2.79/3.03	2.76

Supplementary Table 21. Coupling constant and dihedral angle[a] determined from ^{1}H NMR in DMSO-d_6.

Residues	13		13a		13b		13c		13d		13e		13f	
	$^3J_{NH}$	θ	$^3J_{NH}$	θ	$^3J_{NH}$	θ	$^3J_{NH}$	θ	$^3J_{NH}$	θ	$^3J_{NH}$	θ	$^3J_{NH}$	θ
D-Trp1	8	147.4	8	147.4	9.7	159.7	8	147.4	9.2	155.6	9.7	159.7	7.8	146.2
Lys2	6.9	140.8	8	147.4	7	141.4	7.9	146.8	n.d.	n.d.	7.6	144.9	7.8	146.2
Thr3	7.8	146.2	8	147.4	7.8	146.2	n.d.	n.d.	8.1	148	6.9	140.8	n.d.	n.d.
Phe4	5.8	134.6	5.4	132.3	6	135.7	7.8	146.2	n.d.	n.d.	5.9	135.1	6	135.7
Phe6	7.7	145.6	n.d.	n.d.	7.3	143.2	8.7	n.d.	n.d.	n.d.	5.8	134.6	n.d.	n.d.

n.d.: could not be determined.

[a] θ is the dihedral angle formed between H-N-Cα-Hα.

Supplementary Table 22. NOEs considered for the structure calculation of **13** in DMSO-d_6 and their violations.

13

Interactions	Lower limit	Upper limit	NOE distance	Violations
Dtr1HA-HN	2.09	2.55	2.91	0.36
Dtr1HA-Lys2HN	2.26	2.76	2.17	-0.09
Dtr1HB2-HA	1.96	2.40	2.52	0.12
Dtr1HB2-HN	2.49	3.04	2.83	0.00
Lys2HA-HB2	2.33	2.85	2.53	0.00
Lys2HG2-HN	2.81	3.43	2.64	-0.17
Lys2HN-HB2	3.60	4.40	3.67	0.00
Lys2HN-Thr3HN	2.99	3.66	2.75	-0.24
Thr3HA-HG1	2.15	2.63	2.51	0.00
Thr3HA-HN	2.58	3.15	2.81	0.00
Thr3HB-HG1	2.26	2.76	2.79	0.03
Thr3HG2-HB	1.97	2.81	2.48	0.00
Thr3HB-HN	2.69	3.29	2.45	-0.25
Thr3HB-Phe4HN	3.08	3.76	3.77	0.01
Thr3HG1-HG2	2.55	3.12	2.93	0.00
Thr3HG2-HA	2.62	3.61	3.10	0.00
Thr3HG2-HG1	2.50	3.46	2.93	0.00
Thr3HG2-HN	2.92	3.97	4.30	0.33
Thr3HG2-Phe4HN	3.24	4.35	3.09	-0.14
Phe4HA-HN	2.34	2.87	2.87	0.00
Phe4HD1-HB2	2.59	3.17	2.70	0.00
Phe4HN-Thr3HA	1.81	2.22	2.12	0.00
Phe4HN-Thr3HG1	2.85	3.48	4.18	0.70
Phe4HN-Thr3HG2	3.26	3.98	3.09	-0.16
Pro5HA-Phe4HA	1.97	2.40	2.08	0.00
Pro5HA-Phe4HB2	2.57	3.14	3.83	0.68
Pro5HA-Phe4HD1	2.49	3.04	2.67	0.00
Pro5HA-HG1	2.25	2.75	3.68	0.94
Pro5HD2-HB2	2.32	2.83	3.03	0.20
Phe6HA-HN	2.68	3.27	2.93	0.00
Phe6HB2-HN	2.08	2.54	2.64	0.10
Pro5HA-Phe4HD1	2.49	3.05	2.67	0.00
Phe4HB1-Phe4HD1	3.32	4.06	3.80	0.00
Pro5HG1-Phe4HD1	4.80	5.86	4.27	-0.53
Dtr1HZ2-Lys2HA	2.75	3.36	4.27	0.91
Dtr1HZ2-Lys2HD2	2.93	3.58	4.27	0.69
Dtr1HZ2-Lys2HG2	3.53	4.32	4.27	0.00

Supplementary Table 23. NOEs considered for the structure calculation of **13a** in DMSO-d_6 and their violations.

13a

Interactions	Lower limit	Upper limit	NOE distance	Violations
Dtr1HA-Lys2HN	2.36	2.89	2.10	-0.27
Dtr1HB1-HA	2.25	2.75	3.05	0.30
Dtr1HB1-HB2	2.75	3.36	1.75	-1.00
Dtr1HB1-HN	2.67	3.27	2.68	0.00
Dtr1HB2-HA	2.27	2.78	2.52	0.00
Dtr1HB2-HN	2.14	2.61	2.63	0.02
Dtr1HN-Lys2HN	3.48	4.25	4.36	0.11
Lys2HD2-Dtr1HZ2	3.17	3.87	3.69	0.00
Lys2HG2-Dtr1HZ2	3.53	4.31	3.85	0.00
Lys2HD2-HB2	2.70	3.30	3.53	0.23
Thr3HA-HN	2.79	3.41	2.93	0.00
Thr3HA-Phe4HN	2.43	2.97	2.33	-0.10
Thr3HB-HG1	2.10	2.56	2.43	0.00
Thr3HB-HN	2.20	2.68	2.54	0.00
Thr3HG2-HA	2.47	3.42	3.00	0.00
Thr3HG2-HB	2.72	3.73	2.49	-0.23
Thr3HG2-HG1	2.93	3.98	3.45	0.00
Thr3HG2-HN	3.25	4.37	4.45	0.08
Phe4HB1-HA	2.46	3.00	2.53	0.00
Phe4HB1-HD1	3.23	3.95	3.67	0.00
Phe4HB1-HN	2.99	3.66	3.04	0.00
Phe4HB1-Pro5HA	2.94	3.60	3.28	0.00
Pro5HA-Phe4HA	1.97	2.40	2.61	0.21
Pro5HB2-HA	2.84	3.48	2.28	-0.56
Pro5HG2-HB2	2.08	2.55	2.39	0.00
Phe6HA-Dtr1HN	2.29	2.80	2.33	0.00
Phe6HA-HD1	2.77	3.39	2.87	0.00
Phe6HA-HN	2.43	2.97	2.90	0.00
Phe6HB2-HA	2.08	2.54	2.54	0.00

Supplementary Table 24. NOEs considered for the structure calculation of **13b** in DMSO-d_6 and their violations.

13b

Interactions	Lower limit	Upper limit	NOE distance	Violations
Thr3HA-Phe4HN	2.24	2.74	2.26	0.00
Dtr1HA-Lys2HN	2.04	2.50	2.17	0.00
Dtr1HZ2-Lys2HA	2.75	3.36	4.19	0.83
Dtr1HZ2-Lys2HD2	2.93	3.58	4.23	0.65
Dtr1HZ2-Lys2HG2	3.53	4.32	4.24	0.00
Dtr1HB1-HN	2.57	3.14	2.99	0.00
Phe4HA-HN	2.26	2.76	2.71	0.00
Phe4HB1-HN	2.20	2.69	2.54	0.00
Phe6HA-Dtr1HN	1.99	2.43	2.30	0.00
Dtr1HB2-HN	2.16	2.64	2.52	0.00
Thr3HB-HN	2.88	3.52	3.17	0.00
Lys2HA-HN	2.60	3.18	2.89	0.00
Phe6HA-HN	2.26	2.77	2.91	0.15
Phe4HA-Phe6HN	2.85	3.48	3.04	0.00
Phe6HB1-Phe6HN	2.35	2.87	2.58	0.00
Thr3HG2-HA	2.36	3.28	2.91	0.00
Thr3HG2-HB	1.97	2.81	2.56	0.00
Thr3HA-HG1	2.67	3.27	3.27	0.00
Thr3HB-HA	1.89	2.31	2.77	0.47
Pro5HA-Phe4HA	1.89	2.31	2.27	0.00
Dtr1HB2-HA	2.40	2.93	3.05	0.12
Lys2HB1-HA	2.05	2.50	2.46	0.00
Phe6HB1-HA	1.99	2.43	2.43	0.00
Pro5HA-HB1	2.11	2.58	2.73	0.16
Phe4HA-HD2	2.37	2.89	2.64	0.00
Pro5HA-Phe4HD2	2.36	2.88	2.78	0.00

Supplementary Table 25. NOEs considered for the structure calculation of **13c** in DMSO-d_6 and their violations.

13c

Interactions	Lower limit	Upper limit	NOE distance	Violations
Dtr1HA-HN	2.09	2.55	2.92	0.37
Dtr1HA-Lys2HN	2.26	2.76	2.64	0.00
Dtr1HB2-HA	1.96	2.40	2.51	0.11
Dtr1HB2-HN	2.49	3.04	2.71	0.00
Lys2HA-HB2	2.33	2.85	2.59	0.00
Lys2HN-HA	1.94	2.37	2.84	0.47
Lys2HG2-HN	2.81	3.43	4.33	-0.90
Lys2HN-HB2	3.60	4.40	3.91	0.00
Lys2HN-Thr3HN	2.99	3.66	2.00	-1.00
Thr3HA-HG1	2.15	2.63	3.53	0.90
Thr3HA-HN	2.58	3.15	2.87	0.00
Thr3HB-HG1	2.26	2.76	2.42	0.00
Thr3HG2-HB	1.97	2.81	2.49	0.00
Thr3HB-HN	2.69	3.29	3.33	0.04
Thr3HB-Phe4HN	3.08	3.76	3.26	0.00
Thr3HG1-HG2	2.55	3.12	3.51	0.39
Thr3HG2-HA	2.62	3.61	2.95	0.00
Thr3HG2-HG1	2.50	3.46	3.51	0.06
Thr3HG2-HN	2.92	3.97	3.47	0.00
Thr3HG2-Phe4HN	3.24	4.35	4.51	0.16
Phe4HA-HN	2.34	2.87	2.83	0.00
Phe4HD1-HB2	2.59	3.17	3.08	0.00
Phe4HN-Thr3HA	1.81	2.22	2.30	0.09
Phe4HN-Thr3HG1	2.85	3.48	4.56	1.08
Phe4HN-Thr3HG2	3.26	3.98	4.51	0.53
Pro5HA-Phe4HA	1.97	2.40	2.19	0.00
Pro5HA-Phe4HB2	2.57	3.14	3.10	0.00
Pro5HA-Phe4HD1	2.49	3.04	4.02	0.89
Pro5HA-HG1	2.25	2.75	2.94	0.23
Pro5HD2-HB2	2.32	2.83	3.14	0.31
Phe6HA-HN	2.68	3.27	2.84	0.00
Phe6HB2-HN	2.08	2.54	2.59	0.05

Supplementary Table 26. NOEs considered for the structure calculation of **13d** in DMSO-d_6 and their violations.

13d

Interactions	Lower limit	Upper limit	NOE distance	Violations
Dtr1HA-HN	2.09	2.55	2.90	0.36
Dtr1HA-Lys2HN	2.26	2.76	2.19	-0.07
Dtr1HB2-HA	1.96	2.40	2.65	0.25
Dtr1HB2-HN	2.49	3.04	2.80	0.00
Lys2HA-HB2	2.33	2.85	2.54	0.00
Lys2HG2-HN	2.81	3.43	2.63	-0.17
Lys2HN-HB2	3.60	4.40	3.66	0.00
Lys2HN-Thr3HN	2.99	3.66	2.74	-0.25
Thr3HA-HG1	2.15	2.63	2.72	0.10
Thr3HA-HN	2.58	3.15	2.84	0.00
Thr3HB-HG1	2.26	2.76	2.75	0.00
Thr3HG2-HB	1.97	2.81	2.47	0.00
Thr3HB-HN	2.69	3.29	2.59	-0.10
Thr3HB-Phe4HN	3.08	3.76	3.85	0.09
Thr3HG1-HG2	2.55	3.12	3.02	0.00
Thr3HG2-HA	2.62	3.61	2.97	0.00
Thr3HG2-HG1	2.50	3.46	3.02	0.00
Thr3HG2-HN	2.92	3.97	4.29	0.32
Thr3HG2-Phe4HN	3.24	4.35	3.50	0.00
Phe4HA-HN	2.34	2.87	2.84	0.00
Phe4HD1-HB2	2.59	3.17	2.67	0.00
Phe4HN-Thr3HA	1.81	2.22	2.22	0.00
Phe4HN-Thr3HG2	3.26	3.98	3.50	0.00
Pro5HA-Phe4HA	1.97	2.40	2.09	0.00
Pro5HA-Phe4HB2	2.57	3.14	3.83	0.68
Pro5HA-Phe4HD1	2.49	3.04	2.73	0.00
Pro5HA-HG1	2.25	2.75	3.89	1.15
Pro5HD2-HB2	2.32	2.83	3.07	0.24
Phe6HA-HN	2.68	3.27	2.93	0.00
Phe6HB2-HN	2.08	2.54	2.61	0.07
Pro5HA-Phe4HD1	2.49	3.05	2.73	0.00
Phe4HB1-Phe4HD1	3.32	4.06	3.77	0.00
Pro5HG1-Phe4HD1	4.80	5.86	4.48	-0.31
Dtr1HZ2-Lys2HD2	4.82	5.90	8.00	2.10
Dtr1HZ2-Lys2HG2	4.48	5.48	5.60	0.12

Supplementary Table 27. NOEs considered for the structure calculation of **13e** in DMSO-d_6 and their violations.

13e

Interactions	Lower limit	Upper limit	NOE distance	Violations
Dtr1HA-HN	2.09	2.55	2.90	0.35
Dtr1HA-Lys2HN	2.26	2.76	2.22	-0.04
Dtr1HB2-HA	1.96	2.40	2.53	0.14
Dtr1HB2-HN	2.49	3.04	2.95	0.00
Lys2HA-HB2	2.33	2.85	2.54	0.00
Lys2HG2-HN	2.81	3.43	2.68	-0.13
Lys2HN-HB2	3.60	4.40	3.67	0.00
Lys2HN-Thr3HN	2.99	3.66	2.67	-0.32
Thr3HA-HG1	2.15	2.63	2.73	0.10
Thr3HA-HN	2.58	3.15	2.83	0.00
Thr3HB-HG1	2.26	2.76	2.76	0.00
Thr3HG2-HB	1.97	2.81	2.48	0.00
Thr3HB-HN	2.69	3.29	2.59	-0.10
Thr3HB-Phe4HN	3.08	3.76	3.67	0.00
Thr3HG1-HG2	2.55	3.12	2.99	0.00
Thr3HG2-HA	2.62	3.61	2.97	0.00
Thr3HG2-HG1	2.50	3.46	2.99	0.00
Thr3HG2-HN	2.92	3.97	4.26	0.29
Thr3HG2-Phe4HN	3.24	4.35	3.21	-0.02
Phe4HA-HN	2.34	2.87	2.83	0.00
Phe4HD1-HB2	2.59	3.17	2.64	0.00
Phe4HN-Thr3HA	1.81	2.22	2.24	0.03
Phe4HN-Thr3HG2	3.26	3.98	3.21	-0.04
Pro5HA-Phe4HA	1.97	2.40	2.14	0.00
Pro5HA-Phe4HB2	2.57	3.14	3.76	0.62
Pro5HA-Phe4HD1	2.49	3.04	2.76	0.00
Pro5HA-HG1	2.25	2.75	3.89	1.14
Pro5HD2-HB2	2.32	2.83	3.07	0.24
Phe6HA-HN	2.68	3.27	2.91	0.00
Phe6HB2-HN	2.08	2.54	2.72	0.18
Pro5HA-Phe4HD1	2.49	3.05	2.76	0.00
Phe4HB1-Phe4HD1	3.32	4.06	3.77	0.00
Pro5HG1-Phe4HD1	4.80	5.86	4.52	-0.28
Dtr1HZ2-Lys2HD2	4.82	5.90	6.50	0.60
Dtr1HZ2-Lys2HG2	4.48	5.48	5.20	-0.28

Supplementary Table 28. NOEs considered for the structure calculation of **13f** in DMSO-d_6 and their violations.

13f

Interactions	Lower limit	Upper limit	NOE distance	Violations
Dtr1HA-HN	2.09	2.55	2.84	0.29
Dtr1HA-Lys2HN	2.26	2.76	2.30	0.00
Dtr1HB2-HA	1.96	2.40	2.56	0.16
Dtr1HB2-HN	2.49	3.05	3.21	0.16
Lys2HA-HB2	2.33	2.85	2.57	0.00
Lys2HG2-HN	2.81	3.43	2.72	-0.09
Lys2HN-HB2	3.60	4.40	3.67	0.00
Lys2HN-Thr3HN	3.00	3.66	2.83	-0.16
Thr3HA-HG1	2.15	2.63	2.74	0.11
Thr3HA-HN	2.57	3.15	2.87	0.00
Thr3HB-HG1	2.26	2.76	2.72	0.00
Thr3HG2-HB	1.97	2.81	2.46	0.00
Thr3HB-HN	2.69	3.29	2.59	-0.10
Thr3HB-Phe4HN	3.08	3.76	3.92	0.16
Thr3HG1-HG2	2.56	3.12	3.10	0.00
Thr3HG2-HA	2.63	3.61	2.97	0.00
Thr3HG2-HG1	2.50	3.46	3.10	0.00
Thr3HG2-HN	2.93	3.98	4.34	0.37
Thr3HG2-Phe4HN	3.23	4.35	3.55	0.00
Phe4HA-HN	2.35	2.87	2.85	0.00
Phe4HD1-HB2	2.59	3.17	2.63	0.00
Phe4HN-Thr3HA	1.82	2.22	2.20	0.00
Phe4HN-Thr3HG2	3.26	3.98	3.55	0.00
Pro5HA-Phe4HA	1.97	2.41	2.14	0.00
Pro5HA-Phe4HB2	2.57	3.15	3.80	0.65
Pro5HA-Phe4HD1	2.49	3.05	2.78	0.00
Pro5HD2-HB2	2.31	2.83	3.10	0.27
Phe6HA-HN	2.68	3.28	2.87	0.00
Phe6HB2-HN	2.08	2.54	2.53	0.00
Pro5HA-Phe4HD1	2.49	3.05	2.78	0.00
Phe4HB1-Phe4HD1	3.32	4.06	3.77	0.00
Pro5HG1-Phe4HD1	4.80	5.86	4.51	-0.29
Dtr1HZ2-Lys2HD2	3.83	4.69	4.46	0.00
Dtr1HZ2-Lys2HG2	4.44	5.42	4.51	0.00

Supplementary References.

1. Chatterjee, J., Laufer, B. & Kessler, H. Synthesis of N-methylated cyclic peptides. *Nat. Protoc.* **7**, 432-444 (2012).

2. Khatri, B., Bhat, P. & Chatterjee, J. Convenient synthesis of thioamidated peptides and proteins. *J. Pep. Sci.* **26**, e3248 (2020).

3. T. D. Goddard, D.G.K. SPARKY 3. (2004).

4. Brooks, B.R. et al. CHARMM: A program for macromolecular energy, minimization, and dynamics calculations. *J. Comput. Chem.* **4**, 187-217 (1983).

5. Madhavi Sastry, G., Adzhigirey, M., Day, T., Annabhimoju, R. & Sherman, W. Protein and ligand preparation: parameters, protocols, and influence on virtual screening enrichments. *J. Comput. Aided Mol. Des.* **27**, 221-234 (2013).

6. Harder, E. et al. OPLS3: A Force Field Providing Broad Coverage of Drug-like Small Molecules and Proteins. *J. Chem. Theory Comput.* **12**, 281-296 (2016).

7. Over, B. et al. Structural and conformational determinants of macrocycle cell permeability. *Nat. Chem. Biol.* **12**, 1065-1074 (2016).

8. Wang, J., Yadav, V., Smart, A.L., Tajiri, S. & Basit, A.W. Toward Oral Delivery of Biopharmaceuticals: An Assessment of the Gastrointestinal Stability of 17 Peptide Drugs. *Mol. Pharm.* **12**, 966-973 (2015).

9. Brian Houston, J. Utility of in vitro drug metabolism data in predicting in vivo metabolic clearance. *Biochem. Pharmacol.* **47**, 1469-1479 (1994).
