# Supplementary material for: An amide to thioamide substitution improves the permeability and bioavailability of macrocyclic peptides
Source: Nat Commun. 2023 Sep 28;14:6050. doi: 10.1038/s41467-023-41748-y (PMC10539501; doi:10.1038/s41467-023-41748-y)
Supplement: Supplementary file 2 — Reporting Summary [file 41467_2023_41748_MOESM2_ESM.pdf]

nature portfolio

Corresponding author(s): Jayanta Chatterjee

Last updated by author(s): Aug 14, 2023

Reporting Summary

Nature Portfolio wishes to improve the reproducibility of the work that we publish. This form provides structure for consistency and transparency in reporting. For further information on Nature Portfolio policies, see our Editorial Policies and the Editorial Policy Checklist.

Statistics

For all statistical analyses, confirm that the following items are present in the figure legend, table legend, main text, or Methods section.

n/a	Confirmed	
☐	☒	The exact sample size (*n*) for each experimental group/condition, given as a discrete number and unit of measurement
☐	☒	A statement on whether measurements were taken from distinct samples or whether the same sample was measured repeatedly
☐	☒	The statistical test(s) used AND whether they are one- or two-sided<br>*Only common tests should be described solely by name; describe more complex techniques in the Methods section.*
☒	☐	A description of all covariates tested
☒	☐	A description of any assumptions or corrections, such as tests of normality and adjustment for multiple comparisons
☐	☒	A full description of the statistical parameters including central tendency (e.g. means) or other basic estimates (e.g. regression coefficient) AND variation (e.g. standard deviation) or associated estimates of uncertainty (e.g. confidence intervals)
☐	☒	For null hypothesis testing, the test statistic (e.g. *F*, *t*, *r*) with confidence intervals, effect sizes, degrees of freedom and *P* value noted<br>*Give P values as exact values whenever suitable.*
☒	☐	For Bayesian analysis, information on the choice of priors and Markov chain Monte Carlo settings
☒	☐	For hierarchical and complex designs, identification of the appropriate level for tests and full reporting of outcomes
☒	☐	Estimates of effect sizes (e.g. Cohen's *d*, Pearson's *r*), indicating how they were calculated

Our web collection on statistics for biologists contains articles on many of the points above.

Software and code

Policy information about availability of computer code

Data collection	NanoDrop 2000c 1.6 (ThermoFisher); LabSolutions 5.93, 5.97 and 5.113(Shimadzu); Esquire Control Version 6.2 (Bruker);TopSpin 2.1 (Bruker); SkanIt 2.4.5.9 (ThermoFisher); Omega 1.30 (BMG Labtech); Schrödinger Suite (Schrödinger Release 2020-2); Analyst 1.7.0 (AB Sciex).
Data analysis	LabSolutions 5.93 (Shimadzu); LabSolutions Insight 3.5 (Shimadzu); DataAnalysis 4.0 (Bruker) for MS; GraphPad Prism 8.0.1; iNMR 6.3.3 (Mestrelab Research); Sparky 3.114; Discovery Studio Suite 18.1.0.17334 (Dassault Systemes); Maestro GUI 12.4.072 (Schrödinger); WinNonlin 6.3 (Pharsight Corp.); Analyst 1.7.0 (AB Sciex); UCSF ChimeraX 1.6rc202304272016.

For manuscripts utilizing custom algorithms or software that are central to the research but not yet described in published literature, software must be made available to editors and reviewers. We strongly encourage code deposition in a community repository (e.g. GitHub). See the Nature Portfolio guidelines for submitting code & software for further information.

Data

Policy information about availability of data

All manuscripts must include a data availability statement. This statement should provide the following information, where applicable:

- Accession codes, unique identifiers, or web links for publicly available datasets
- A description of any restrictions on data availability
- For clinical datasets or third party data, please ensure that the statement adheres to our policy

Source data are provided as a Source Data file. All other data are available in the supplementary information files. CryoEM structure of somatostatin receptor 2 in complex with Octreotide (7T11) used for the docking study is available in the Protein Data Bank.

Research involving human participants, their data, or biological material

Policy information about studies with human participants or human data. See also policy information about sex, gender (identity/presentation), and sexual orientation and race, ethnicity and racism.

Reporting on sex and gender	Blood samples were collected from three healthy participants, one female and two male. Study duration: one year. From each individual blood was collected twice over the entire duration of one year.
Reporting on race, ethnicity, or other socially relevant groupings	The participants were not selected based on race, ethnicity or socially relevant criteria.
Population characteristics	Fresh human blood was collected from three 26-28 year old healthy donors without medical history.
Recruitment	Plasma samples were exclusively donated by three coauthors of the manuscript. Blood samples were collected by qualified personnel at the Health Center of the Indian Institute of Science, Bangalore. All participants gave written informed consent before contributing blood samples.
Ethics oversight	The study was reviewed and approved by the Institutional Human Ethics Committee (IHEC) of the Indian Institute of Science, Bangalore. (Approval number: IHEC: 4-14032018)

Note that full information on the approval of the study protocol must also be provided in the manuscript.

Field-specific reporting

Please select the one below that is the best fit for your research. If you are not sure, read the appropriate sections before making your selection.

- [x] Life sciences
- [ ] Behavioural & social sciences
- [ ] Ecological, evolutionary & environmental sciences

For a reference copy of the document with all sections, see nature.com/documents/nr-reporting-summary-flat.pdf

Life sciences study design

All studies must disclose on these points even when the disclosure is negative.

Sample size	Sample sizes were chosen based on preliminary experiments. All experiments were replicated twice or thrice for clear interpretation of the data.
Data exclusions	No data was excluded from analysis.
Replication	Unless otherwise mentioned, all assays were performed independently with n = 3. All attempts at replication were successful and the deviation in the measurements are depicted in the graphs.
Randomization	Randomization is not relevant to this study.
Blinding	No blinding was done in the assays for clear interpretation and analysis of the data.

Reporting for specific materials, systems and methods

We require information from authors about some types of materials, experimental systems and methods used in many studies. Here, indicate whether each material, system or method listed is relevant to your study. If you are not sure if a list item applies to your research, read the appropriate section before selecting a response.

Materials & experimental systems

n/a	Involved in the study	
[ ]	[x]	Antibodies
[ ]	[x]	Eukaryotic cell lines
[x]	[ ]	Palaeontology and archaeology
[ ]	[x]	Animals and other organisms
[x]	[ ]	Clinical data
[x]	[ ]	Dual use research of concern
[x]	[ ]	Plants

Methods

n/a	Involved in the study	
[x]	[ ]	ChIP-seq
[x]	[ ]	Flow cytometry
[x]	[ ]	MRI-based neuroimaging

Antibodies

Antibodies used	Rat Growth Hormone ELISA Kit (Invitrogen), Cat. No. KRC5311.
Validation	The Biotinylated detection antibody was used as provided by the manufacturer in the assay kit.

Eukaryotic cell lines

Policy information about cell lines and Sex and Gender in Research

Cell line source(s)	Caco-2 (NCCS Pune, India); HEK293T (NCCS, Pune, India).
Authentication	Caco-2 cell line was authenticated by STR (short tandem repeat) profiling. HEK293T cells were not authenticated and used as supplied.
Mycoplasma contamination	Caco-2 and HEK293T cell lines tested negative for mycoplasma contamination.
Commonly misidentified lines (See ICLAC register)	No commonly misidentified cell lines were used in the study.

Animals and other research organisms

Policy information about studies involving animals; ARRIVE guidelines recommended for reporting animal research, and Sex and Gender in Research

Laboratory animals	Male Wistar IGS rats (Rattus norvegicus) of 9 weeks old were procured from Hylasco Biotechnology Pvt Ltd., Hyderabad, India. Sprague-Dawley rats (Rattus norvegicus) of male sex 6 weeks old were procured from CPCSEA registered, Biogen Laboratory Animal Facility (Bangalore, India).
Wild animals	No wild animals were used in the study.
Reporting on sex	Rats of male sex were considered to allow for clear data interpretation. Female rats were excluded in the experiments to avoid data variability resulting from the estrous cycle.
Field-collected samples	No field collected samples were used in the study.
Ethics oversight	The animal experiments were performed at animal facility of Anthem Biosciences Pvt. Ltd. and at the Central Animal Facility, Indian Institute of Science, Bangalore, following CPCSEA (The Committee for the Purpose of Control and Supervision of Experiments on Animals) and ARRIVE guidelines. The work plans were reviewed and approved by the Institutional animal ethics committee of Anthem Biosciences Pvt. Ltd. and the Indian Institute of Science, Institute Animal Ethical Committee (IAEC). (Approval number: IAEC: CAF/ Ethics/678/2019).

Note that full information on the approval of the study protocol must also be provided in the manuscript.
